# A genomic approach to analyze the cold adaptation of yeasts isolated from Italian Alps

**DOI:** 10.3389/fmicb.2022.1026102

**Published:** 2022-11-08

**Authors:** Benedetta Turchetti, Pietro Buzzini, Marcelo Baeza

**Affiliations:** ^1^Department of Agricultural, Food and Environmental Sciences, and Industrial Yeasts Collection DBVPG, University of Perugia, Perugia, Italy; ^2^Departamento de Ciencias Ecológicas, Facultad de Ciencias, Universidad de Chile, Santiago, Chile

**Keywords:** cold-adapted yeasts, Alpine yeasts, draft genomes, stress genes, secondary metabolites

## Abstract

Microorganisms including yeasts are responsible for mineralization of organic matter in cold regions, and their characterization is critical to elucidate the ecology of such environments on Earth. Strategies developed by yeasts to survive in cold environments have been increasingly studied in the last years and applied to different biotechnological applications, but their knowledge is still limited. Microbial adaptations to cold include the synthesis of cryoprotective compounds, as well as the presence of a high number of genes encoding the synthesis of proteins/enzymes characterized by a reduced proline content and highly flexible and large catalytic active sites. This study is a comparative genomic study on the adaptations of yeasts isolated from the Italian Alps, considering their growth kinetics. The optimal temperature for growth (OTG), growth rate (Gr), and draft genome sizes considerably varied (OTG, 10°C–20°C; Gr, 0.071–0.0726; genomes, 20.7–21.5 Mpb; %GC, 50.9–61.5). A direct relationship was observed between calculated protein flexibilities and OTG, but not for Gr. Putative genes encoding for cold stress response were found, as well as high numbers of genes encoding for general, oxidative, and osmotic stresses. The cold response genes found in the studied yeasts play roles in cell membrane adaptation, compatible solute accumulation, RNA structure changes, and protein folding, i.e., dihydrolipoamide dehydrogenase, glycogen synthase, omega-6 fatty acid, stearoyl-CoA desaturase, ATP-dependent RNA helicase, and elongation of very-long-chain fatty acids. A redundancy for several putative genes was found, higher for P-loop containing nucleoside triphosphate hydrolase, alpha/beta hydrolase, armadillo repeat-containing proteins, and the major facilitator superfamily protein. Hundreds of thousands of small open reading frames (SmORFs) were found in all studied yeasts, especially in *Phenoliferia glacialis.* Gene clusters encoding for the synthesis of secondary metabolites such as terpene, non-ribosomal peptide, and type III polyketide were predicted in four, three, and two studied yeasts, respectively.

## Introduction

Studies on the strategies exhibited by microbial communities inhabiting cold regions worldwide to thrive at low temperatures have increased in recent decades. Cold environments, characterized by temperatures below 5°C, predominate on Earth and represent a harsh habitat for microbial life leading to a strong reduction of growth rates (Feller and Gerday, 2003; D’Amico et al., 2006; Margesin and Feller, 2010; Margesin and Miteva, 2011). Cold-adapted (i.e., psychrophilic) microorganisms have evolved molecular and metabolic adaptations to overcome the adverse effects of low temperatures, including the synthesis of cryoprotectant molecules, cold-active enzymes, and polyunsaturated lipids regulating the degree of membrane fluidity (Margesin et al., 2007; Buzzini and Margesin, 2013; Alcaíno et al., 2015; Baeza et al., 2017). An increase in antioxidant enzymes activities was observed in Antarctic fungi after incubation at low temperatures, suggesting that the antioxidant defense could play a significant role in microbial survival when subjected to cold conditions (Gocheva et al., 2006, 2009; Kostadinova et al., 2012). When the Antarctic yeast *Mrakia blollopis* was submitted to a cold-shock an accumulation of TCA-cycle metabolites, lactic acid, and polyamines was observed (Tsuji, 2016). Polyamines are recognized to regulate important functions in cell growth, development, and cellular protection against stresses (Valdés-Santiago and Ruiz-Herrera, 2013). In the genome of the cold-adapted yeast *Mrakia psychrophila* there is a high numbers of genes encoding for Major Facilitator Superfamily (MFS) proteins (133), which play multiple roles including the incorporation of nutrients from the environment (Perlin et al., 2014; Su et al., 2016), and suggested as an adaptation to survive in its environment. Considering the protein 3D structural level, the highly flexible and larger active site exhibited by cold-active enzymes is considered responsible for their higher specific activity in comparison to their mesophilic counterparts (Collins et al., 2008; Jung et al., 2008; Feller, 2013). The reduced proline content found in bacterial cold-active proteins has been proposed as an adaptation strategy to attenuate the negative effect of proline isomerization on protein folding (Wedemeyer et al., 2002; Feller and Gerday, 2003; Feller, 2010).

OMICs technologies have become practical approaches for studying microorganisms, opening a window to find novel and holistic strategies of cold adaptation expressed by microbial cells (Casanueva et al., 2010). A higher number of rRNA and tRNA genes have been found in psychrophilic than mesophilic bacterial genomes suggesting an adaptation strategy to compensate the reduced translation rate under low-temperature conditions (Médigue et al., 2005; Methé et al., 2005; Riley et al., 2008; Choi et al., 2020). However, in the few studies about genomes of psychrophilic yeasts the number of rRNA and tRNA genes was variable and not consistently higher than mesophilic yeasts (Firdaus-Raih et al., 2018; Baeza et al., 2021; Yusof et al., 2021).

Genes associated with cold tolerance were found in genomes of the yeast *Naganishia vishniacii* isolated from McMurdo Dry Valleys, Antarctica; putative genes encoding for solute transfers, chaperones, associated with photoprotection, desaturases, trehalose synthesis, and lipid metabolism were found (Nizovoy et al., 2021). The yeast genus *Glaciozyma* includes psychrophilic species isolated from Antarctica and European glaciers, comprising some species formerly classified into different genera (e.g., *Leucosporidium antarcticum*, which has been re-classified as *Glaciozyma antarctica*; Turchetti et al., 2011). Genes encoding for proteins stimulating cold survival, e.g., antifreeze proteins (AFPs), were identified in the genome of *G. antarctica* PI12 (Wong et al., 2019). The comparisons with other genomes of two mesophilic yeasts, two thermophilic, and one psychrophilic fungi revealed that a third of the genes identified in *G. antarctica* were absent in the more closely related yeasts, suggesting that these genes, which are unique for *G. antarctica*, could be associated to its psychrophilic habitus (Firdaus-Raih et al., 2018). Furthermore, a 4% of the annotated open reading frames (ORFs) found in the *G. antarctica* genome corresponded to small ORFs (smORFs) not exceeding 100 amino acids, which may have a role in cold adaptation (Mat-Sharani et al., 2015). Thousands to millions of smORFs have been found in other organisms, including humans, insects (i.e., *Drosophila melanogaster*), metazoans, and yeasts (i.e., *Saccharomyces cerevisiae*), in which they fulfill key physiological functions (Couso and Patraquim, 2017).

The analysis of genomes and transcriptomes of a few Antarctic yeasts revealed the presence of a considerable number of putative genes involved in the synthesis of enzymes regulating the response to stress; most of them were classified as oxidative, general, and cold-associated responses, including desaturases, chaperones and chaperonins, glutathione S-transferase, catalase, and enzymes for the production and regulation of compatible osmolytes. Furthermore, putative genes involved in the synthesis of secondary metabolites, i.e., non-ribosomal peptide synthase (NRPS), terpenes, and type III polyketide synthases (PKSs) were found (Baeza et al., 2021, 2022). A global comparative analysis of *in silico* generated proteomes among Antarctic yeasts in comparison with their different optimal temperature for growth (OTG) and growth rates (Gr), revealed that a general trend of high content of flexible proteins associated with low OTG was found in yeasts characterized by fast growth. When subjected to cold stress, yeasts characterized by similar growth parameters exhibited similar transcriptomic level (Baeza et al., 2022). Interestingly, a significant upregulation of genes encoding for heat-shock proteins and chaperones, fatty acid desaturases, and proteasomal subunits, associated with the downregulation of ribosomal subunits were observed in yeasts exhibiting the higher OTG (e.g., *Candida sake* and *Wickerhamomyces anomalus*), but not in those with lower OTG (e.g., *Mrakia gelida*, and *Cryptococcus* sp.). In contrast, some yeasts characterized by lower OTG showed an upregulation of ribosomal subunits, suggesting the presence of differences strategies of adaptation to cold depending on their specific OTG (Baeza et al., 2022). In *Rhodosporidium kratochvilovae* strain YM25235 subjected to low-temperature stress, 1,300 differentially expressed genes (DEGs) were detected by next-generation deep sequencing technology (RNA-seq). From them, the pathways included the MAPK signaling pathway, metabolic pathways, and amino sugar and nucleotide sugar metabolism were overrepresented (Guo et al., 2021). The MAP-Kinase HOG1 would play a role in cold adaptation in *R. kratochvilovae* by promoting biosynthesis of polyunsaturated fatty acids and glycerol (Chen et al., 2022).

In recent years, the genomes of cold adapted yeasts from Antarctica were sequenced and characterized; their diverse responses to cold adaptations were studied, analyzing genomes and transcriptomes (Wong et al., 2019; Baeza et al., 2021, 2022; Nizovoy et al., 2021; Touchette et al., 2022). The extension of these studies to cold-adapted yeasts isolated from other geographical areas, which are characterized by chemical–physical and ecological conditions (including a different annual and seasonal temperature range) very different from those of the Antarctic habitats, is essential to advance toward a global understanding of yeasts’ adaptative strategies to live in cold habitat worldwide. In this work, the genomes of four strains of different cold-adapted yeast species isolated from the Italian Alps and Apennines were sequenced, analyzed, and compared in relation to their different optimal growth temperatures. In this framework, a comparative analysis among yeasts was performed considering their growth properties, genome size, protein flexibility, and putative genes encoding for cold stress response and secondary metabolites.

## Materials and methods

### Yeast strains, culture conditions, determination of the optimum temperature and growth rates

The yeast strains used in the present study were *Goffeauzyma gilvescens* DBVPG 4707, *Glaciozyma martinii* DBVPG 4841, *Naganishia antarctica* DBVPG 5271, and *Phenoliferia glacialis* DBVPG 5880, all preserved in the Industrial Yeast Collection DBVPG of the University of Perugia, Italy. They were all isolated from soil or sediments of cold habitats associated to high mountain glaciers of Italian Alps and Apennines. More in-depth information on strains used in this study are available on the website of DBVPG Collection (www.dbvpg.unipg.it). All the strains were routinely maintained in physiological inactive state (−80°C). Working cultures were grown on YEPG (yeast extract 10 g L^−1^, peptone 10 g L^−1^, glucose 20 g L^−1^, agar 15 g L^−1^) agar slants at 25°C (*G. gilvescens*) or 15°C (*G. martinii*, *N. antarctica*, and *P. glacialis*).

Calibrated (A_600_ = 0.8, which was considered an average cell concentration = 10^7^ cells/mL) suspensions of 48 h-old cells of the 4 strains were used to inoculate 200 μl of YEPG into 96 wells microplate to obtain A_600_ = 0.1. Microplates were incubated at 4°C, 10°C, 15°C, 20°C, 25°C, and 30°C and A_600_ was checked every day for 10 days. Growth curves were determined in triplicate; the growth rates (Gr) of each strain were calculated from the exponential phase of the growth curves while, the temperature in which the highest A_600_ during exponential phase was observed was considered the optimum temperature for growth (OTG).

### Genomic DNA purification

DNA Was extracted using standard methods based On The treatment of yeasts cells with CTAB (hexadecyltrimethylammonium bromide) and chloroform/isoamyl alcohol buffers. Briefly, 72 h yeast cells were suspended In pre-warmed CTAB extraction buffer containing CTAB, NaCl, EDTA, Tris HCl, PVP (Polyvinylpyrrolidone), β-mercaptoethanol, and proteinase K (sigma, Aldrich) and incubated at 60°C for 1 h. then chloroform/isoamylalcohol (24:1) Solution Was added and The Mix Was centrifuged To recover The aqueous phase. The DNA Was then purified using pectinase and RNAase (sigma, Aldrich) incubating The Mix at 37°C, and phenol:Chloroform:Isoamylalcohol (25:24:1; Sigma, Aldrich). After centrifugation, DNA contained In The aqueous phase Was additionally washed with sodium acetate (3 M), isopropanol and ethanol 70% (sigma, Aldrich). After an additional centrifugation, DNA Was suspended In ultrapure water and maintained at −20°C until quantification and sequencing The DNA Was quantified By qubit Fluorometric assay (ThermoFisher scientific) To obtain a concentration higher than 20 ng/μl.

### Next-generation sequencing

NGS by the Omics2view company (Germany, http://www.omics2view.consulting) on MGI DNBSEQ-G400 in 2 × 150 bp mode, including sequence processing as follow. Quality of demultiplexed reads was checked with FastQC v0.11.7 (Andrews, 2018). A summary QC report created with MultiQC v1.10 (Ewels et al., 2016) is available for download. Read quality trimming was performed with the BBTools package v38.45 (Bushnell, 2019). This comprised the removal of optical duplicates, human sequences, adapter sequences, low entropy reads, and trimming of bases with quality scores <20. Reads with invalid or ambiguous bases and reads with a length < 50 base pairs (bp) were discarded. Only reads surviving quality trimming as pairs entered downstream analysis. Read quality recalibration and error correction was performed with the BBTools package v38.45 (Bushnell, 2019). Quality-trimmed reads were aligned to a preliminary de-novo assembly made with Tadpole from a subset of the quality-trimmed reads. Alignment information was used to recalibrate the base quality of all quality-trimmed reads. Sequencing errors were corrected by consecutively applying BBTools programs BBMerge, Clumpify, and Tadpole in error correction mode on the quality-recalibrated reads. Results are referred to as “filtered reads.” 31-bp kmers of filtered reads were normalized with BBNorm from the BBTools package v38.45 (Bushnell, 2019) to a target kmer depth of 100×, with a minimum kmer depth of 3×. Contiguous sequences (contigs) were assembled from normalized reads and further combined to Scaffolds ≥500 bp with SPAdes v3.15.2 (Bankevich et al., 2012) using k-mer lengths up to 127 bp. The genome completeness was evaluated by BUSCO v.5.0 (Nishimura et al., 2017, 2019) using the Fungi datasets.

Draft genomes have been deposited at DDBJ/ENA/GenBank under the following accession numbers and versions: *G. gilvescens* DBVPG 4707: JANGFI000000000; *G. martinii* DBVPG 4841: JANGFH000000000; *N. antarctica* DBVPG 5271: JANGFJ000000000; *P. glacialis* DBVPG 5880: JANHKM000000000.

### Prediction of secondary metabolite biosynthetic clusters

The draft genomes were submitted to antiSMASH 6 fungal version to predict biosynthesis of secondary metabolites clusters in strict mode using default parameters (Blin et al., 2019). Each predicted core biosynthetic gene (CBG) and additional biosynthetic gene (ABG) of different gene clusters was compared to NCBI nr database by Blastp using default parameters, and the complete sequence of first 10 hits were retrieved. The sequences were analyzed using the maximum likelihood method and JTT matrix-based model (Jones et al., 1992) using the MEGA11 software (Kumar et al., 2018; Stecher et al., 2020). Initial tree(s) for the heuristic search were obtained automatically by applying Neighbor-Join and BioNJ algorithms to a matrix of pairwise distances estimated using a JTT model and then selecting the topology with a superior log-likelihood value.

### Prediction, annotation, and comparative analysis of coding sequence

Bioinformatic analysis was performed using Geneious Prime 2020.1.2 software (Kearse et al., 2012). The CDSs with lengths ≥210 nt were predicted in draft genomes using the generic training of the Augustus gene prediction program (Stanke and Morgenstern, 2005; Stanke and Tzvetkova, 2006), trough the Geneious Augustus plugin. The CDS were *in silico* extracted, translated, and compared by Blastp against a local curated fungal protein database (updated in June 2021). The hits having at least 30% similarity and *E* values ≤10^−10^ were considered for annotation. The annotated CDSs were classified according to cellular function predicted using KAAS – KEGG Automatic Annotation Server (Moriya et al., 2007)^1^ trough GHOSTX, and using default parameters and the datasets for Genes and Eukaryotes.

The translated and annotated CDS were used for comparative analysis of their flexibilities calculated based on their sequences of amino acids. For that, the percentage of amino acids classified according to their flexibility index (Li et al., 2006; Rao et al., 2011) either as very flexible (Vf: E, G, K, N, Q, and S) or moderately flexible (Mf: A, D, H, I, P, R, T, V) were calculated for each CDS. Furthermore, the global flexibility of each CDS was predicted using MEDUSA, a Deep-Learning based protein flexibility tool that uses as input the information from homologous protein sequences and amino acid physicochemical properties, using default parameters (Vander Meersche et al., 2021). From that, the percentage of residues grouped in three predicted flexibility classes was considered, M0, M1, and M2, from rigid to flexible. For each CDS the percentages of amino acids grouped as Vf, Vf plus Mf (VMf), M2, and M1 + 2, were calculated. The comparisons among yeasts were performed globally, considering the results for all CDS, and using the CDSs classified by cellular function by applying one-way ANOVA followed by a *post hoc* Tukey analysis. The data distribution was visualized using histograms and quantile-quantile (QQ) plots and did not find any indication that the data did not have a normal distribution. The CDS that displayed significant differences (significance threshold of 0.05) in their calculated flexibilities in the comparisons among yeasts at the global level or grouped by cellular function were selected to evaluate a probable correlation to yeast’s growth parameters. For that, the differences among yeasts in calculated flexibilities of CDS were plotted vs. their differences in the optimal temperature for growth or growth rate, and linear regressions were applied.

### Prediction of small open reading frames

The ORFs between 30 and 300 nt were predicted in the draft genomes using the ORF finder included in Geneious software, annotated as SmORFs, and classified as those present in the CDS (CDSSmORFs), into 1,000 nt upstream (5’SmORFs) and into 1,000 nt downstream (3’SmORFs) from the CDS. SmORFs were translated and annotated by Blastp comparison to a local SmOrfs database constructed with the data downloaded from the web pages smORFunction^2^ (Ji et al., 2020) and SmProt^3^ (Hao et al., 2018; Li et al., 2021).

### Multigene phylogeny

This analysis was conducted to compare the *P. glacialis* isolates from the Italian Alps and Antarctica and other members belonging to order Kriegeriales for which the genomes are available in the NCBI database were included. The corresponding genomes were downloaded (Supplementary Table S1), and the CDS were predicted and annotated, as mentioned above. The CDS encoding for putative proteins common to at least eight yeasts, including both *P. glacialis* isolates, were selected for more analysis. Furthermore, the analysis was performed for SmORFs. The translated SmORFs with at least 30% similarity in Blastp comparison against the local SmORF database mentioned above and common to three and all the studied yeasts were considered. The reference SmORF sequences were also included. The Evolutionary distances were computed for the individual proteins and concatenated CDS using the Jukes-Cantor method; the phylogenetic trees were obtained by neighbor-joining with 1,000 bootstrap replications. The analyses were conducted in MEGA11 software (Kumar et al., 2018; Stecher et al., 2020).

## Results

### Yeast growth parameters

**Figure 1:**
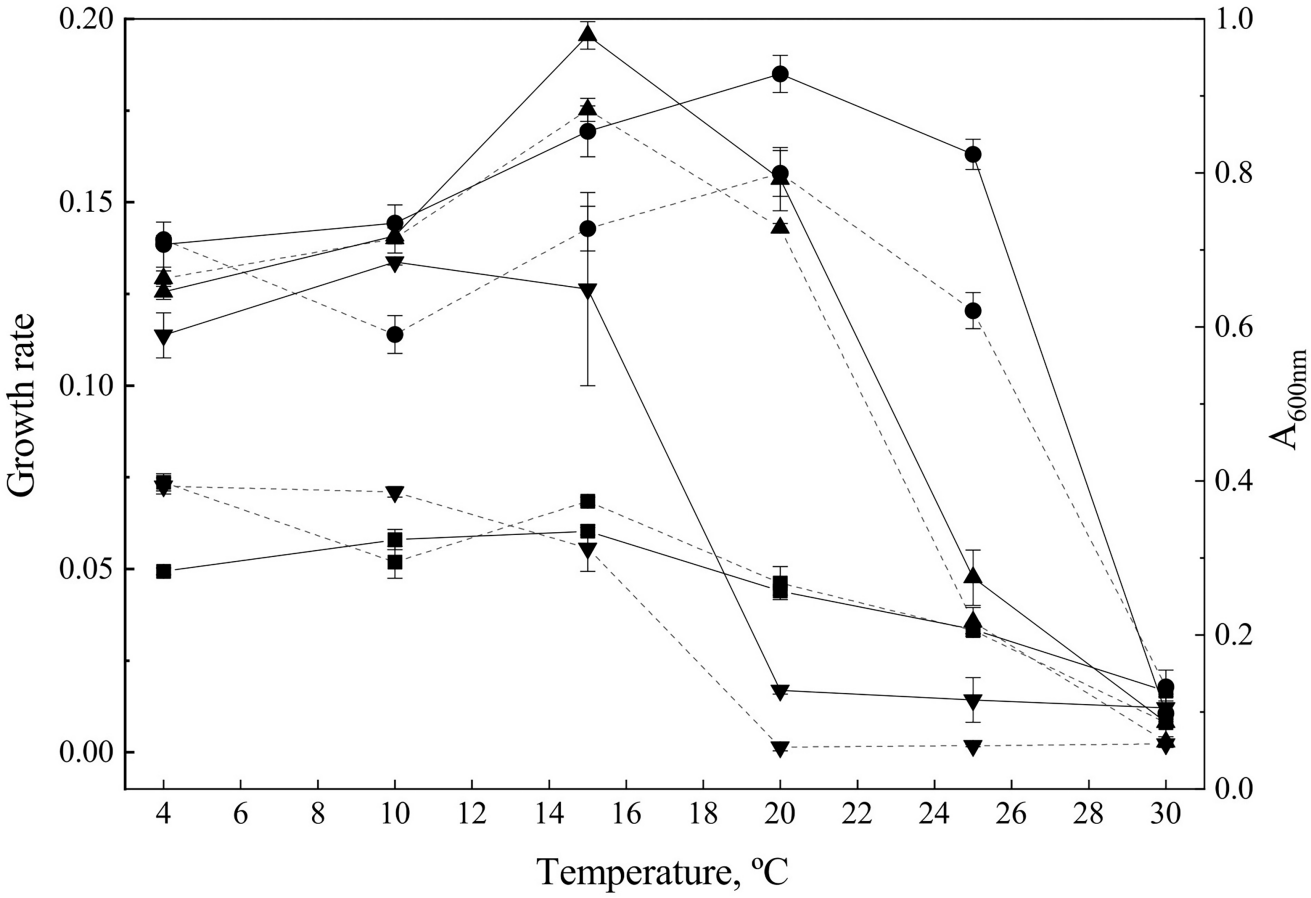
Growth parameters. Growth curves were determined at temperatures from 4°C to 30°C. The growth rate (discontinuous lanes) was calculated from the exponential phase of the growth curves. Continuous lanes show to the maximum A_600_ observed during exponential phase at different temperatures and express the optimal temperature for growth for each strain. *Goffeauzyma gilvescens* (circle), *Glaciozyma martinii* (down triangle), *Naganishia antarctica* (square), and *Phenoliferia glacialis* (up triangle).

The complete growth curve was determined for each strain at temperature ranging from 4°C to 30°C, to obtain both OTG and Gr (Figure 1).

*G. martinii* showed the lowest OTG (at 10°C), and the most limited temperature growth range (from 4°C to 15°C). *N. antarctica* and *P. glacialis* exhibited similar OTG at 15°C while *G. gilvescens* showed the highest OTG (20°C). All the strains exhibited growth at 4°C while only *G. gilvescens* and *P. glacialis* grew at 25°C. No growth was observed at 30°C (Figure 1).

The maximum Gr was showed by *P. glacialis* at 15°C (0.175 ± 0.003) while for *G. gilvescens* the maximum Gr was recorded at 20°C (0.1579 ± 0.0062).

The maximum Gr of *G. martinii* and *N. antarctica* corresponds to a value more than twice lower than the Gr of the other two species and were exhibited at 4°C–10°C with no significant differences for *G. martinii* (0.0726 ± 0.0022 and 0.0710 ± 0.0014, respectively) and at 4°C for *N. antarctica* (0.0736 ± 0.0023; Figure 1).

### Draft genomes, prediction, and annotation of CDS, and secondary metabolite biosynthetic clusters

The contigs assembled in draft genomes ranged from 401 in *N. antarctica* to 1,908 in *G. gilvescens*, the size was from 20.7 Mpb in *G. gilvescens* to 33.8 Mpb in *P. glacialis*, and the GC content was from 50.9% in *G. gilvescens* to 61.2% in *P. glacialis* (Supplementary Table S2). The predicted CDS varied from 2,221 in *G. gilvescens* to 10,303 in *P. glacialis*, while the annotated ones ranged from 11% in *P. glacialis* to 20% in *G. gilvescens*. Secondary metabolite biosynthetic gene clusters were predicted in the draft genomes: terpene clusters in the four yeasts, type III polyketide synthase clusters (T3PKS) in *G. gilvescens* and *N. antarctica*, and non-ribosomal peptide synthetase clusters (NRSP) in *G. gilvescens*, *G. martinii* and *P. glacialis* (Table 1). The identification of protein encoded by core biosynthetic gene (CBG), additional biosynthetic gene (ABG), and transport related gene (TRG) in the clusters was inferred by a phylogenetic analysis that included the sequences of the best five Blastp hits for each sequence obtained from the NCBI database (Supplementary Figure S1). In terpene clusters, the CBG was closest to squalene synthase in *G. martinii* (seq 22,166) and *P. glacialis* (seq 24,553), to bifunctional farnesyl-diphosphate farnesyl transferase/squalene synthase in *G. gilvescens* (seq 14,972) and *N. antarctica* (seq 22,146), and to geranylgeranyl-diphosphate geranylgeranyl transferase, terpenoid cyclase, squalene/phytoene synthase and phytoene desaturase in *G. gilvescens* (seq 7,405), *G. martinii* (seq 17,625), *N. antarctica* (seq 20,744) and *P. glacialis* (seq 22,851), respectively. The ABG was close to amino acid transporter in *N. antarctica* and NAD-binding protein and NAD-dependent mannitol dehydrogenase in *P. glacialis* (seq 22,851 and 24,553, respectively). In T3PKS clusters the CBG found in *G. gilvescens* and *N. antarctica* apparently corresponded to chalcone synthase, and in *N. antarctica* the CBG and TRG were closest to anthranilate synthase and sugar transporter, respectively. The CBG of NRPS clusters predicted would correspond to nonribosomal peptide synthetase in *G. martinii* and *P. glacialis*, and to L-aminoadipate-semialdehyde dehydrogenase in *G. gilvescens*. The ABG of NRPS predicted in *P. glacialis* was close to an oxidoreductase (Supplementary Figure S1).

**Table 1:** Secondary metabolite biosynthesis clusters predicted on draft genomes: non-ribosomal peptide synthetase clusters (NRSP), type III polyketide synthase clusters (T3PKS), and terpene.

Type	Yeast	Length	CBG	ABG	TRG	OG
NRPS	*G. gilvescens*	13,057	L-aminoadipate-semialdehyde dehydrogenase			1
	*G. martinii*	4,488	Nonribosomal peptide synthetase			
	*P. glacialis*	29,508	Nonribosomal peptide synthetase	Oxidoreductase		4
T3PKS	*G. gilvescens*	6,344	Chalcone synthase			1
	*N. antarctica*	41,667	Chalcone synthase	Anthranilate synthase	Sugar transporter	11
Terpene	*G. gilvescens*	7,405	Geranylgeranyl-diphosphate geranylgeranyltransferase			1
	*G. gilvescens*	14,972	Bifunctional farnesyl-diphosphate farnesyl transferase/squalene synthase			2
	*G. martinii*	22,166	Squalene synthase			4
	*G. martinii*	17,625	Terpenoid cyclase			6
	*N. antarctica*	22,146	Bifunctional farnesyl-diphosphate farnesyl transferase/squalene synthase	Amino acid transporter		2
	*N. antarctica*	20,744	Squalene/phytoene synthase			6
	*P. glacialis*	22,851	Phytoene desaturase	NAD-binding		9
	*P. glacialis*	24,553	Saqualene synthase	NAD-dependent mannitol deshydrogenase		3

CBG, Core Biosyntetic Gene; ABG, Additional Biosynthetic Gene; TRG, Transport Related Gene; OG, Other Genes.

### Modules and metabolic pathways

**Figure 2:**
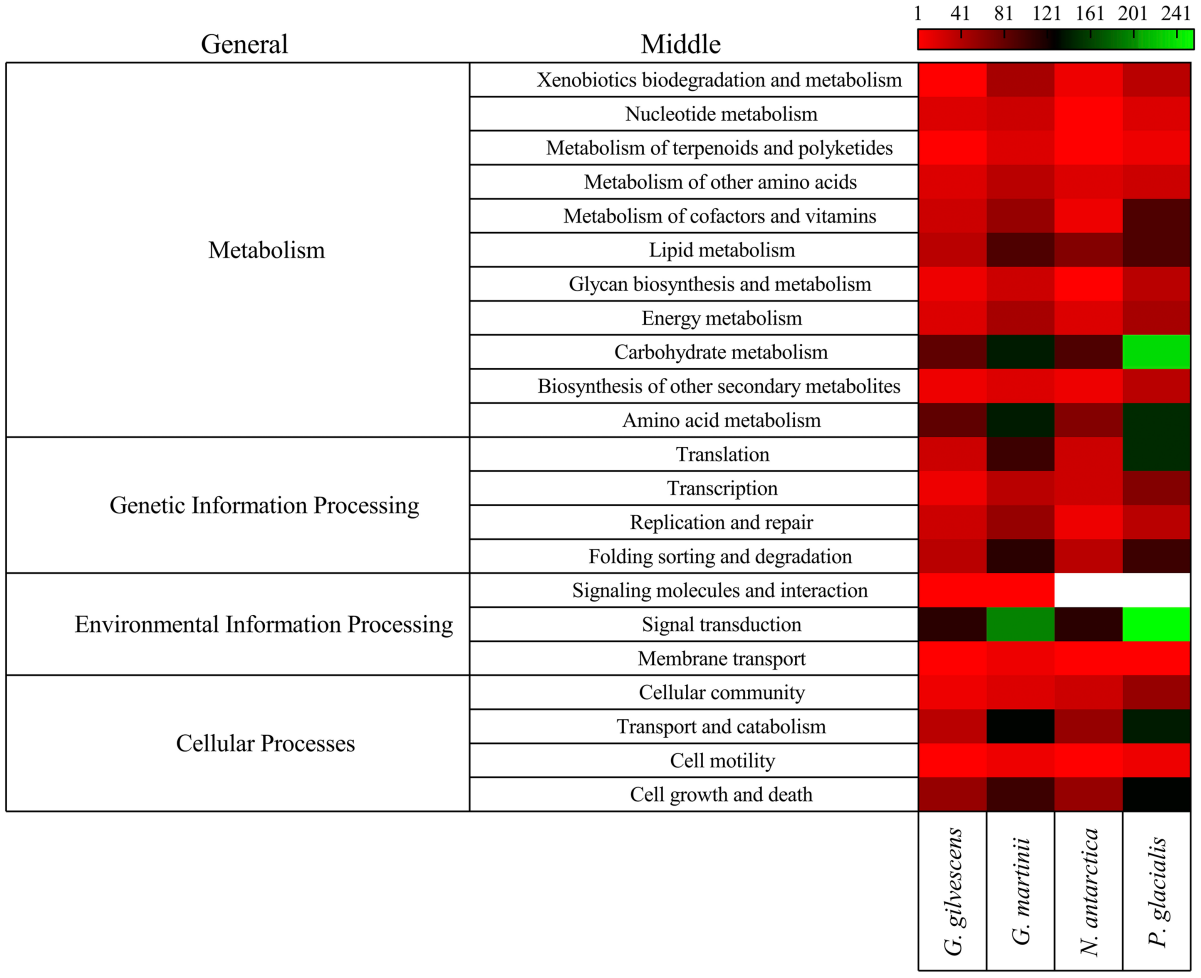
Classification of CDS by metabolic pathways. The CDSs were classified by comparison using GHOST X tools of the KAAS-KEGG Automatic Annotation Server. The number of CDSs grouped according to general and middle pathways are shown.

The annotated CDSs were classified according to general and specific completed modules and metabolic pathways. Forty-four modules were found, with the highest number in *P. glacialis* followed in decreasing order by *N. antarctica*, *G. martinii*, and *G. gilvescens* (Supplementary Table S3), 28 were found in the four studied yeasts and 9 in tree yeasts (i.e., Polyamine biosynthesis, arginine, assimilatory sulfate reduction and beta-oxidation acyl-CoA synthesis). The modules for which more putative genes were found were citrate cycle, beta-oxidation, leucine degradation, citrate cycle second carbon oxidation, and glycolysis. Seven modules associated to metabolism of cofactors and vitamins (i.e., lipoic acid biosynthesis, biotin biosynthesis and ubiquinone biosynthesis) were found only in two yeasts. Regarding metabolic pathways, more putative genes were found for *P. glacialis* and *G. martinii*, and the top5 at the middle level were signal transduction, carbohydrate metabolism, amino acid metabolism, transport and catabolism, and cell growth and death (Figure 2). At the specific level the top5 pathways were cell cycle, MAPK signaling pathway, spliceosome, ribosome, and autophagy (Supplementary Table S4). Of 201 specific pathways for which at least one putative gene was found, about the half were common to four yeasts, whiles 32 were found only in one yeast, most in *G. martinii* such as nitrogen metabolism, ether lipid metabolism, non-homologous end-joining, and atrazine degradation.

**Figure 3:**
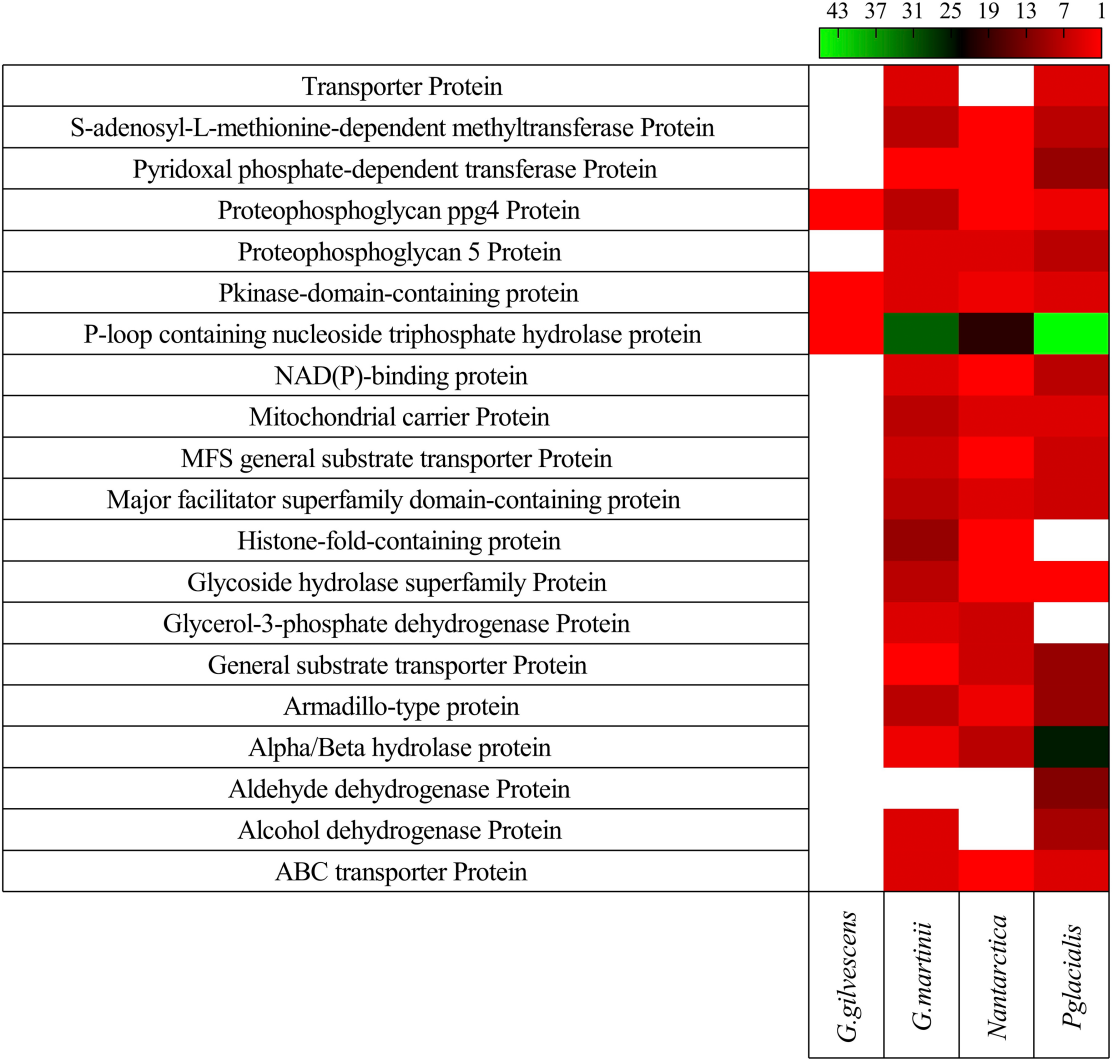
Gene redundancy for top 20 kinds of protein. The number of CDS predicted to encode for a protein in each yeast is shown.

In all studied yeasts, two or more CDS encoding for the same putative protein was found, especially in *G. martinii* and *P. glacialis*. The 20 putative proteins encoded by a higher number of CDS found in all yeasts are shown in Figure 3. Among them, P-loop containing nucleoside triphosphate hydrolase protein, alpha/beta hydrolase protein, armadillo-type protein, major facilitator superfamily domain-containing protein, and general substrate transporter protein can be mentioned.

### Stress related genes

A total of 423 orthologous genes associated to response to different kind of stresses were found, most of them related to oxidative and cold stress, followed by general, osmotic, and heat stress responses, especially in *P. glacialis* and *G. martinii* (Supplementary Table S5). The top5 predicted proteins related to stress were mitogen-activated protein kinase 1, catalase, dihydrolipoamide dehydrogenase, glyceraldehyde 3-phosphate dehydrogenase, glycogen synthase kinase 3 beta, ribulose-phosphate 3-epimerase.

A total of 13 putative stress related proteins were commonly found in the four studied yeasts, i.e., enoyl-[acyl-carrier protein] reductase II, mitogen-activated protein kinase 1, eukaryotic translation initiation factor 2-alpha kinase 4, formate dehydrogenase, UTP--glucose-1-phosphate uridylyl transferase, glycogen synthase kinase 3 beta, heat shock 70 kDa protein, and phosphoenolpyruvate carboxy kinase (ATP). A total of 73 orthologous genes related to cold stress were predicted, with enoyl-[acyl-carrier protein] reductase II, mitogen-activated protein kinase kinase 1, glycogen synthase, omega-6 fatty acid desaturase/acyl-lipid omega-6 desaturase (Delta-12 desaturase), and stearoyl-CoA desaturase (Delta-9 desaturase) found in at least three yeasts. Putative antifreeze glycopeptide AFGP polyprotein and antifreeze protein were identified in *N. antarctica* and *P. glacialis*, respectively.

### Small open reading frames

**Figure 4:**
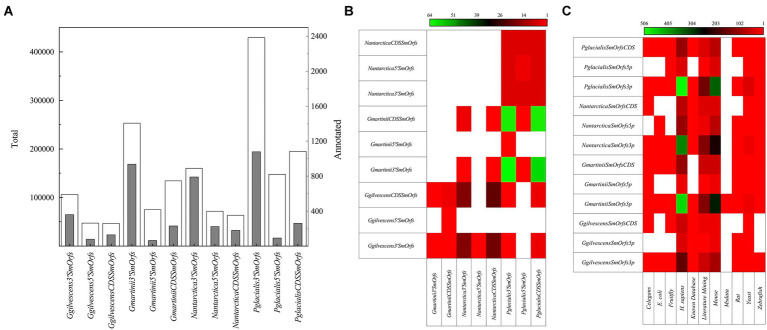
SmORFs prediction and comparison among yeasts. The total (white columns) SmORFs and those annotated (gray columns) by comparison SmOrfs database, are shown in **(A)** the number of translated SmORFs identical among yeasts is shown in **(B)** and those annotated by comparison to a SmORFs database in **(C)**. CDSSmORFs, SmORF located into a CDS; 5’SmORFs, SmORF located until 1,000 nt upstream of a CDS; 3’SmORFs, SmORF located until 1,000 nt downstream of a CDS (3’SmORFs).

Hundreds of thousands of SmORFs were found in all yeasts, in decreasing order in *P. glacialis* (772,037), *G. martinii* (462,525), *N. antarctica* (295,488), and *G. gilvescens* (199,985), and higher numbers of 3’SmOrfs were predicted (Figure 4A). No identical SmORFs nucleotide sequences were found among the studied yeasts, and very few considering translated sequences, being higher between *P. glacialis* and *G. martinii* in CDSSmOrfs and 3’SmOrfs (from 59 to 64 identities; Figure 4B). Compared to the SmORFs database, only 0.1%–0.5% of SmORFs yielded hits (considering at least 50% of coverage and similarity), most of them corresponding to the classifications *Homo sapiens* and mouse (Figure 4C). A phylogenetic analysis was performed considering the translated SmORFs with at least 30% similarity by Blastp to the SmORF database and common to three and all yeasts studied. The majority of common putative SmORFs corresponded to Literature mining classification. The yeasts were grouped close in either analysis of translated SmORFs common to three (Supplementary Figure S2A) and all yeasts (Supplementary Figure S2B), not observing a preference for hierarchical clustering among yeasts in any of the SmORF used for the analysis.

### Comparison of protein flexibilities based on amino acid composition

**Figure 5:**
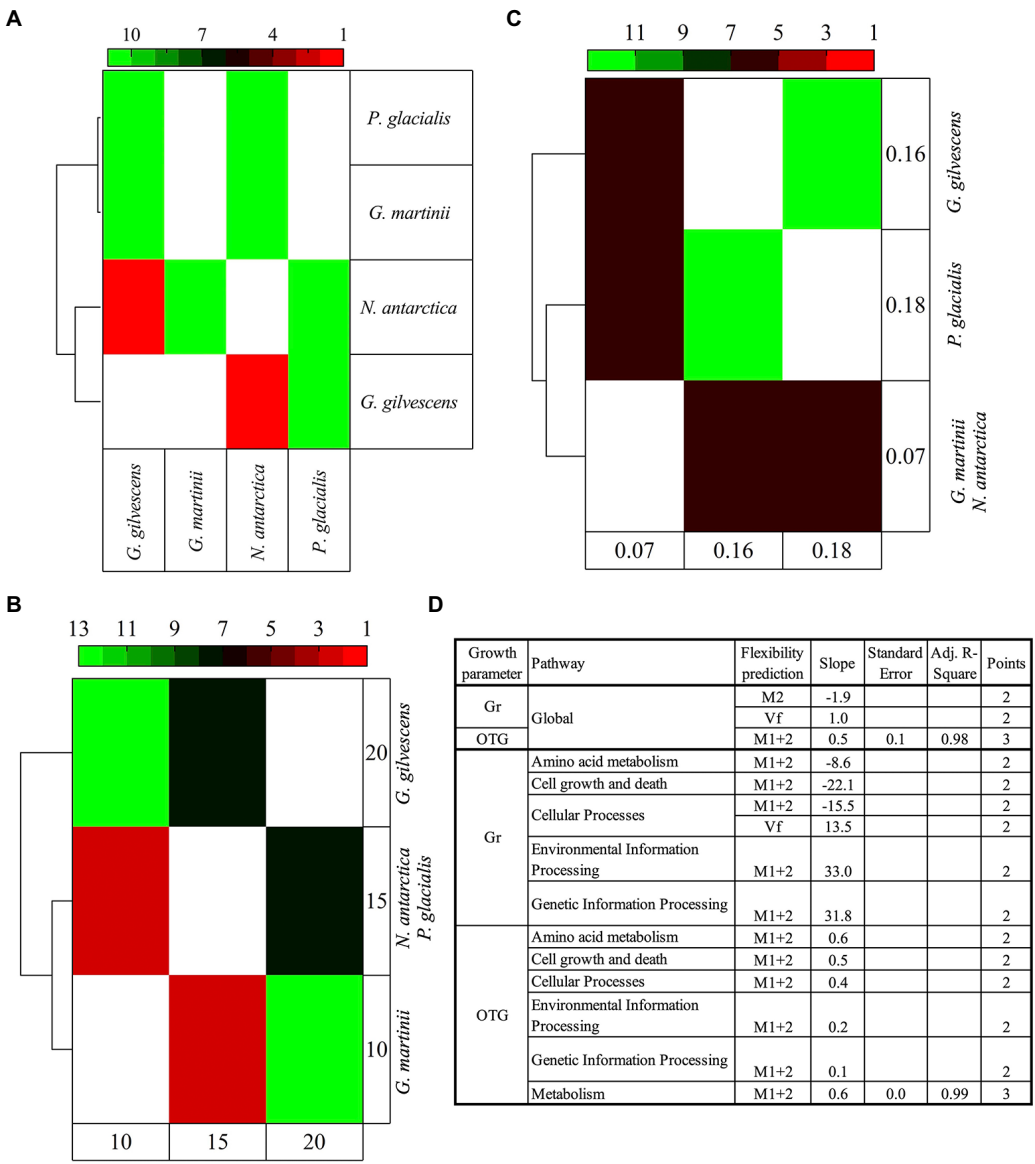
Comparative analysis of yeast species according to predicted protein flexibilities and growth parameters. The predicted flexibility of CDSs grouped by cellular functions were compared among species individually **(A)** and grouped according to their optimal temperature for growth **(B)** and growth rate **(C)**. The heatmap shows the number of cellular pathways for which significant difference (Tukey *post hoc* tests, *p* < 0.05) were found between yeasts. In **(D)** the plot of the difference between yeasts for a pathway in flexibility parameters (P1-2) vs. difference in growth parameters (F1-2) is shown. OTG, optimal temperature for growth; Gr, growth rate; Vf, very flexible; M2, class2 flexibility predicted by MEDUSA; M1 + 2, class 1 + class 2 flexibility predicted by MEDUSA.

The analysis was performed considering the predicted flexibilities of translated CDSs annotated and classified in cellular pathways. The M1 + 2 percentage showed the highest degree of significant (*p* < 0.05) variations among almost all yeasts under study, being higher between *G. martinii* vs. *G. gilvescens* and *N. antarctica*, and *P. glacialis* vs. *G. gilvescens* and *N. antarctica*. The pathways exhibiting more significant differences were carbohydrate metabolism, cell growth and death, and transduction signal. The clustering of species according to their differences revealed that *P. glacialis* was close to *G. martinii*, while *N. antarctica* was close to *G. gilvescens* (Figure 5A). When the 4 yeast species were grouped according to their OTG (obtaining 3 groups: OTG = 10°C, OTG = 15°C, and OTG = 20°C), those with OTG equal to 10°C and 15°C clustered separately from yeast exhibiting an OTG of 20°C (Figure 5B). On the other hand, when Gr was considered (Gr = 0.07, Gr = 0.16, Gr = 0.18), the yeast species showing either highest or lowest Gr clustered together, differently from those with intermediate Gr, which clustered separately (Figure 5C). The significant differences found among studied yeasts in CDS groups and parameters were analyzed concerning their differences in growth parameters (Figure 5D). Overall, positive, and negative correlations were obtained between Vf and M1 + 2, respectively, and Gr, while a positive correlation was found between M1 + 2 and OTG. In the analysis by pathways, both positive and correlations between M1 + 2 and Gr in 2 and 3 pathways, respectively, and positive correlation between M1 + 2 and OTG in 6 pathways were observed. These correlations were generally found in curves with only two points, except for M1 + 2 vs. OTG in for global and general metabolism pathway (*R*^2^ ≥ 0.98).

### Multigene phylogeny of Alpine and Antarctic *Phenoliferia glacialis* isolates

**Figure 6:**
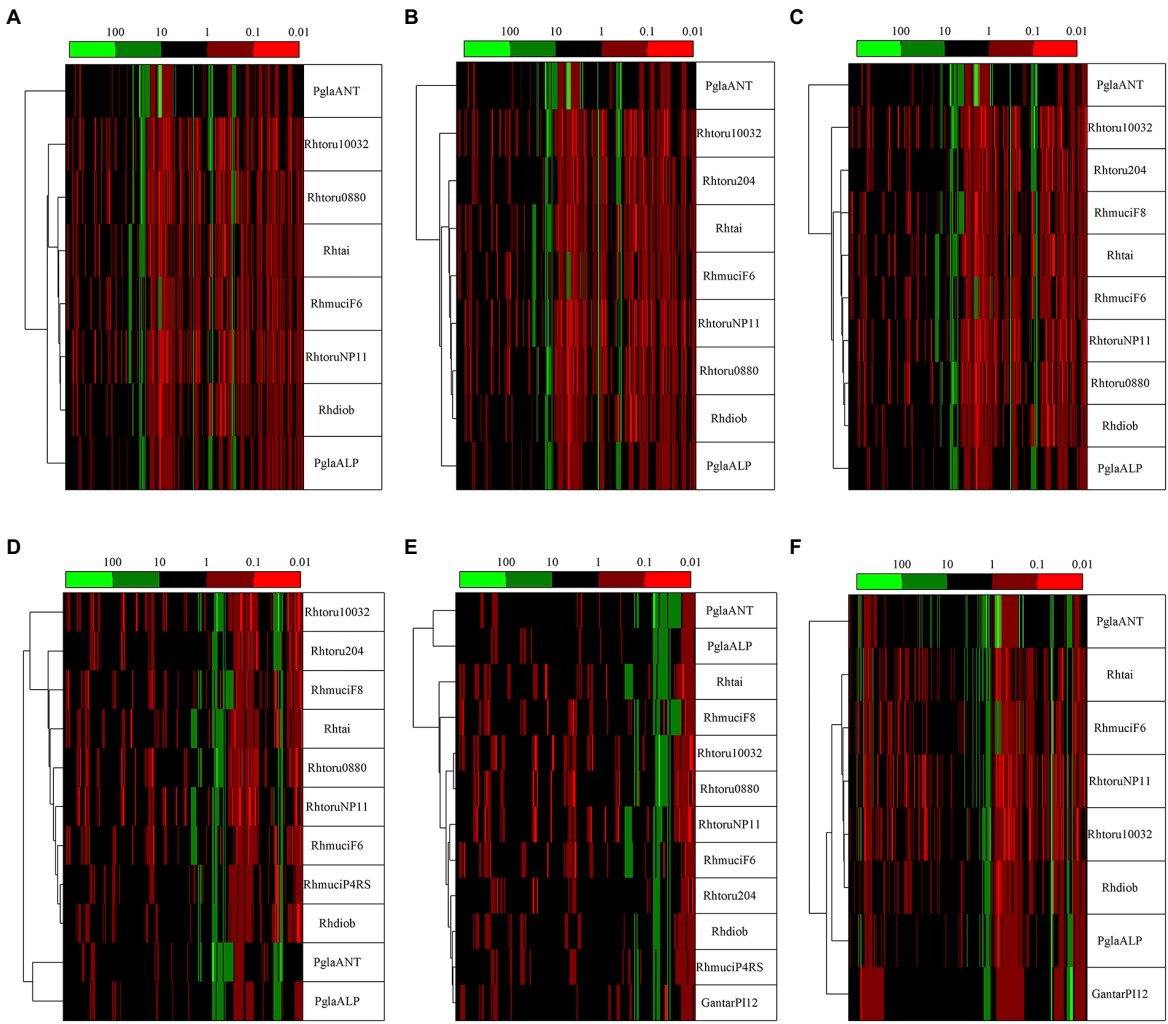
Comparison among yeasts based in phylogenetic analysis of individual proteins. Comparisons were made based on the patristic distances extracted from threes computed for proteins (Supplementary Figure S3) that were common to at least 8 yeasts, including both *P. glacialis* isolates. **(A)**, 50 proteins common to 8 yeasts; **(B)**, 41 proteins common to 9 yeasts; **(C)**, 35 proteins common to 10 yeasts; **(D)**, 23 proteins common to 11 yeasts; **(E)**, 24 proteins common to 12 yeasts. In **(F)**, was considered the proteins common at least for *P. glacialis* and *G. antarctica* (31 proteins common to 8 yeasts). PglaALP, *P. glacialis* isolate from Italian Alps; PglaANT, *P. glacialis* isolate from Antarctica; GantarPI12, *Glaciozyma antarctica* PI12; Rhdiob, *Rhodotorula diobovata* strain UCD-FST 08–225; RhmuciF6, *Rhodotorula mucilaginosa* strain F6; Rhmuci, *Rhodotorula mucilaginosa* strain F8; Rhhai, *Rhodotorula taiwanensis* strain MD1149; Rhtoru204, *Rhodotorula toruloides* ATCC 204091; RhtoruNP11C, *Rhodotorula toruloides* strain NP11C; Rhtoru10031, *Rhodotorula toruloides* strain NBRC10032; Rthoru0880, *Rhodotorula toruloides* strain NBRC 0880.

**Figure 7:**
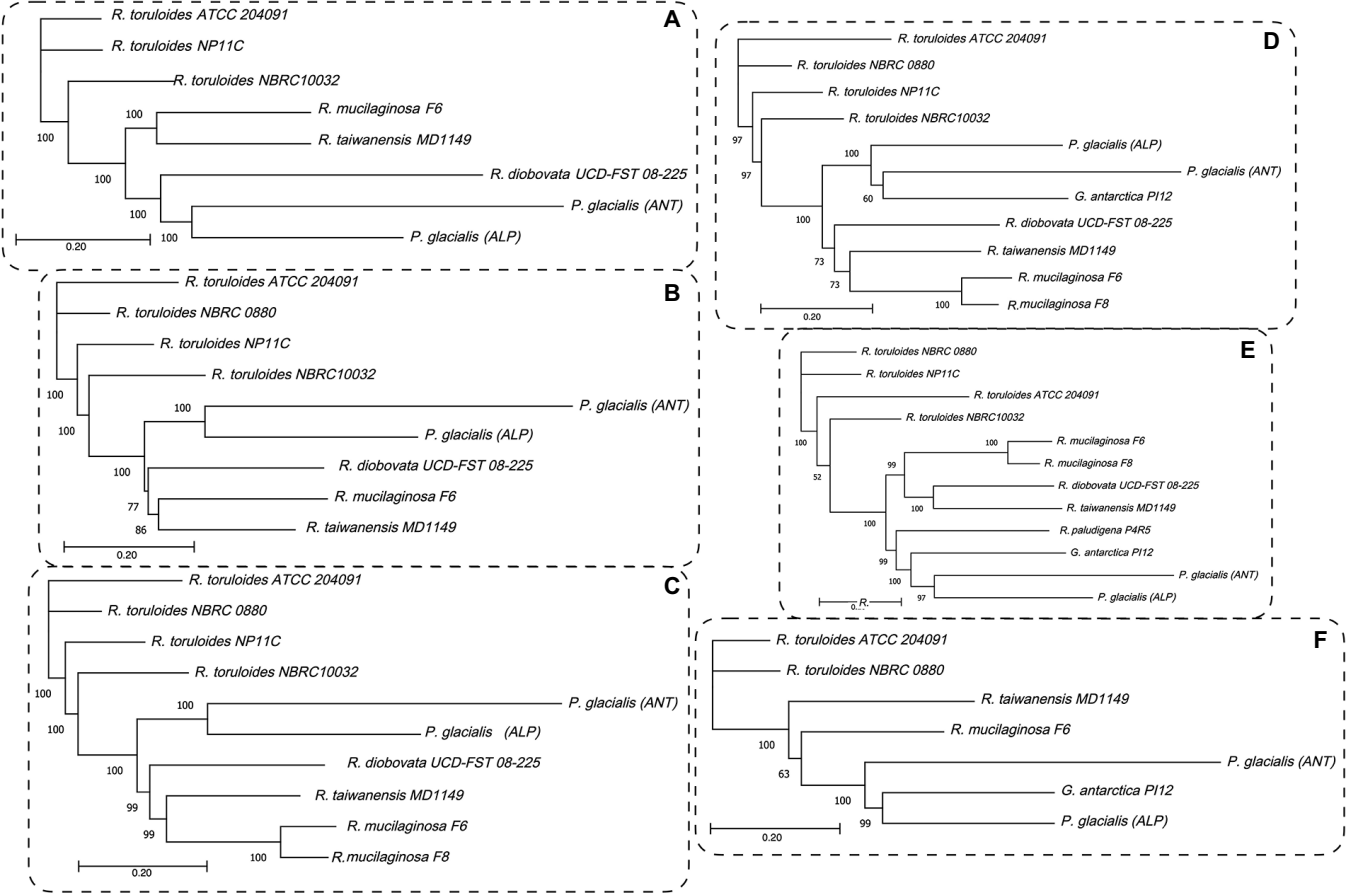
Maximum likelihood phylograms based on the concatenated CDSs. The coding sequences for proteins common to at least 8 yeasts, including both *P. glacialis* isolates, were concatenated and the evolutionary distances computed by Jukes-Cantor method. The phylogenetic trees obtained by neighbor-joining with 1,000 bootstrap replications and statistically supported bootstrap values (≥50%) are shown. **(A)**, 50 proteins common to 8 yeasts; **(B)**, 40 proteins common to 9 yeasts, **(C)**, 34 proteins common to 10 yeasts; **(D)**, 22 proteins common 11 yeasts; **(E)**, 17 proteins common to 12 yeasts. In **(F)**, was considered the proteins common at least for *P. glacialis* and *G. antarctica* (31 proteins, common to 7 yeasts). Abbreviation of yeasts as in Figure 6.

To highlight the differences between strains belonging to the same species but isolated from different geographical areas, the strain DBVPG 5880 of *P. glacialis* isolated from Italian Alps, was compared to an isolate from Antarctica, and to other members of the order Kriegeriales with available genomes at the NCBI database. The identified CDS encoding for proteins common to at least 8 yeasts (including both *P. glacialis* isolates) were considered for analysis at individual proteins and concatenated CDSs. In the trees generated from individual analysis of 65 proteins, both isolates of *P. glacialis* were generally closed related (Supplementary Figure S3). In some cases, alpine *P. glacialis* was closest to *G. antarctica* PI12, such as for proteins 2-isopropyl malate synthase, 3-hydroxybutyrate dehydrogenase, and acetyl-CoA carboxylase. Figure 6 summarized the results of trees classified for proteins common to 8–12 yeasts. The two strains of *P. glacialis* clustered in closed related positions in just 2 of the 6 trees obtained (Figures 6D,E); in the trees with higher number of analyzed proteins (Figures 6A–C,F) Antarctic *P. glacialis* was always positioned external to the entire group of strains, differently from Alpine *P. glacialis*. When the analysis was performed with concatenated CDS, both *P. glacialis* isolates clustered together and with *Rhodotorula* species (especially with *R. diobovata*, *R. taiwanensis*, and *R. mucilaginosa*) which vary in dependence of the number of CDS analyzed (Figures 7A–C), and with *G. antarctica* (Figures 7D,E). When the analysis was restricted to all CDS common exclusively to *P. glacialis* isolates and *G. antarctica*, these three yeasts clustered with *R. taiwanensis* MD1149, and *R. mucilaginosa* F6, with a close relation between alpine *P. glacialis* and *G. antarctica* PI12 (Figure 7F).

## Discussion

The four yeasts under study showed variable growth parameters (OTGs ranging from 10°C to 20°C). Interestingly, *N. antarctica* showed its max Gr at 4°C, but its max OTG (the highest A_600_ during the exponential phase) was found at 15°C. The long lag phase may explain the data observed at 4°C, in comparison with those obtained at 10°C and 15°C. The maximum A_600_ was reached by *G. gilvescens* and *P. glacialis*, which also showed the highest Gr and the ability to grow at 25°C, although the growth of *P. glacialis* was greatly reduced. All the strains were isolated from cold habitats of Italian Alps and Apennines and grow at 4°C, but only *G. martinii* and *N. antarctica* exhibited no growth at temperature equal or above 20°C. The Alpine *P. glacialis* strain herein studied differed from a previously studied Antarctic isolate of the same species exhibiting higher OTG and temperature range for growth (Baeza et al., 2021). This temperature range is narrower than that exhibited by isolates of the yeast-like fungus *Aureobasidium subglaciale*, which grew from 0°C to 30°C, and even tolerated incubation at 50°C for 2 h (Zajc et al., 2022). The sizes of draft genomes and GC contents ranged from 20.7 to 33.8 Mpb and 50.9% to 61.2%, respectively, in agreement with other yeasts isolated from cold habitats (Brejová et al., 2017; Coleine et al., 2019, 2020; Cunha et al., 2020; Baeza et al., 2021). Comparing the calculated protein flexibilities among yeasts, significant differences were found mainly for medium plus very flexible (M1 + 2, predicted by MEDUSA), which was higher for *G. martinii* than *G. gilvescens* and *N. antarctica*, and for *P. glacialis* than *G. gilvescens* and *N. antarctica*. Considering the growth parameters, the yeasts showing most significant differences in OTG were also discriminated according to their predicted protein flexibilities; similar clustering did not occur for Gr. Overall, a correlation between protein flexibilities calculated as M1 + 2 and yeast growth parameters was directly proportional when considering OTG and variable for Gr. Similarly, in previous genomic and transcriptomic studies of eight Antarctic yeasts, a correlation was observed between calculated protein flexibilities and OTGs, not Gr (Baeza et al., 2021, 2022). Interestingly, the correlation between OTG and calculated protein flexibilities was negative in Antarctic yeasts, while it was positive in the yeasts isolated from the Alps and Apennines. It could be an interesting point concerning potential differences in adaptation strategies in yeasts colonizing different cold environments, thus suggesting the need of a more in-depth study of cold-adapted yeasts at the genomic and biochemical levels.

A considerable number of putative genes involved in the regulation of response to different kinds of stress were found, including, in addition to cold, general, oxidative, and osmotic stress, thus highlighting the putative adaptation of yeasts to the challenging environmental conditions occurring in Alpine ecosystems. Some genes associated with response to cold were found, among them: dihydrolipoamide dehydrogenase, enoyl-[acyl-carrier protein] reductase II, glycogen synthase, omega-6 fatty acid, desaturase/acyl-lipid omega-6 desaturase, stearoyl-CoA desaturase (Delta-9 desaturase), ATP-dependent RNA helicase DeaD, elongation of very-long-chain fatty acids protein 6, glycogen debranching enzyme, and very-long-chain acyl-CoA dehydrogenase, involved in different cellular functions such as cell membrane adaptation, compatible solute accumulation, regulation of protein folding, and changes in RNA structure. Similar genes patterns were also found in other microorganism (including yeasts), as a common reply to cold stress (Aguilera et al., 2007; Inouye and Phadtare, 2014; Zhang and Gross, 2021). Among the more represented pathways MAPK signaling, spliceosome, ribosome, and autophagy were observed, especially in *P. glacialis* and *G. martinii*. In a genomic and comparative transcriptomic study of a cold-adapted oleaginous yeast *R. kratochvilovae*, cultivated at 15°C and 30°C, the significantly downregulated genes were primarily related to metabolic and cellular processes and cellular organelles, whereas the MAPK signaling pathway was among overrepresented pathways (Guo et al., 2021). A recent study of Chen et al. (2022) demonstrated that the overexpression and knockout of the MAP-Kinase HOG1 played an important role in cold adaptation of *R. kratochvilovae*, mainly by promoting the biosynthesis of polyunsaturated fatty acids, such as linoleic acid and linolenic acid, and glycerol (Chen et al., 2022). Autophagy denotes a variety of mechanisms for the metabolization *via* vacuole of aged and damaged proteins, lipids, and membrane-bound organelles and has also been associated play a critical role in aging (Tyler and Johnson, 2018). The role of autophagy in response to abiotic stress, including cold, has been studied mainly in plants and fishes (Chen et al., 2021; Ren et al., 2021). A considerable number of genes classified in the autophagy pathway were found in Antarctic yeasts, especially in *Candida sake* (Baeza et al., 2021). Among complete modules according to putative genes in the four studied yeasts, the pathways for polyamine biosynthesis and citrate cycle (TCA cycle Krebs cycle) were found. TCA-cycle metabolites, lactic acid, and polyamines were accumulated in the Antarctic yeast *Mrakia blollopis* after a cold shock exposure (Tsuji, 2016). Polyamines are aliphatic hydrocarbon chains containing one or more amine groups. They have recognized functions in cell growth, development, and cellular protection against stresses such as oxidative, osmotic and temperature (Valdés-Santiago and Ruiz-Herrera, 2013; Gevrekci, 2017; Murray Stewart et al., 2018). Putative antifreeze glycopeptide AFGP polyprotein and antifreeze protein were identified in *N. antarctica* and *P. glacialis*. Similarly, a putative gene encoding for ice-binding protein was found in the whole genome of the endemic Antarctic ascomycetous fungus *Antarctomyces pellizariae* (Batista et al., 2020). The antifreeze or Ice Binding Proteins (IBPs) can bind to the ice surface and be incorporated into the ice crystal matrix controlling the ice shape and inhibiting re-crystallization, potentially decreasing cell injury (DeVries, 1986; Bouvet and Ben, 2003; Garner and Harding, 2010). IBPs also stimulate microbial biofilm formation on ice and the formation of channels through the ice, helping interchange of nutrients and gasses (Davies et al., 2002; Bar Dolev et al., 2016; Cheung et al., 2017). Most studies revealed the production of IBPs by fish, bacteria, and plants, and in a more limited way by fungi. The IBP AFP1, produced by *G. antarctica*, was successfully expressed in *E. coli* (Hashim et al., 2013). Antifreeze proteins from the Antarctic yeast *Goffeauzyma gastrica*, maintained, after partial purification, a good antifreeze property after several freeze–thaw cycles (Villarreal et al., 2018).

The redundancy in putative CDS encoding for the same protein or proteins families was one additional aspect that can be putatively considered as an adaptation strategy of yeasts to their extreme environments. Gene duplication has been proposed as a mechanism of genomic adaptation (as a genetic robustness) to a changing environmental conditions, including transport of nutrients, and protection to biotic and abiotic stress (Levasseur and Pontarotti, 2011; Kondrashov, 2012). P-loop containing nucleoside triphosphate hydrolase (P-loop NTPase), Alpha/Beta hydrolase *(α/β hydrolase), Armadillo repeat-containing proteins (ARMCs)*, and the major facilitator superfamily protein (MFS) were found in the studied genomes among the most redundant ones. P-loop NTPase is a superfamily of enzymes crucial for almost all aspects of life, and some members contribute to stress responses in plants (Leipe et al., 2002; Cheung et al., 2013, 2016). *α/β Hydrolase* is a superfamily of hydrolytic enzymes that share a common fold having widely catalytic functions and phylogenetic origin (Ollis et al., 1992). Some *α/β hydrolases* have been involved in salt and cold tolerance in plants and microorganisms, including yeasts (Brody et al., 2001; Takahashi et al., 2006; Liu et al., 2014; Wu et al., 2020). *ARMCs* form a large family of enzymes primarily characterized in multicellular organisms (e.g., in plants) and exhibiting very versatile and fundamental functions, including response to stress, such as osmotic (Kojima et al., 2013; Sharma and Pandey, 2015; Huang et al., 2021). MFS is a ubiquitous transporter superfamily whose members have a broad spectrum of substrates, such as inorganic and amino acids, nucleosides, lipids, and short peptides (Yan, 2015); some members of MFS are involved in resistance to oxidative stress and fungicides in fungal pathogen *Alternaria alternata* (Chen et al., 2017). An increase of MFS copy numbers led to an increase of the gene redundancy in Antarctic yeast *Naganishia vishniacii* (Nizovoy et al., 2021) and *Mrakia psychrophila* isolated from permafrost on the Qinghai-Tibet Plateau (Su et al., 2016) and suggested that it could be considered a further adaptation strategy contributing to the accumulation of nutrients under cold imposed conditions.

Gene clusters for the biosynthesis of secondary metabolites were predicted: terpene clusters were found in all four sequenced yeasts, NRSP in *G. gilvescens*, *G. martinii*, and *P. glacialis*, and T3PKS in *G. gilvescens* and *N. antarctica*. NRPS was mainly found in bacteria and fungi, especially Ascomycota, which assemble numerous peptides having functions in primary metabolism, cellular development and morphology, and stress responses such as oxidative in *Aspergillus fumigatus* (Stack et al., 2007; Süssmuth and Mainz, 2017; Sayari et al., 2019). Terpenoids are structurally the most diverse groups of natural compounds, widely distributed in nature, that play important physiological and metabolic functions, mainly antioxidant activities in microorganisms (Avalos and Carmen Limón, 2015; Jaeger and Cuny, 2016). Type III PKS has widely described in plants and bacteria and recently in Antarctic yeasts (Baeza et al., 2021), and produces a wide array of compounds such as chalcones, pyrones, acridones, phloroglucinols, and stilbenes, with several physiological roles such as pigmentation, salinity and dehydration resistance, stress adaptation, and cell wall remodeling (Yu et al., 2012; Parvez et al., 2018; Navarro-Muñoz and Collemare, 2019).

A class of genetic elements with increasing relevancy are putative functional small ORFs (SmORFs) of 10–100 codons (which millions can be found in eukaryotic genomes) generally considered non-coding sequences because of their short length that impair bioinformatic analysis (Albuquerque et al., 2015; Couso and Patraquim, 2017). In *G. antarctica* SmORFs represented 4% of annotated ORFs, validated as expressed transcripts *via* cross-referencing against available transcriptome data (Mat-Sharani et al., 2015). High numbers of SmORFs were found in all four Alpine yeasts studied, especially in *P. glacialis*, of which 19% were at 5′UTR. The SmORFs showed to be “exclusive” for each yeast as no identical SmORFs at nucleotide sequence were found and resulted infimum when translated sequences were compared; only 0.1%–0.5% of them showed similarity to SmORFs of other organisms, mainly from *H. sapiens* and mouse.

The function mainly described for SmORFs, especially those at 5′UTR, is related to the regulation of the expression of the main ORF by mechanisms that include the alteration of ribosome capacity to initiate, terminate and reinitiate translation (Wang and Rothnagel, 2004; Gunišová and Valášek, 2014; Renz et al., 2020). Although some examples of microproteins working independently have been found, such as repressors in *S. cerevisiae*, *Neurospora crassa* and *H. sapiens* (Renz et al., 2020), their appropriate functional study is still complicated. Anyway, it is possible to postulate a role for SmORFs as an additional adaptation strategy of yeasts to cold environments.

The results obtained by the comparison of the genomes showed that Alpine *P. glacialis* herein studied exhibited some differences when compared to another strain of the same species, but isolated from Antarctica (Baeza et al., 2021). The draft genome of Alpine *P. glacialis* was 33.8 Mbp with 61.2% GC (87% completeness), whiles Antarctic *P. glacialis* was 15.9 Mb with 50.4% GC (completeness 71%). Two terpene and one NRPS biosynthetic cluster were predicted in Alpine *P. glacialis* and three NRPS in Antarctic *P. glacialis*. The ratio sum of CDS / total of different putative proteins was 2.1 for Alpine strain and 1.8 for the Antarctic one. When comparing the top 20 redundant putative proteins, just 9 of them exhibited a coincidence between both yeasts: alcohol dehydrogenase, α/β hydrolase, general substrate transporter, MFS, NADP-dependent oxidoreductase domain-containing, P-loop NTPase, and pyridoxal phosphate-dependent transferase. In the comparisons by multigene phylogeny computed with protein sequences individually and concatenated CDS of both strains, including other yeasts of the order Kriegeriales, the two *P. glacialis* did not always cluster together and, in some trees, they showed a close relation with G. *antarctica* PI12.

## Conclusion

The findings obtained from the genomic approach used in this study to analyze the cold adaptation of the four studied yeasts are consistent with those reported for other yeasts isolated from cold habitats regarding genomic structure. Furthermore, the high numbers of putative genes encoding for cold stress response and gene clusters encoding for the synthesis of secondary metabolites suggest the ability of such yeasts to successfully survive and adapt their physiology to these harsh ecological conditions. The different correlation between the OTG and calculated protein flexibilities found in yeasts isolated from the Italian Alps and Apennines and those from Antarctica suggest the existence of a certain degree of variability in adaptation strategies of yeasts thriving in different cold regions.

As expected, Alpine and Antarctic *P. glacialis* isolates were close in the multigene phylogenetic analysis, including other members of the order Kriegeriales. Interestingly, both strains were clustered close to *G. antarctica* PI12 suggesting more similitude among yeast from cold environments.

On the other hand, as already described for Antarctic yeasts, a considerable number of SmORFs and genetic redundancy were found. These two aspects are less studied and could be proposed to play roles in adaptation to cold, which are attractive to include in future comparative studies of adaptative yeast’ strategies from different cold environments worldwide.

## Data availability statement

The datasets presented in this study can be found in online repositories. The names of the repository/repositories and accession number(s) can be found at: NCBI BioProject, accession no: PRJNA859078.

## Author contributions

BT and PB carried out DNA purifications and determination of yeast growth parameters. MB performed bioinformatic and statistical analyses. MB, BT, and PB contributed to design of the experiments, discussion of the results, and manuscript writing. All authors have read and agreed to the published version of the manuscript.

## Funding

This research was funded by Agencia Nacional de Investigación y Desarrollo, grant number Fondecyt 1180233.

## Conflict of interest

The authors declare that the research was conducted in the absence of any commercial or financial relationships that could be construed as a potential conflict of interest.

## Publisher’s note

All claims expressed in this article are solely those of the authors and do not necessarily represent those of their affiliated organizations, or those of the publisher, the editors and the reviewers. Any product that may be evaluated in this article, or claim that may be made by its manufacturer, is not guaranteed or endorsed by the publisher.

## Footnotes

^1^ https://www.genome.jp/kaas-bin/kaas_main?mode

^2^ https://www.cuilab.cn/smorfunction/download

^3^ http://bigdata.ibp.ac.cn/SmProt/download.htm

## Supplementary material

Supplementary FIGURE S1 Prediction of function for genes in secondary metabolite clusters. The phylogenetic analysis included the best 10 Blastp hits for each sequence obtained from the NCBI database. The evolutionary history was inferred by using the Maximum Likelihood method and JTT matrix-based model. The percentage of trees in which the associated taxa clustered together is displayed next to the branches.

Supplementary FIGURE S2 Phylogenetic analysis for translated SmORFs. The analyses were performed with SmORFs common to three **(A)**, and all yeast **(B)** studied, including the reference Blastp hits SmORFs from Mouse and Literature mining classification. The evolutionary history was inferred by using the Maximum Likelihood method and JTT matrix-based model. The percentage of trees in which the associated taxa clustered together is displayed next to the branches.

Supplementary FIGURE S3 Phylogenetic analysis for individual proteins used in multigene phylogeny analysis. The evolutionary history was inferred by using the Maximum Likelihood method and JTT matrix-based model. The percentage of trees in which the associated taxa clustered together is displayed next to the branches.
