# Supplementary figures and images for: A genomic approach to analyze the cold adaptation of yeasts isolated from Italian Alps
Source: Front Microbiol. 2022 Nov 8;13:1026102. doi: 10.3389/fmicb.2022.1026102 (PMC9679224; doi:10.3389/fmicb.2022.1026102)

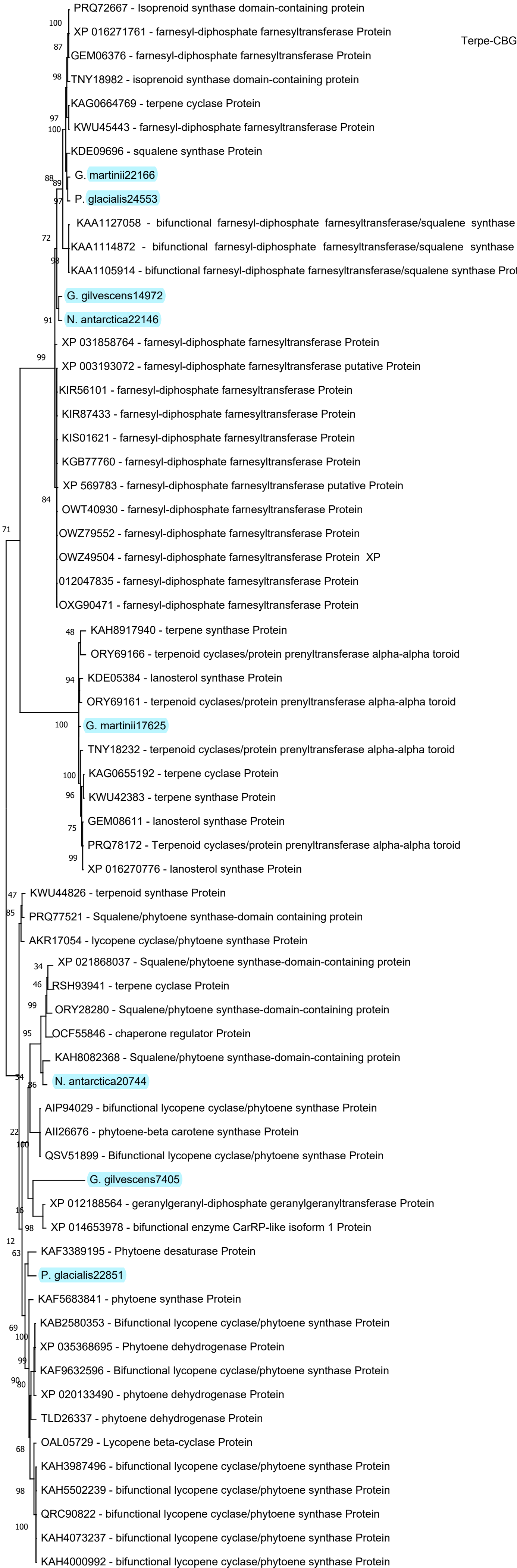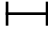

1.00

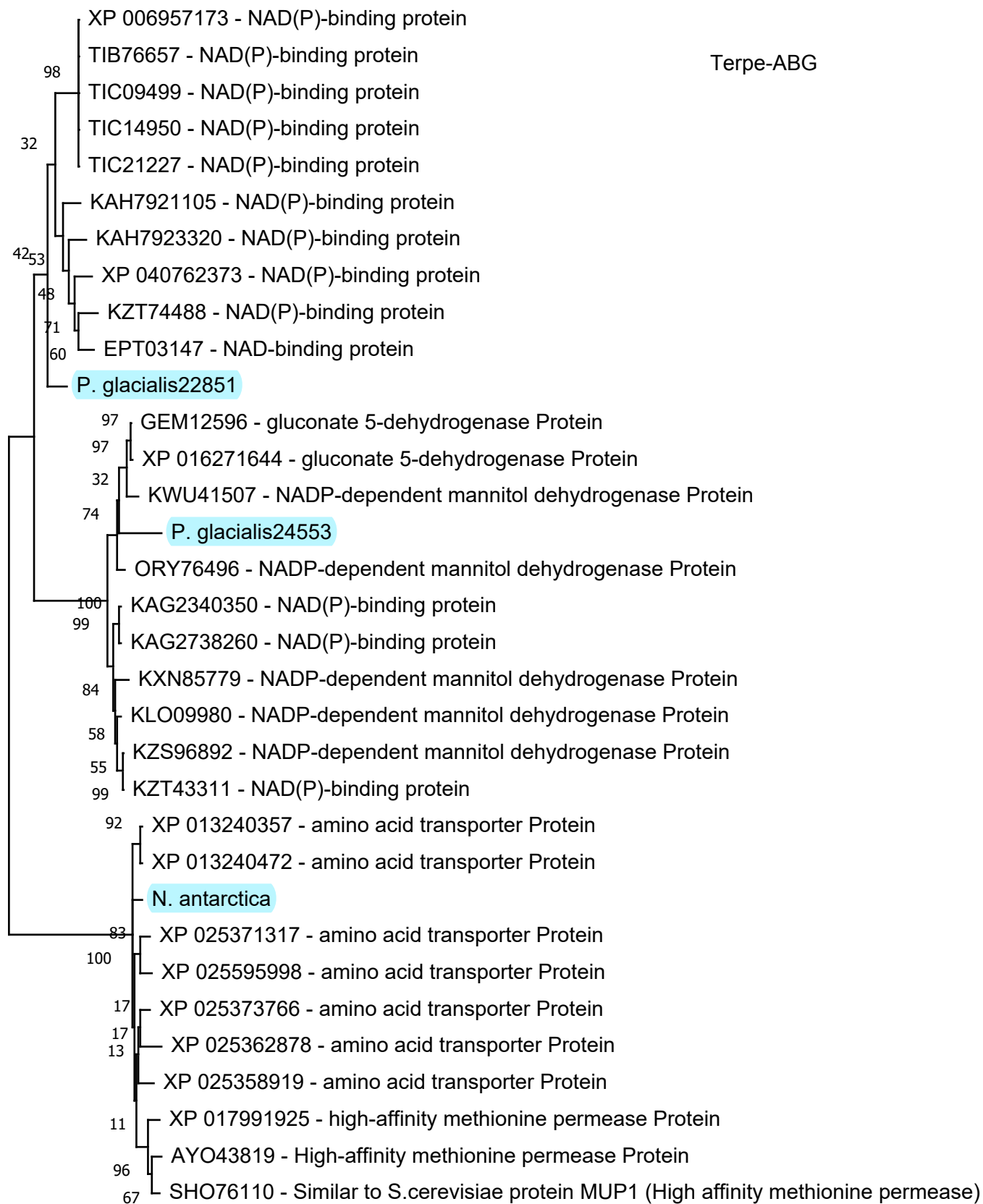

H

0.50

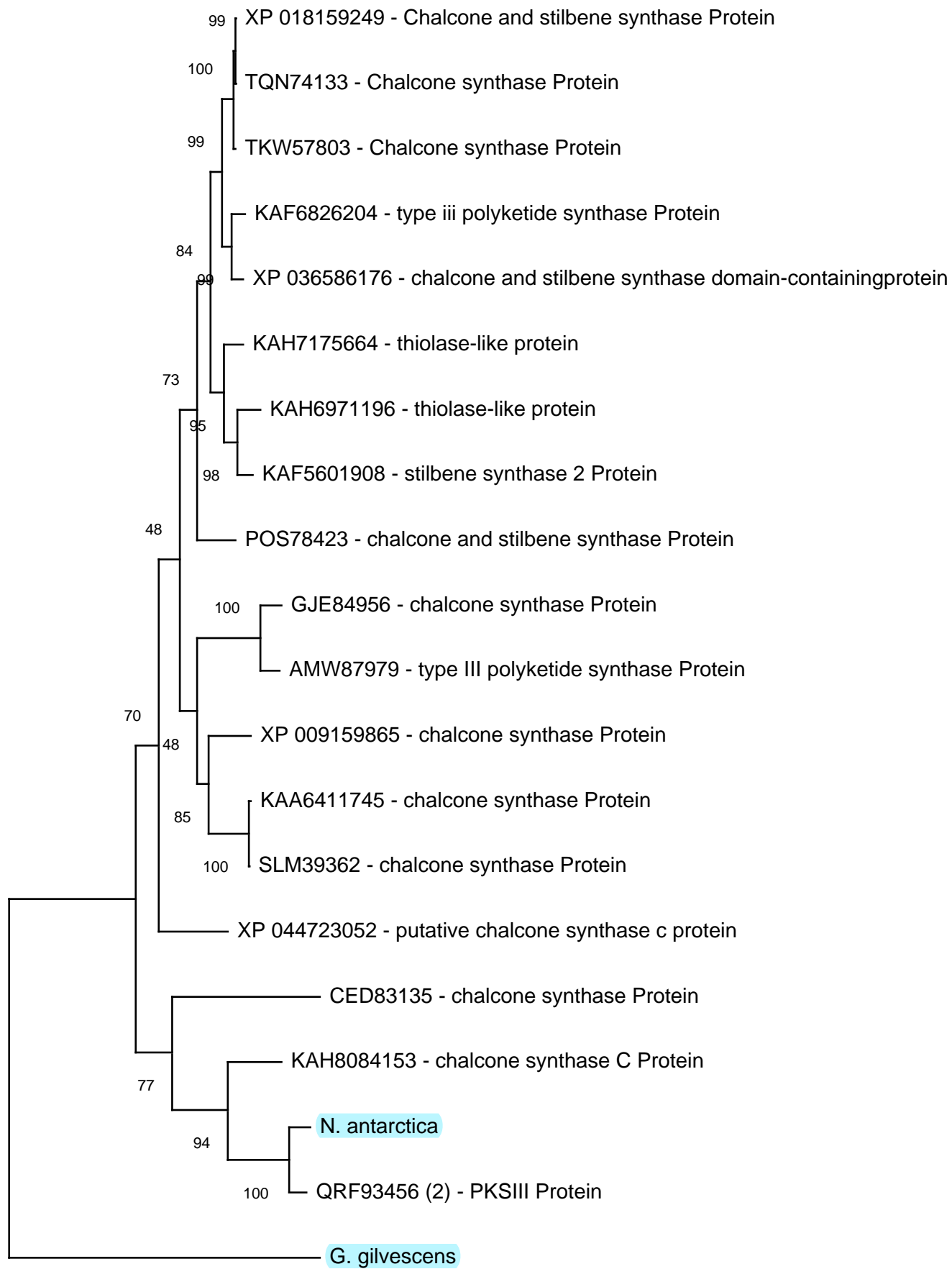

|

0.50

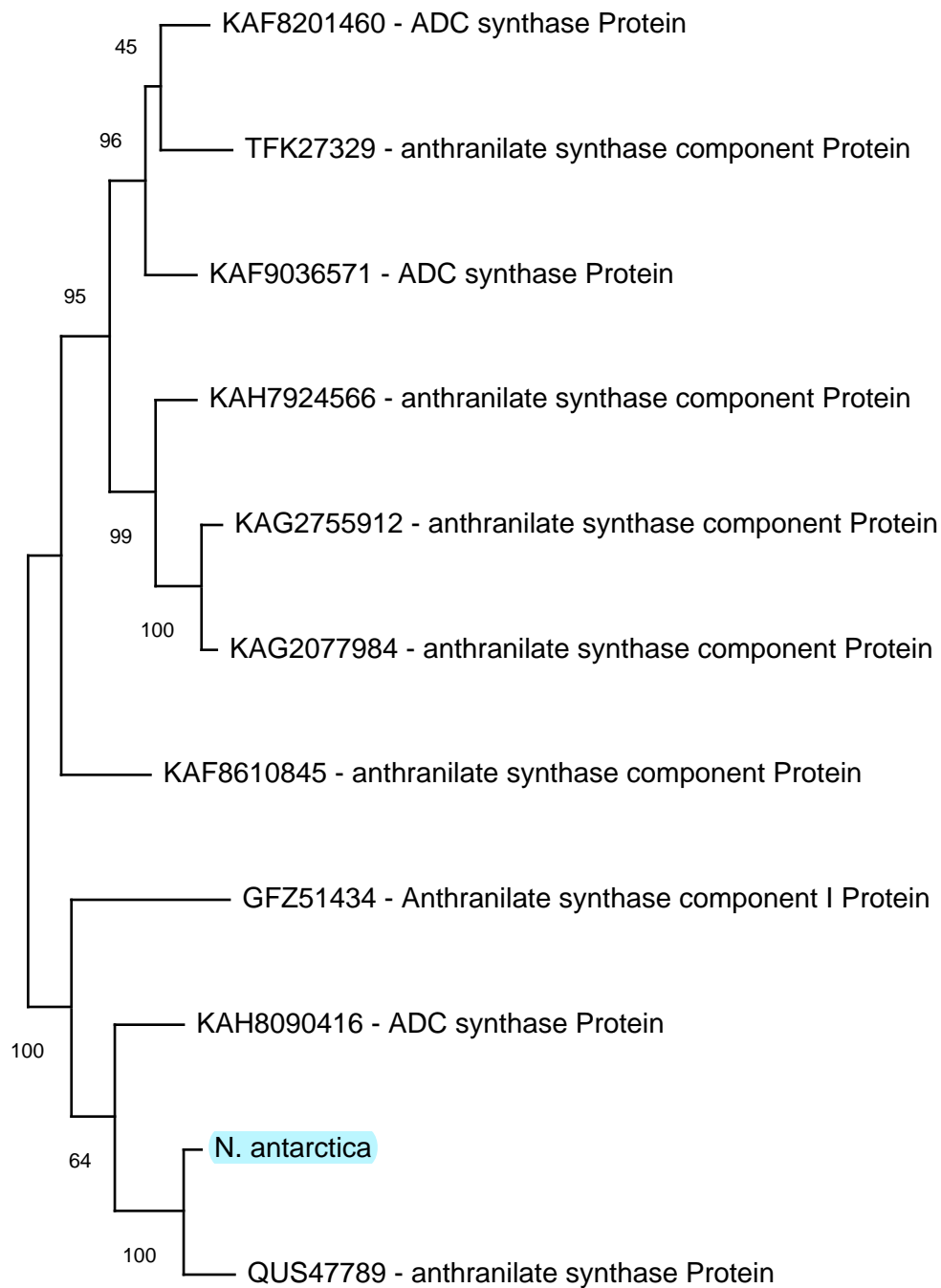

H

0.050

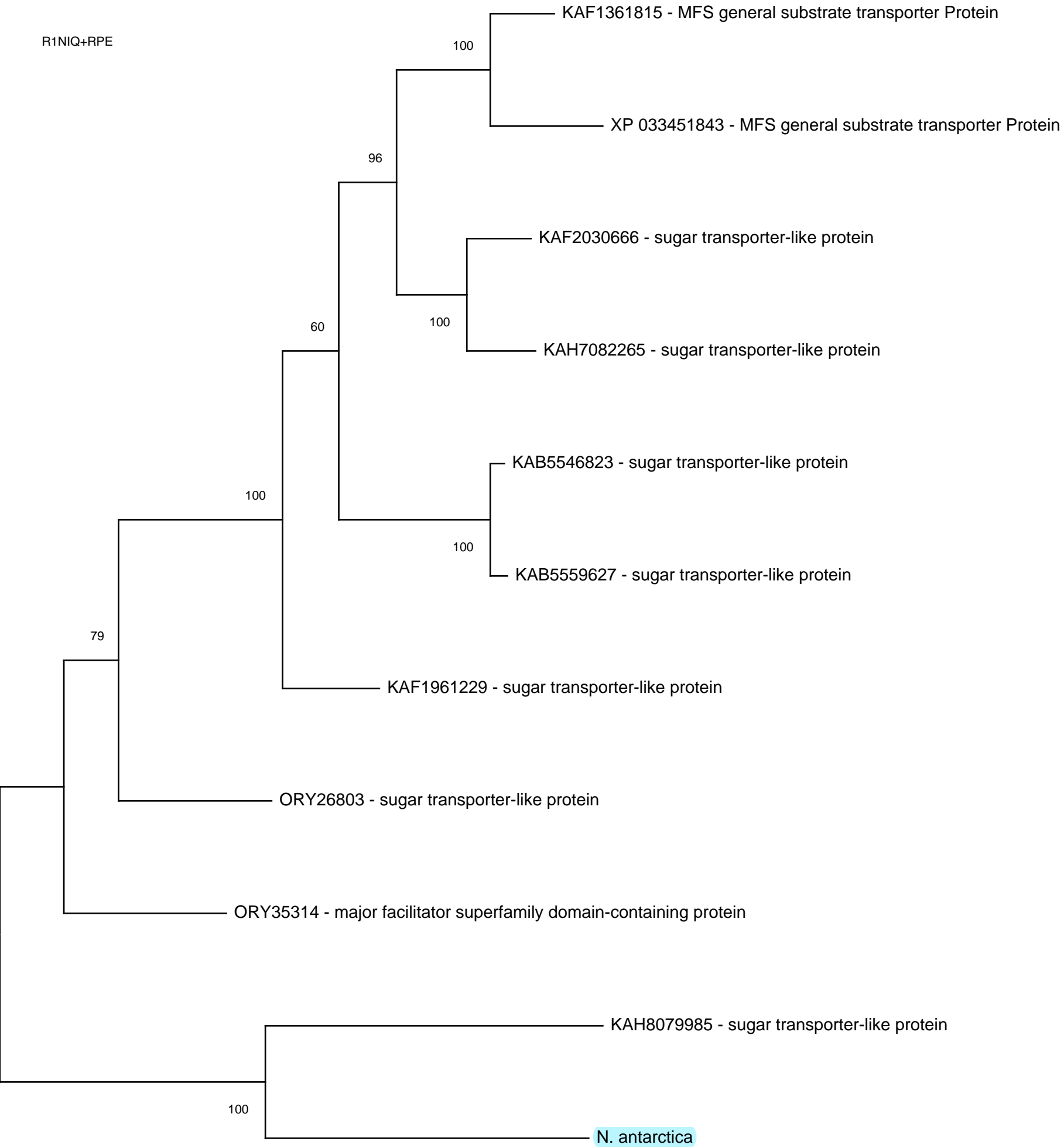

0.10

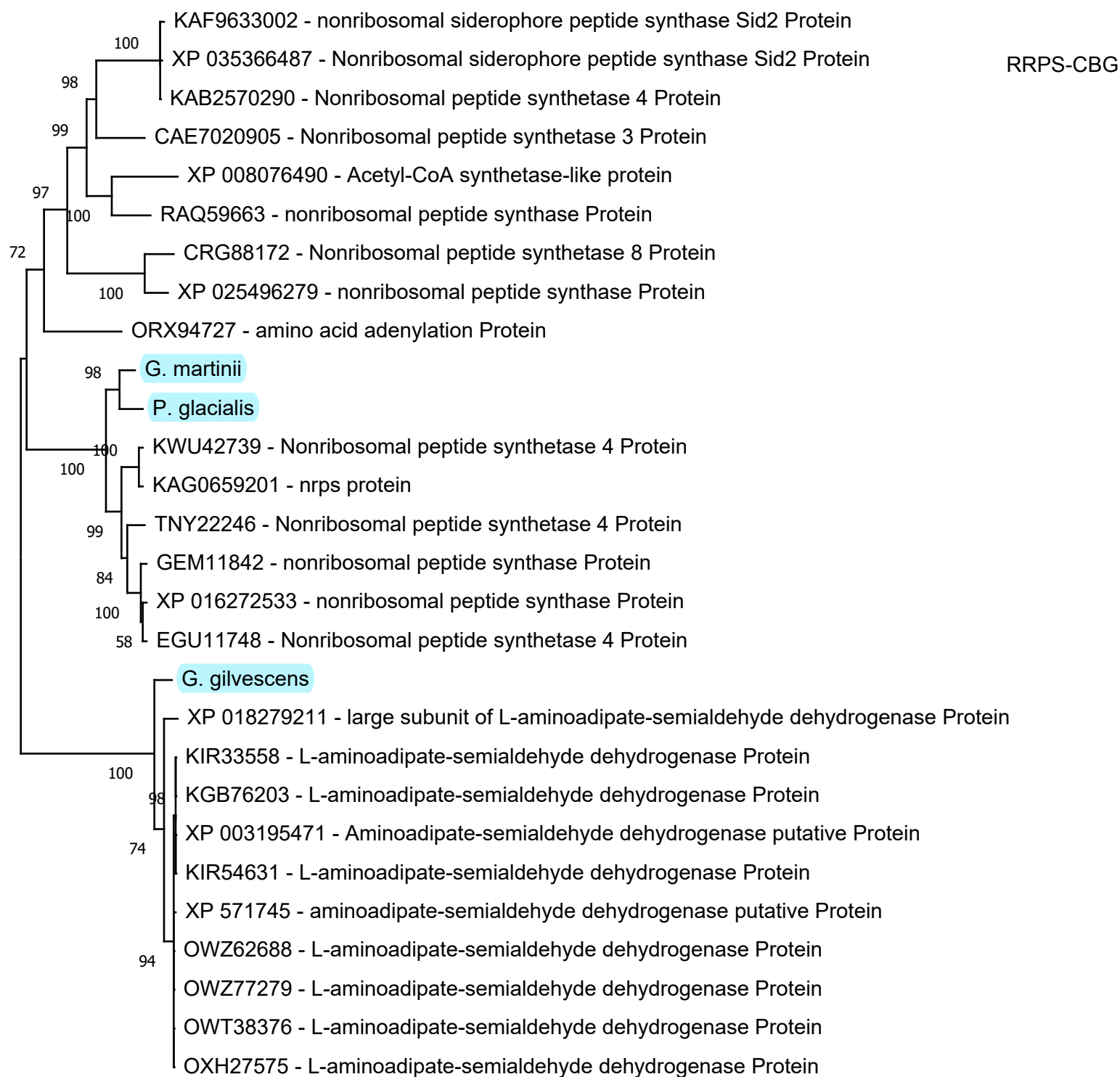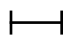

0,50

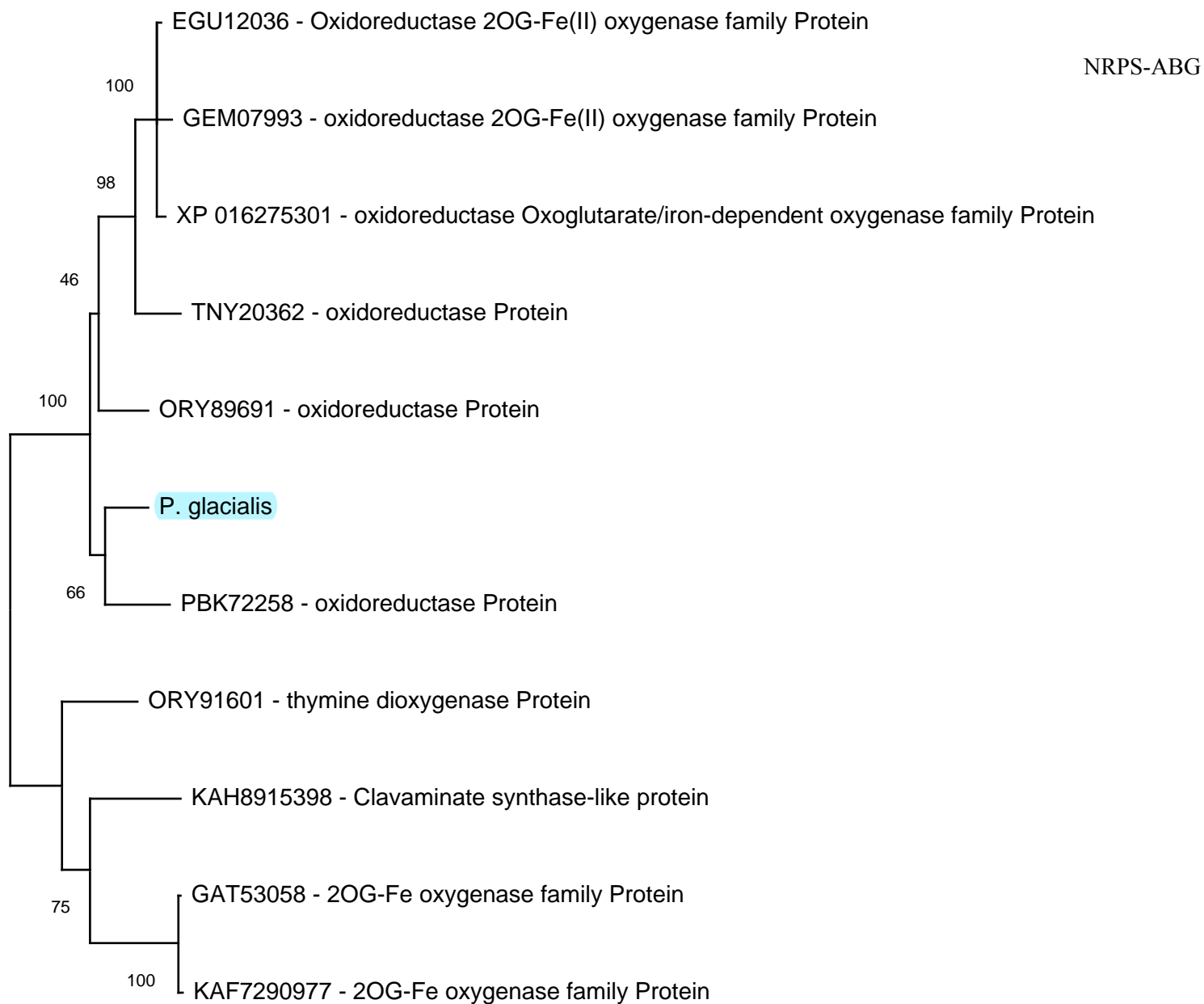

H

0.10

Supplement: Supplementary FIGURE S1 — Prediction of function for genes in secondary metabolite clusters. The phylogenetic analysis included the best 10 Blastp hits for each sequence obtained from the NCBI database. The evolutionary history was inferred by using the Maximum Likelihood method and JTT matrix-based model. The percentage of trees in which the associated taxa clustered together is displayed next to the branches. [file Data_Sheet_1.PDF]

A

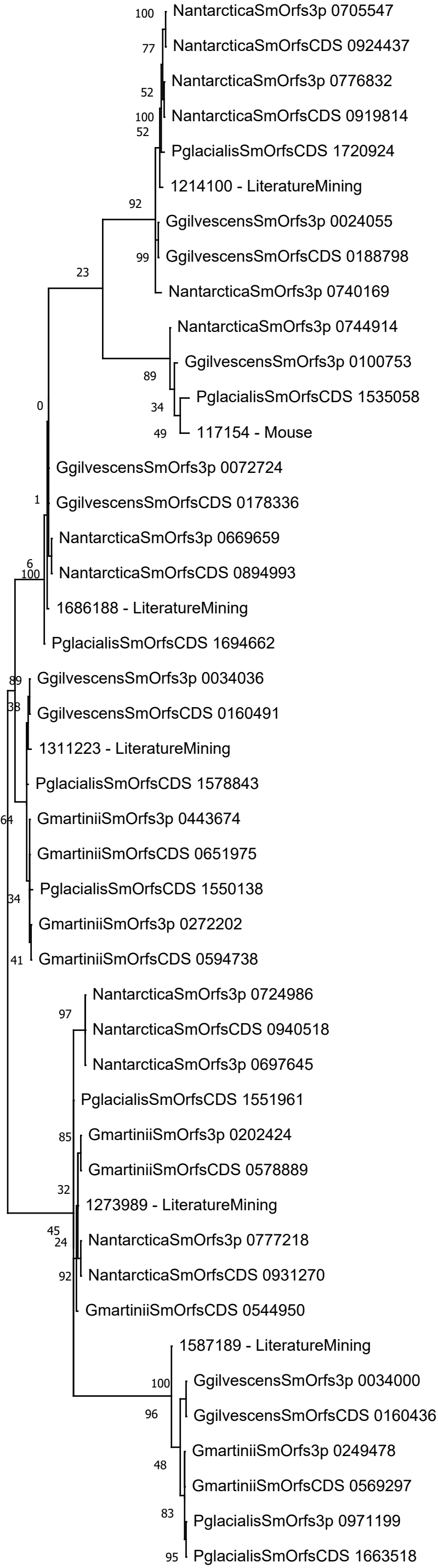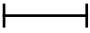

2.00

B

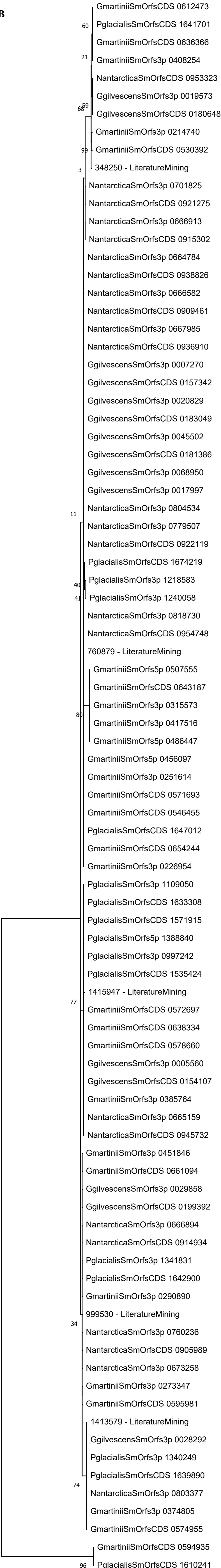

10.00

Supplement: Supplementary FIGURE S2 — Phylogenetic analysis for translated SmORFs. The analyses were performed with SmORFs common to three (A), and all yeast (B) studied, including the reference Blastp hits SmORFs from Mouse and Literature mining classification. The evolutionary history was inferred by using the Maximum Likelihood method and JTT matrix-based model. The percentage of trees in which the associated taxa clustered together is displayed next to the branches. [file Data_Sheet_2.PDF]

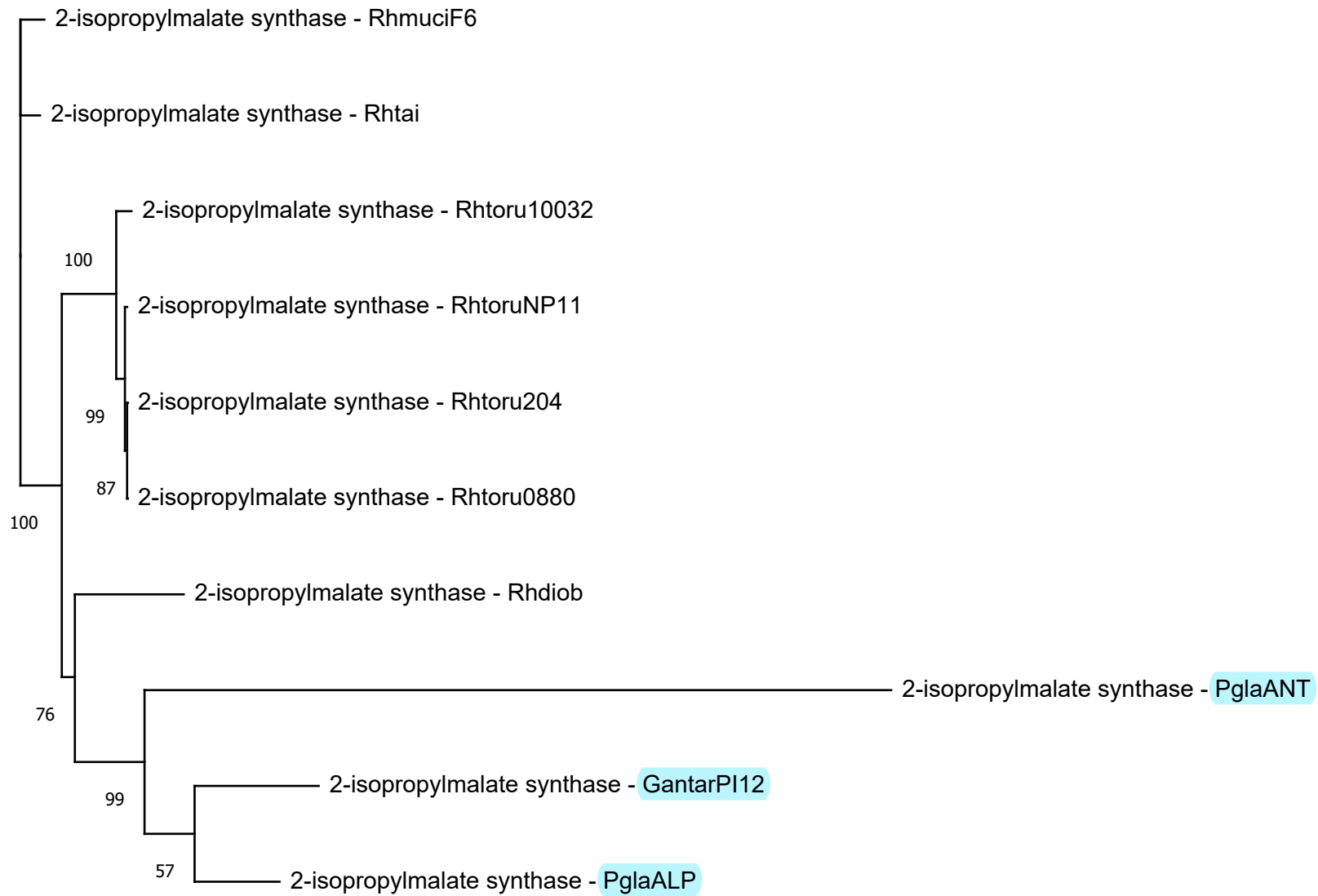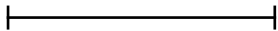

0.20

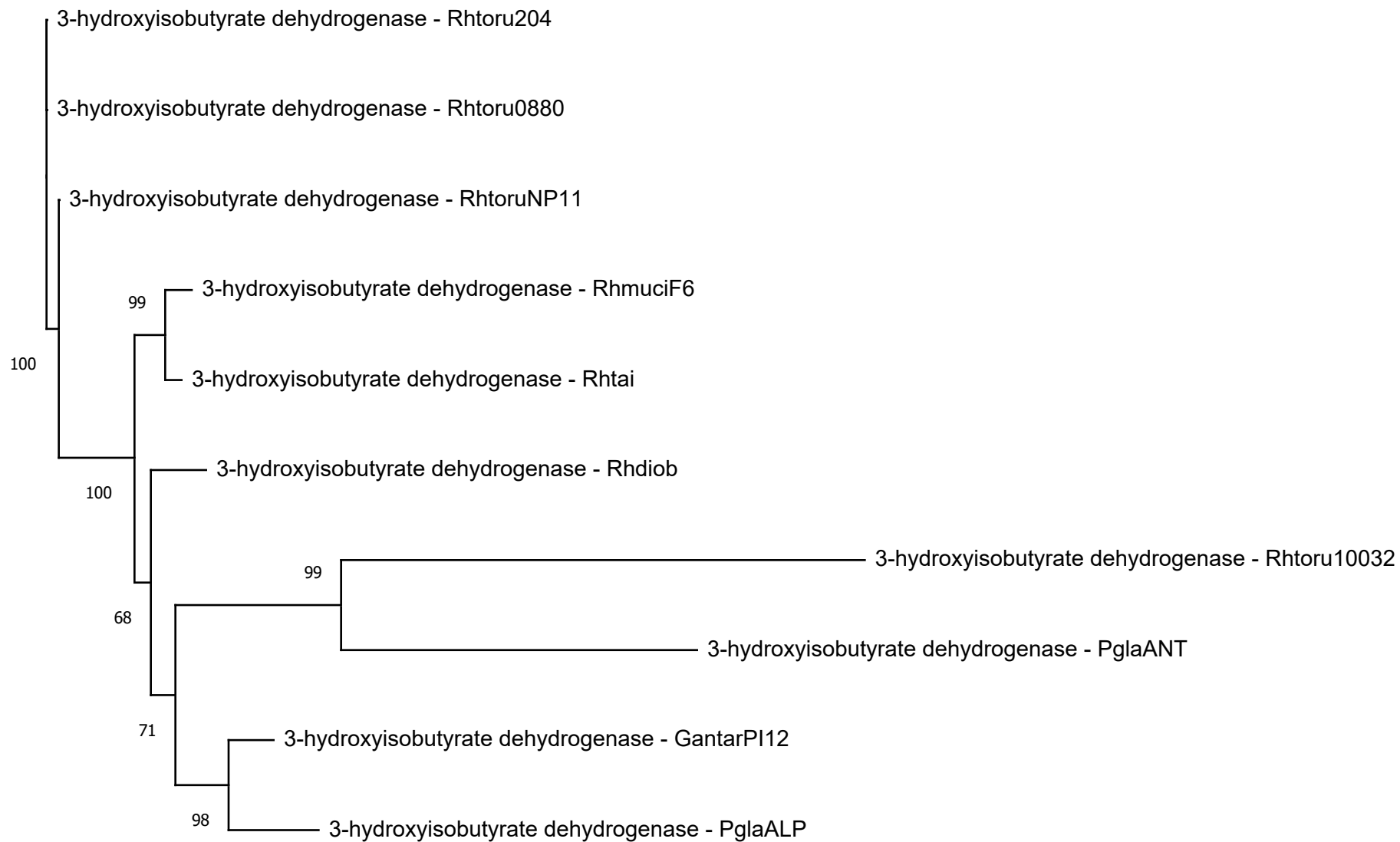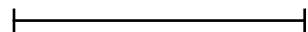

0.50

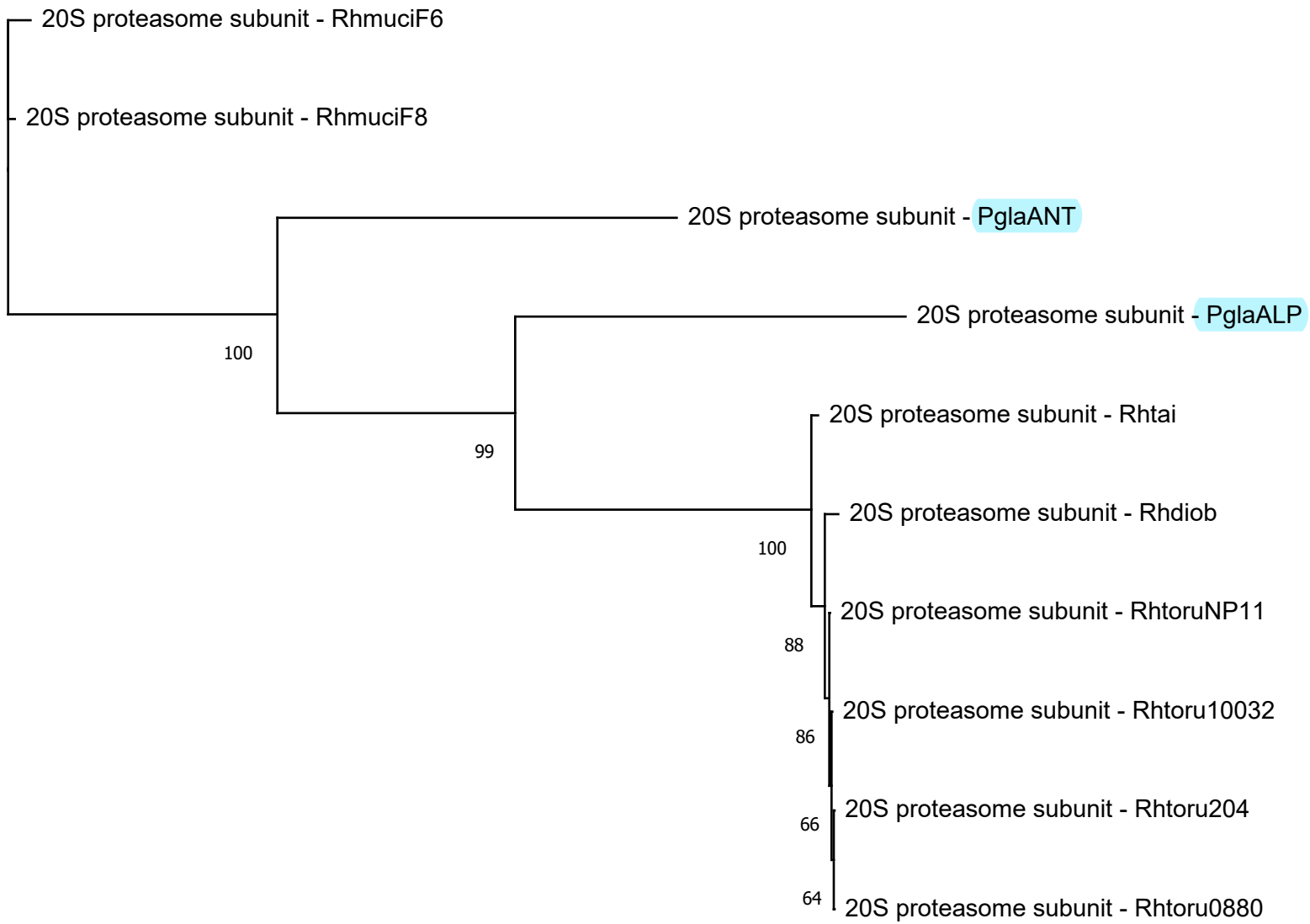

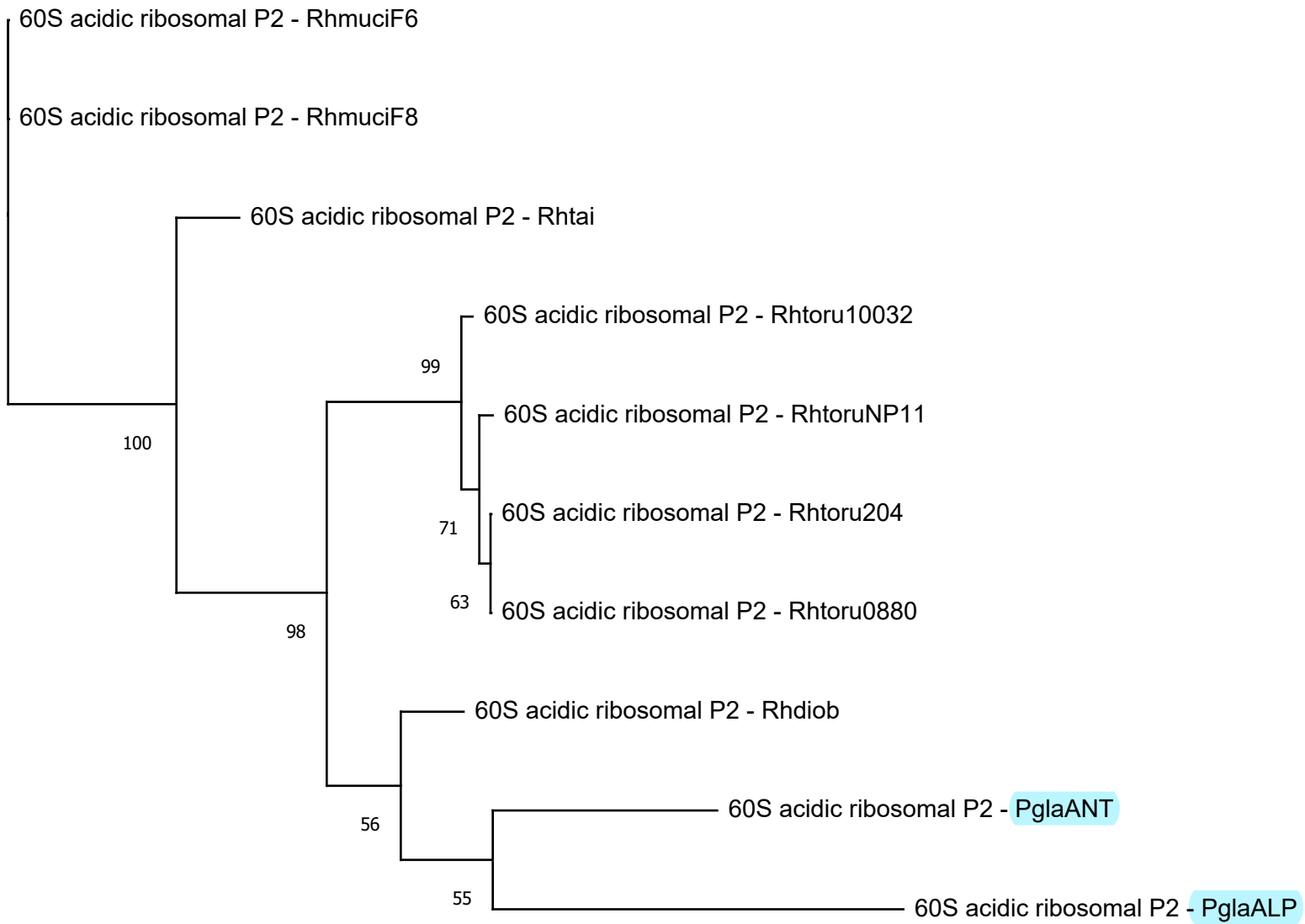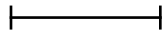

0.10

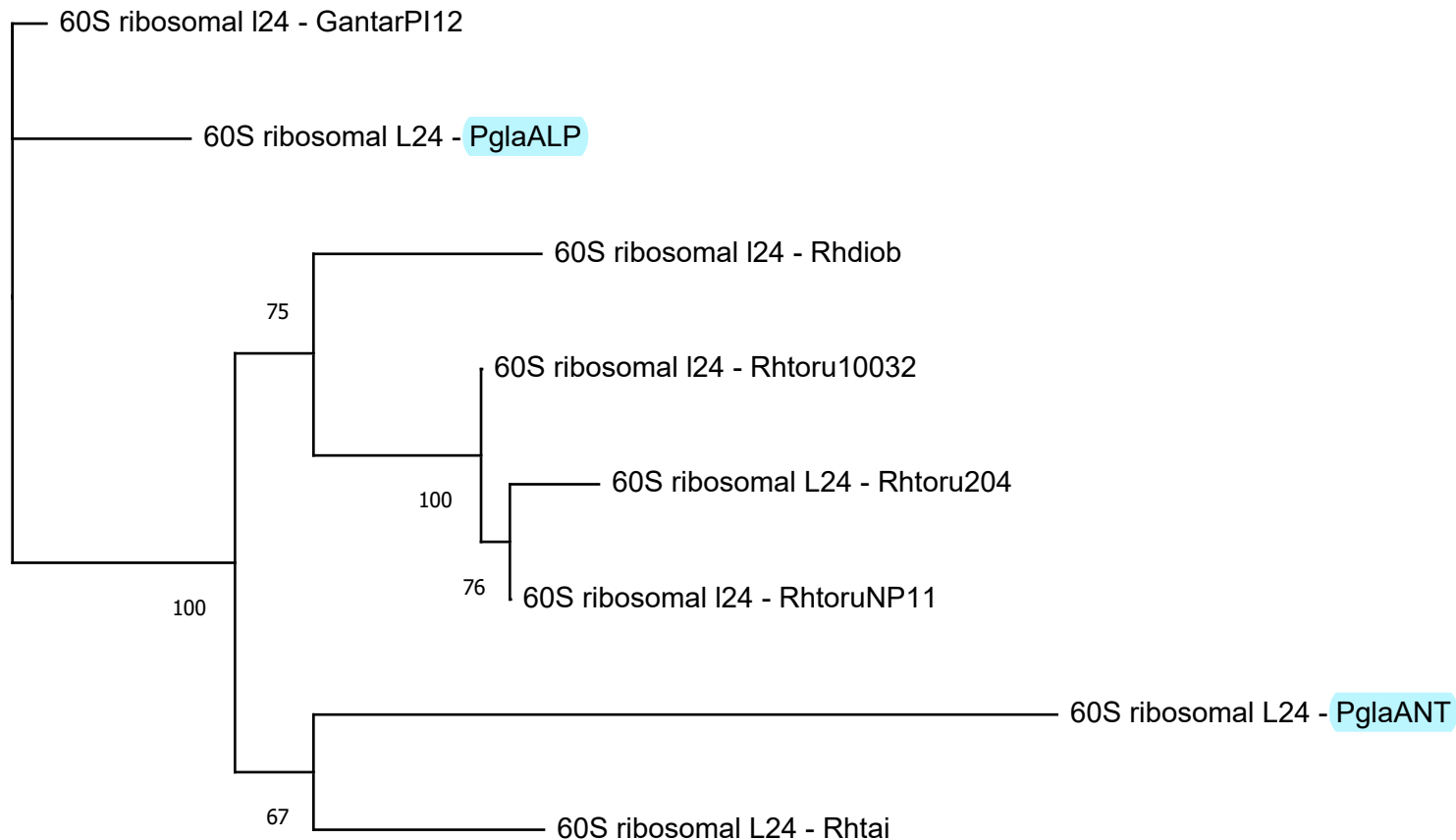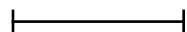

0.10

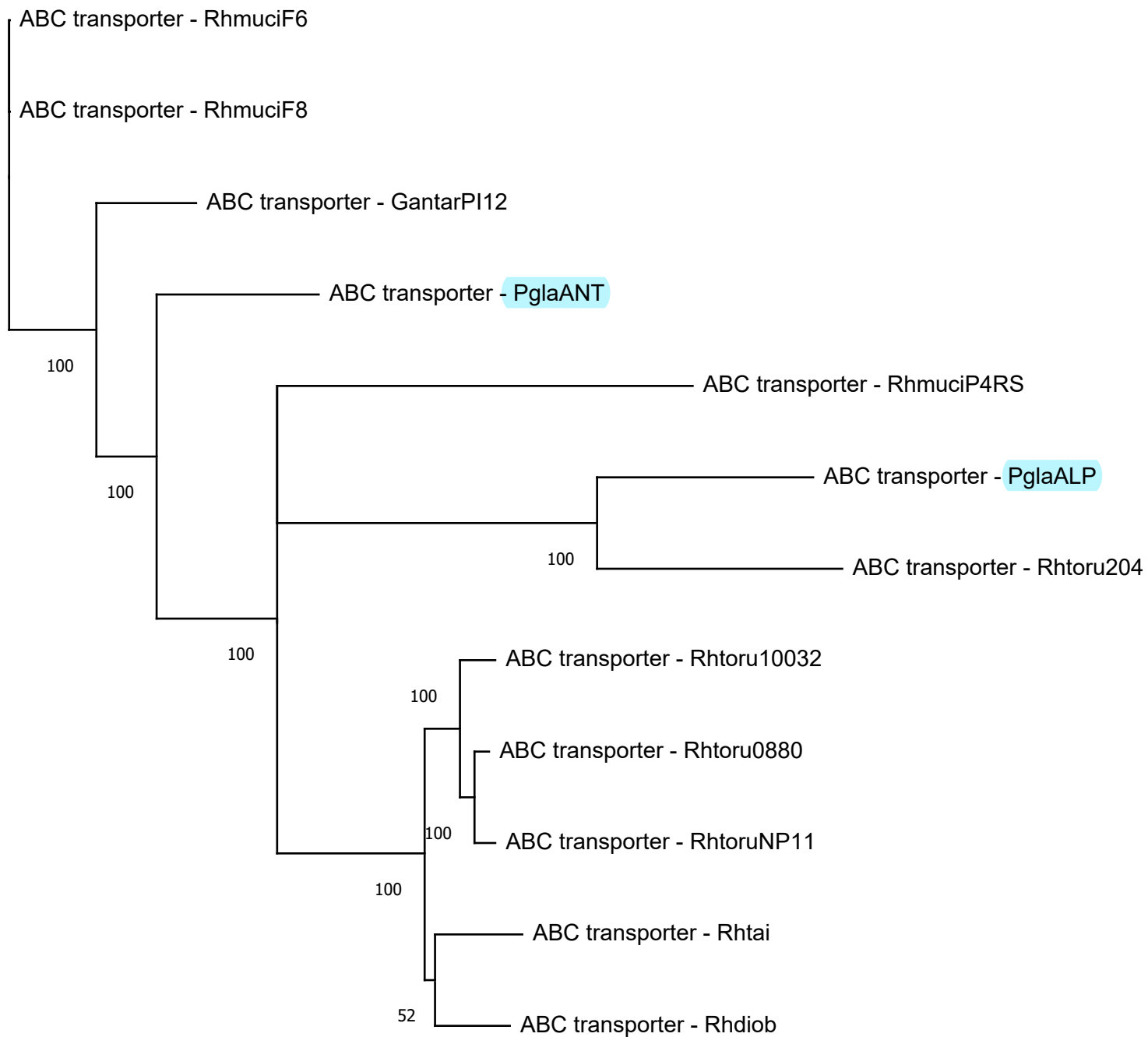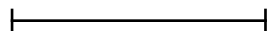

0.50

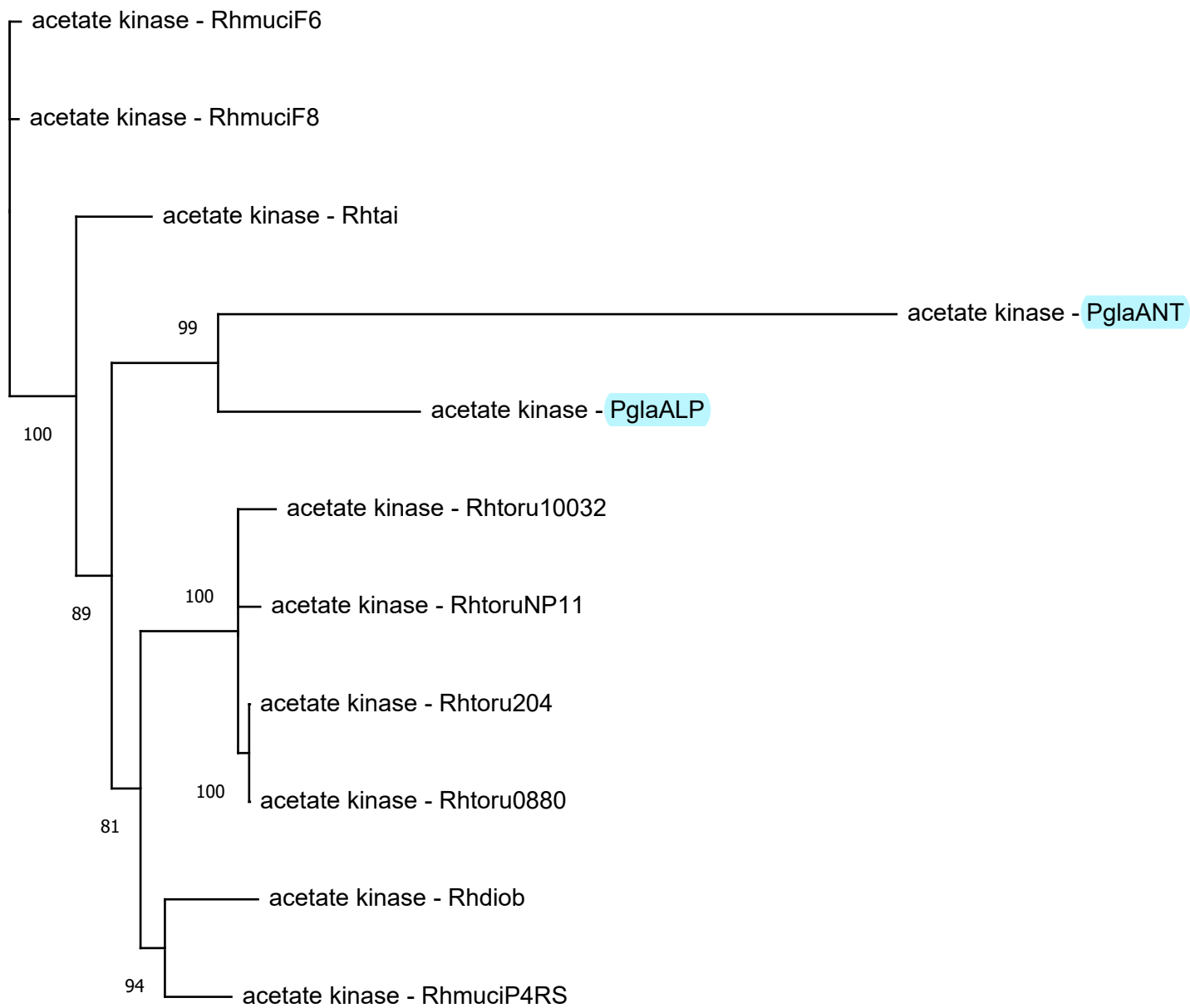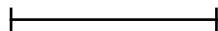

0.20

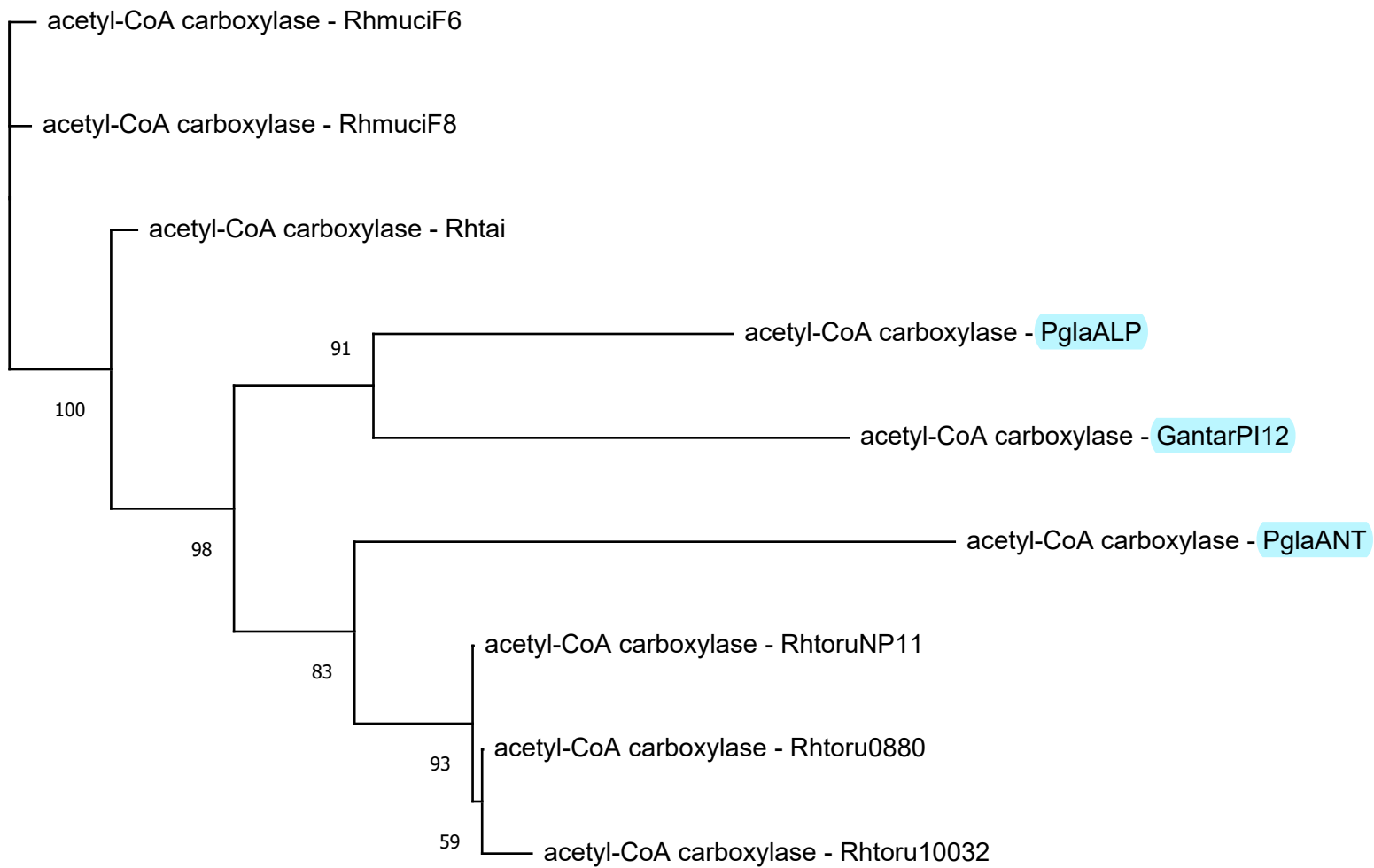

0.050

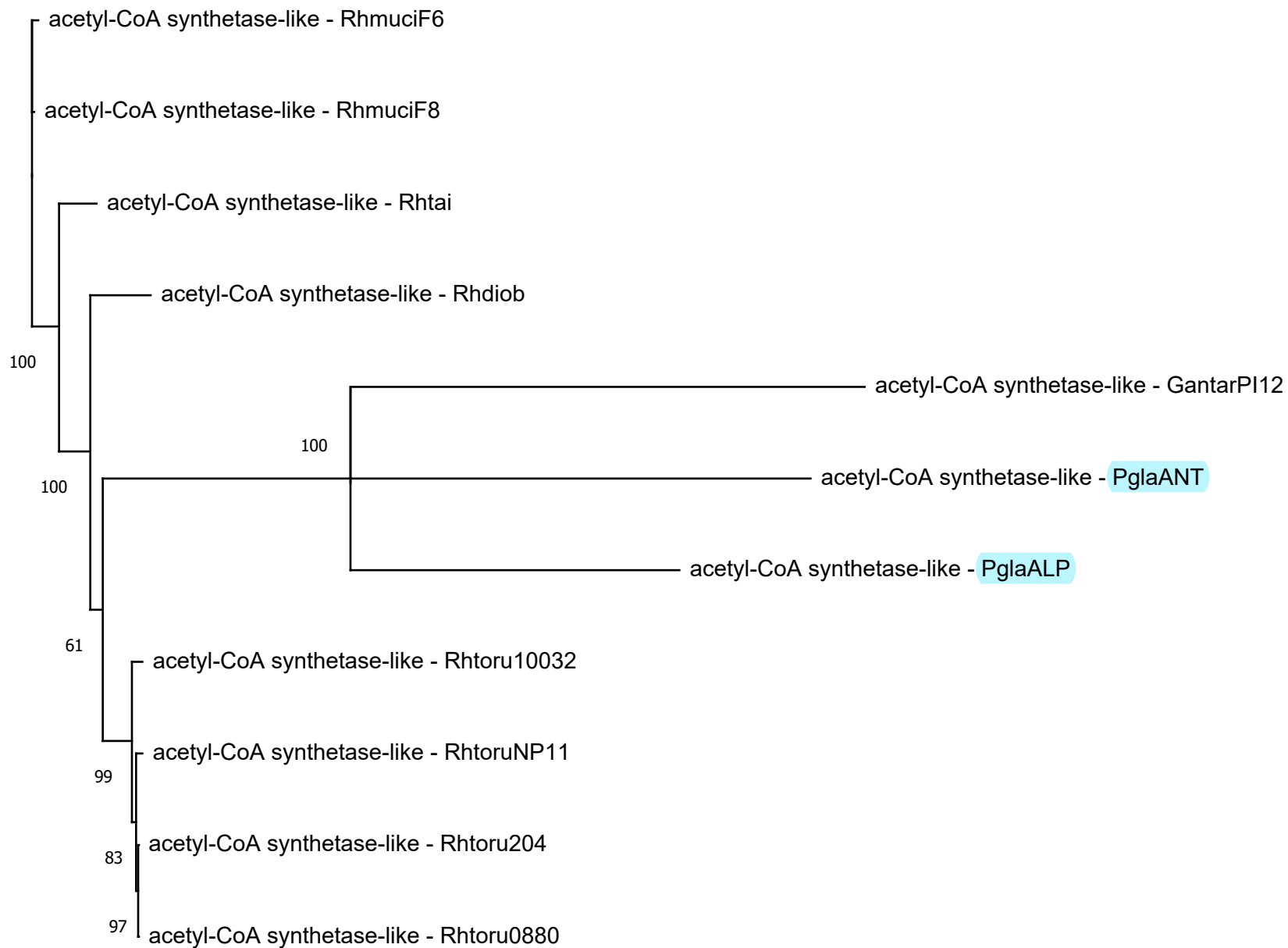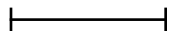

0.20

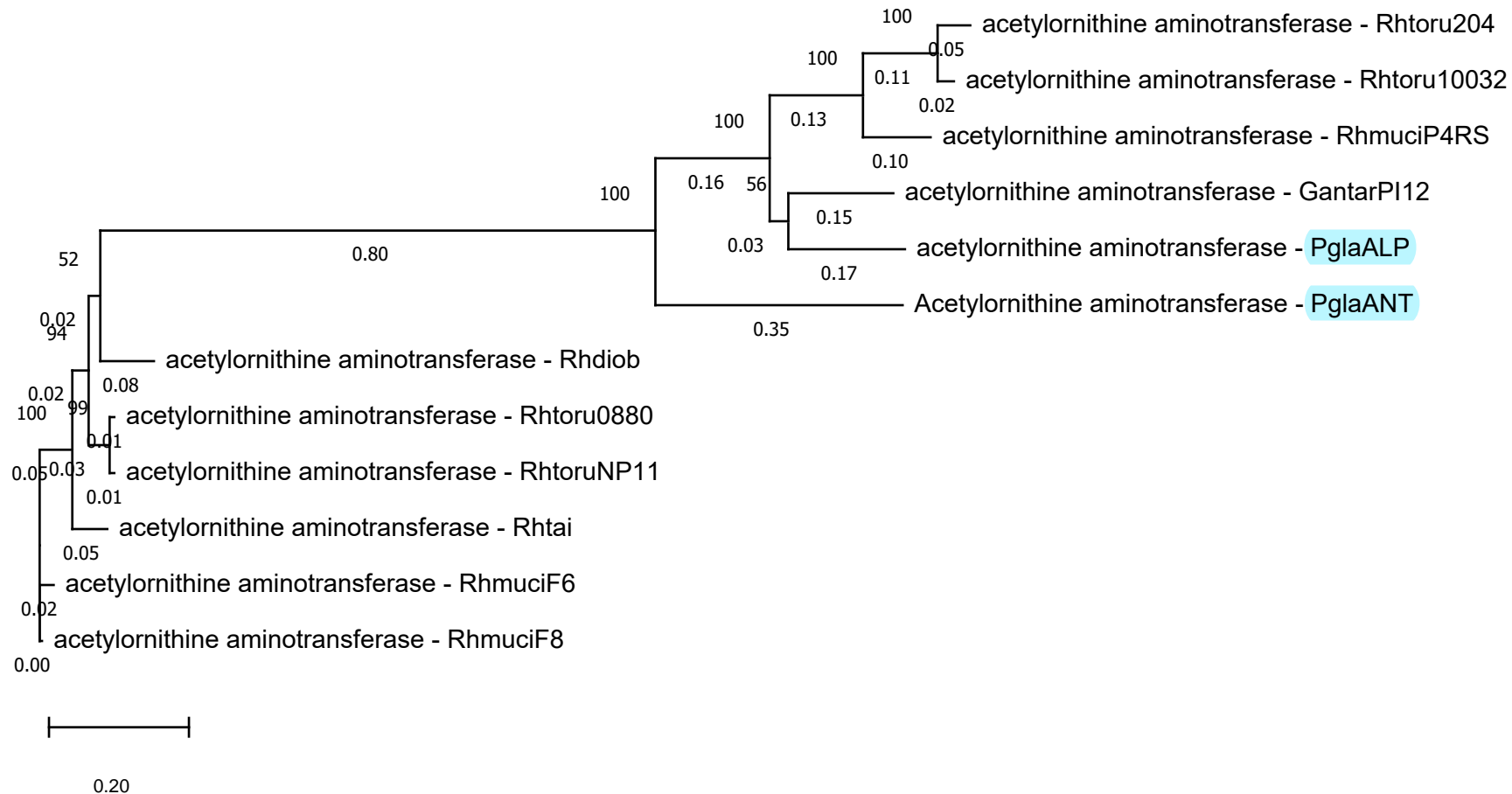

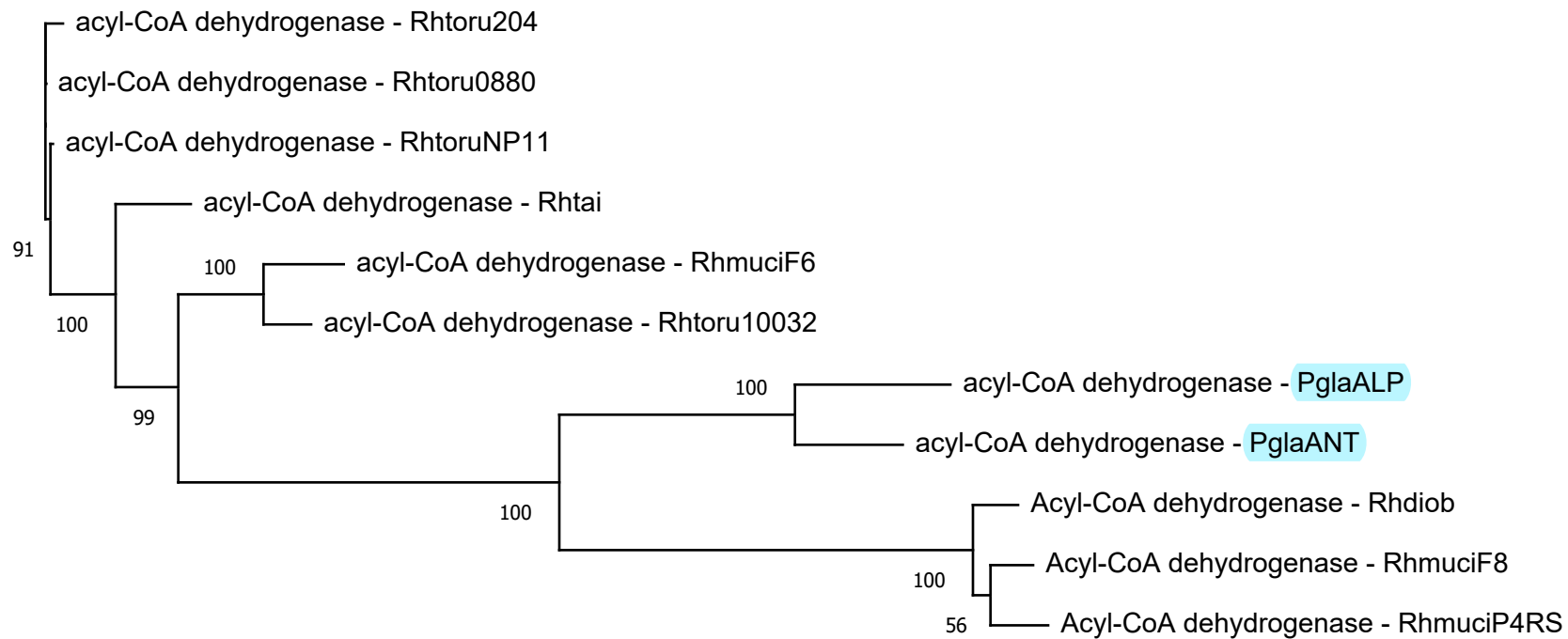

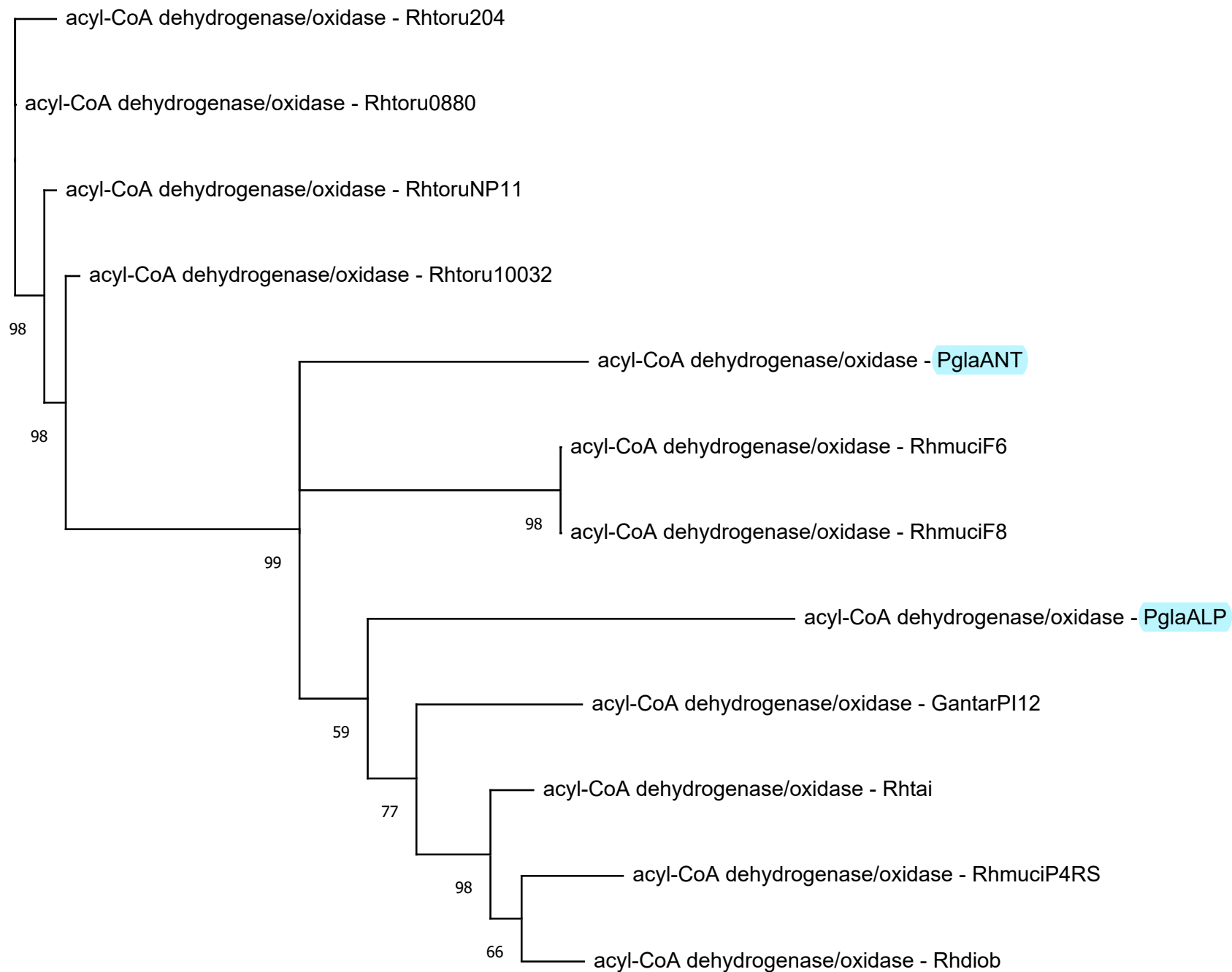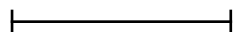

0.50

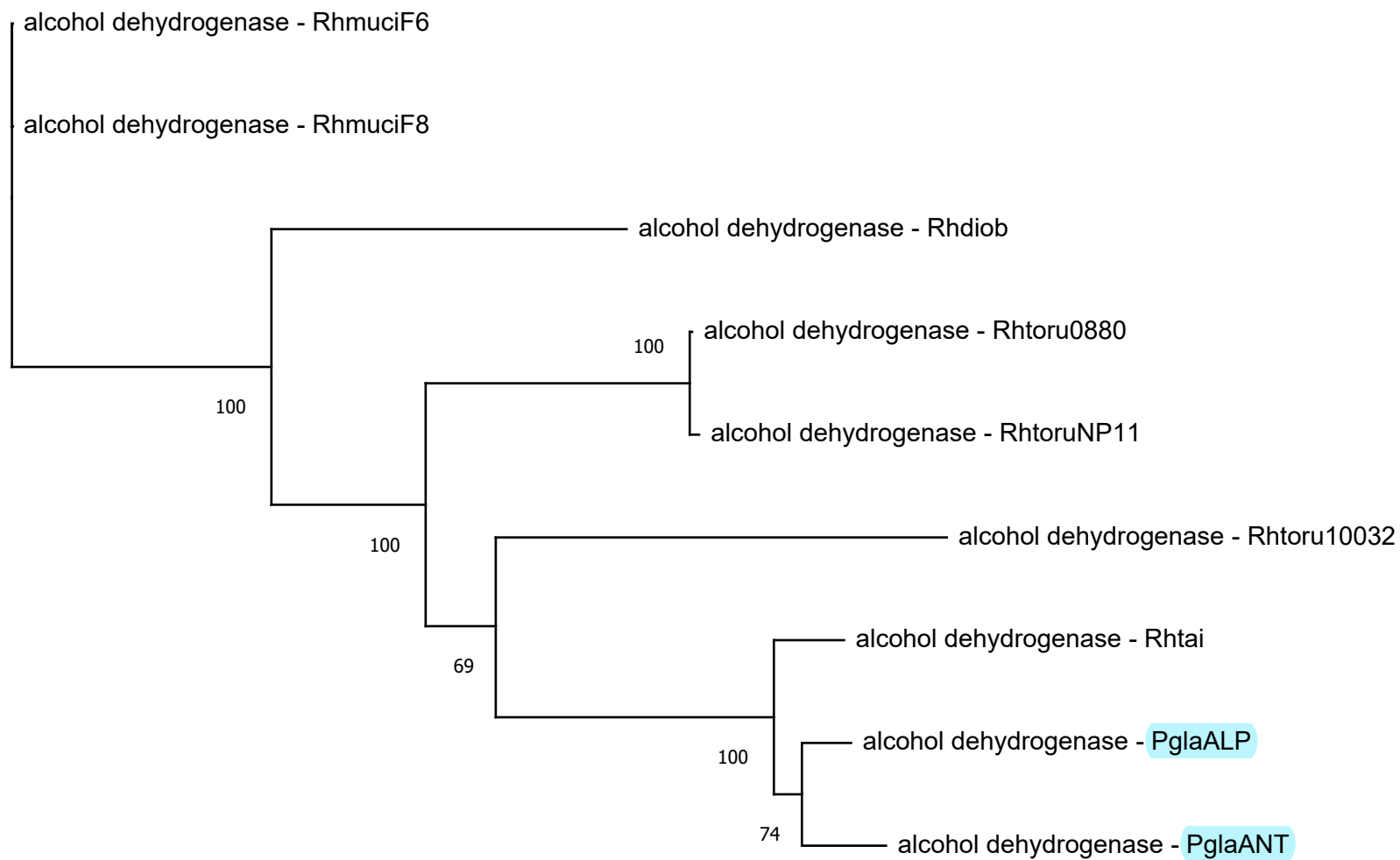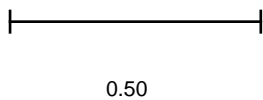

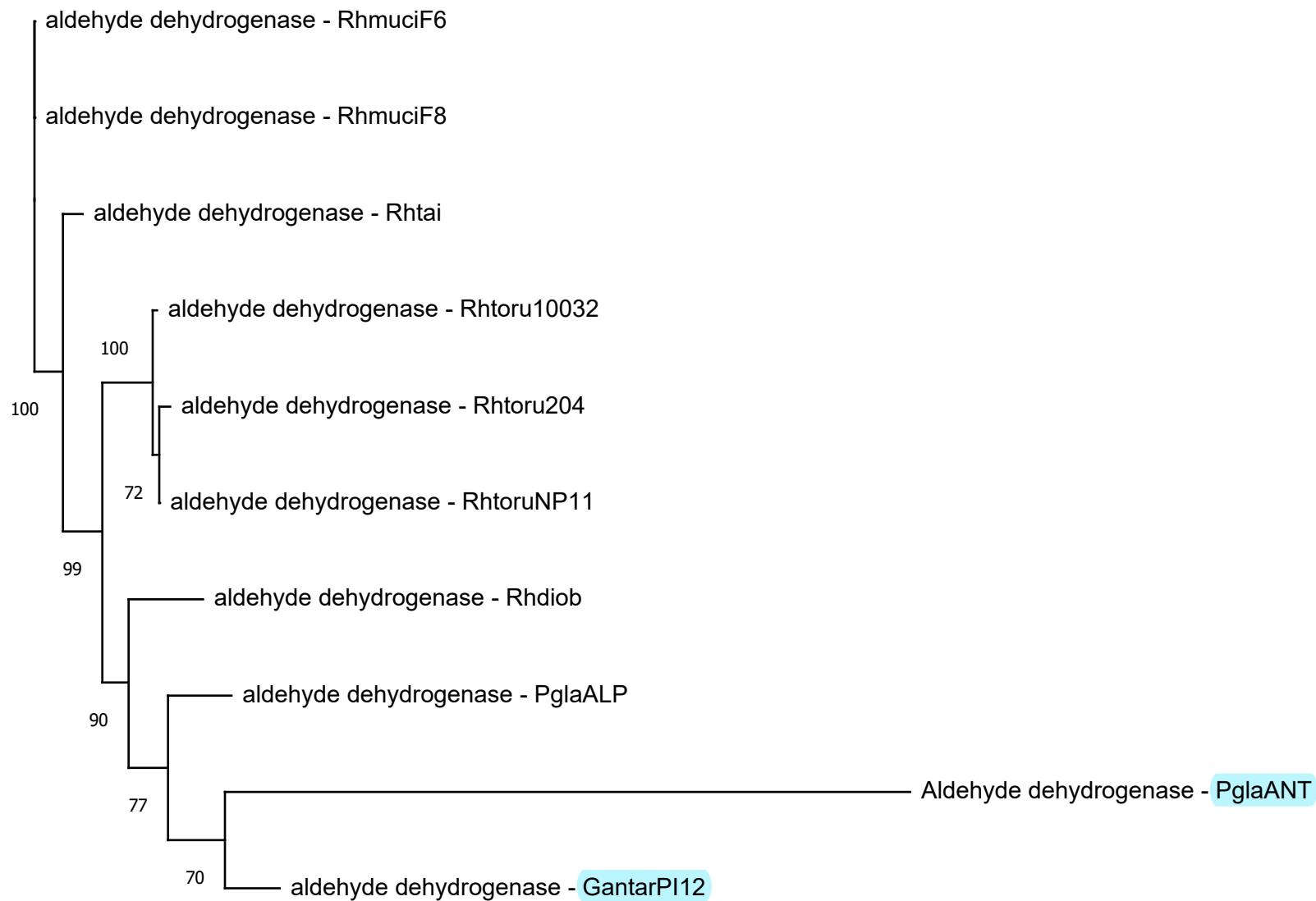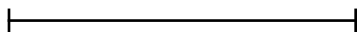

0.50

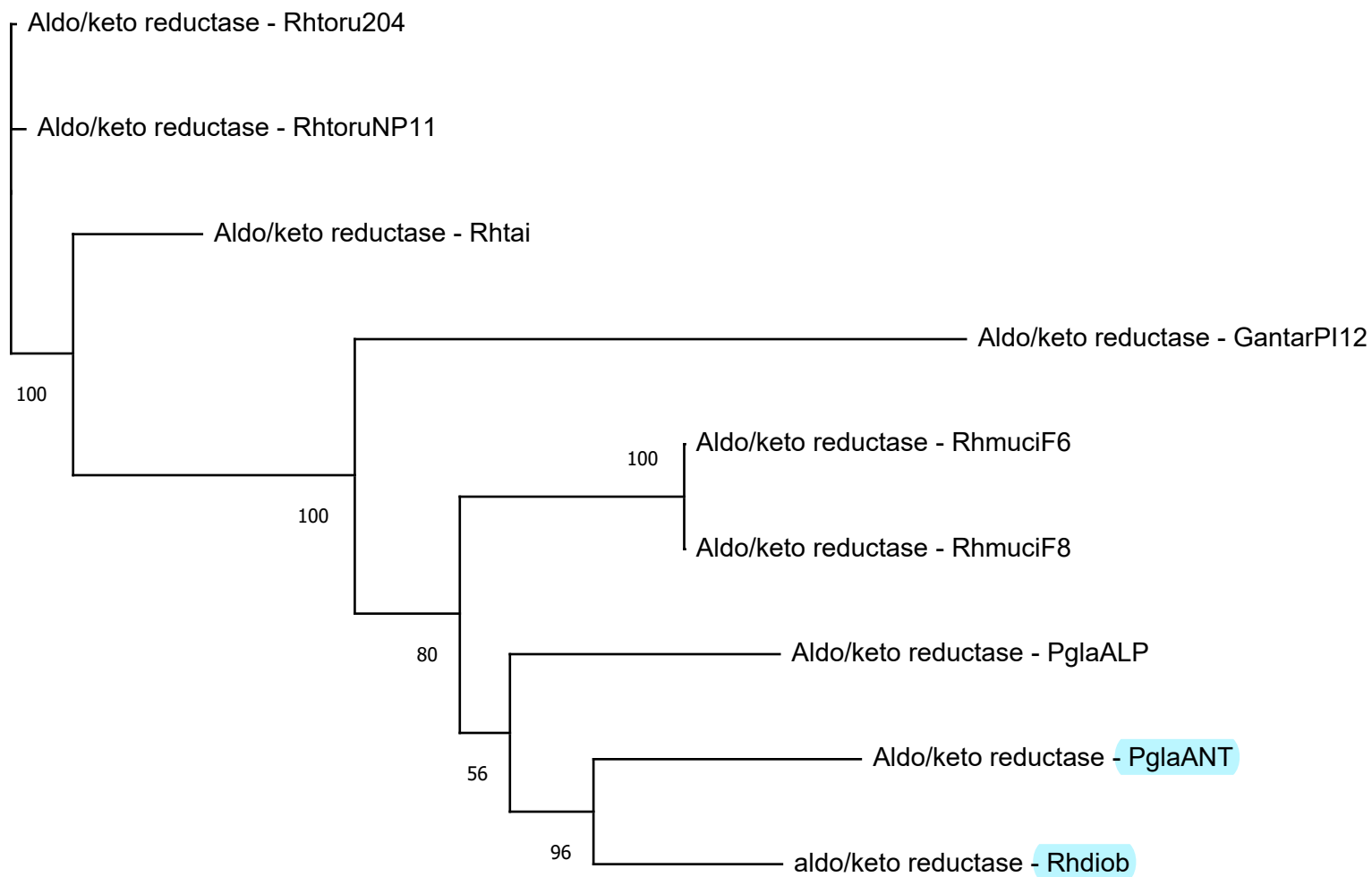

0.50

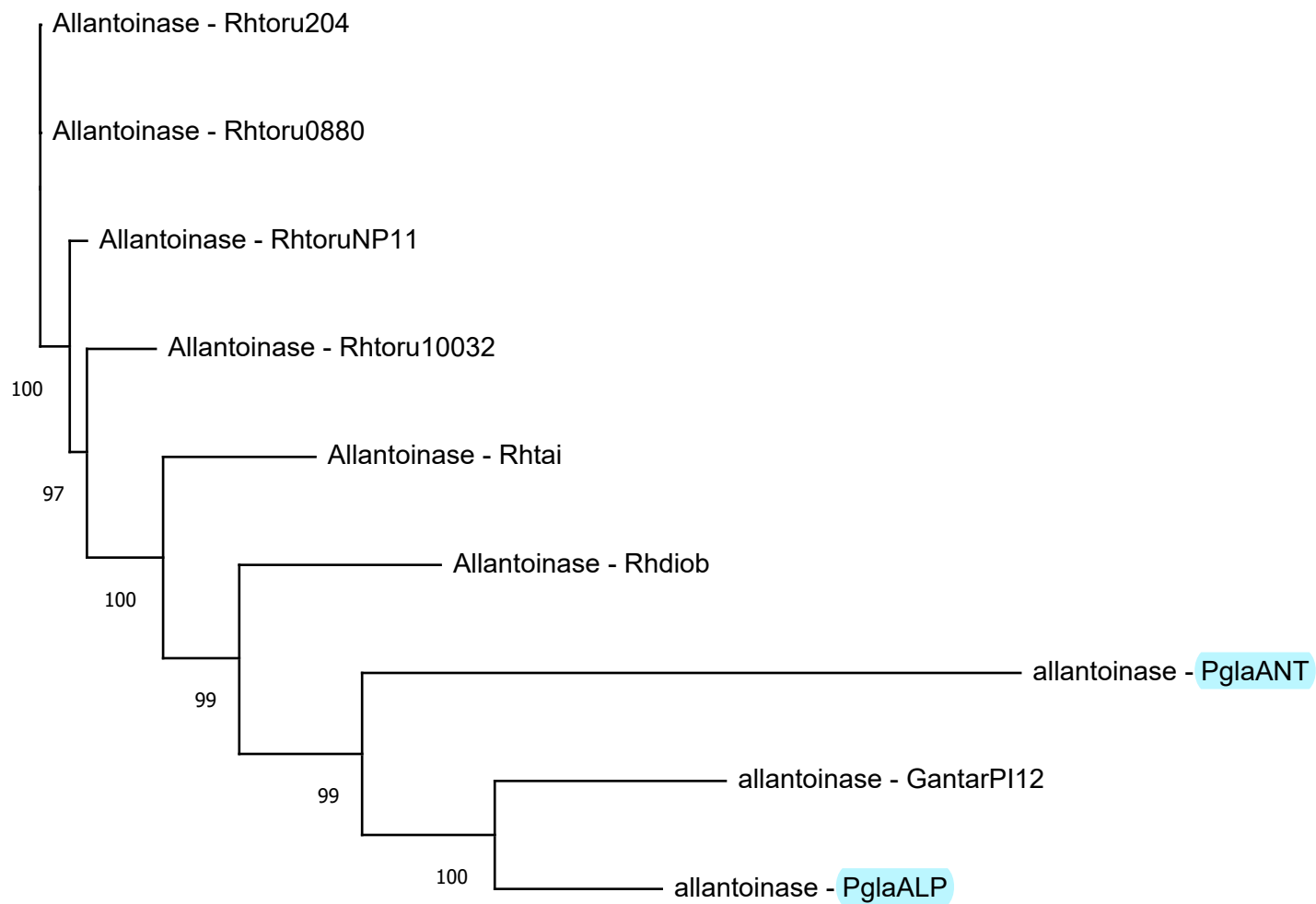

0.10

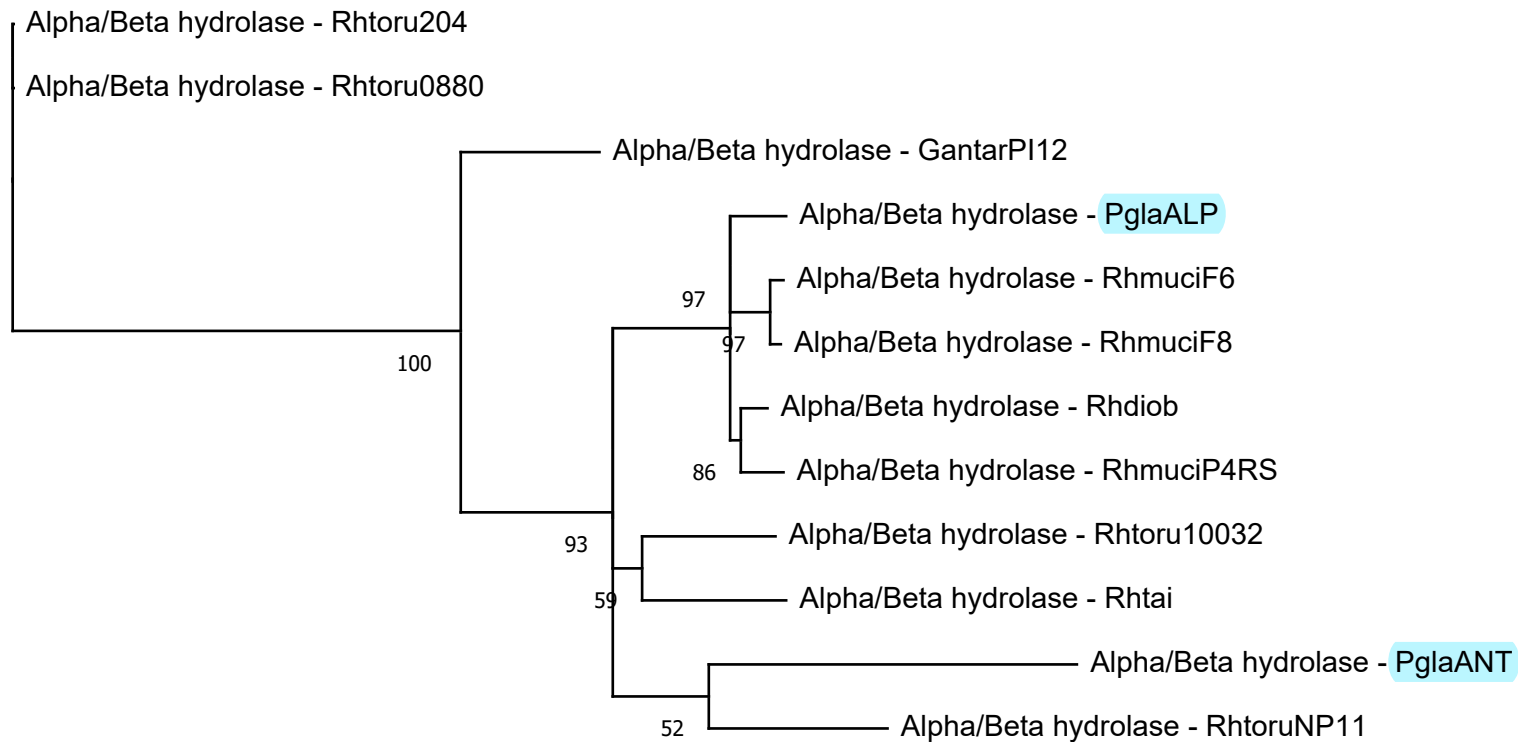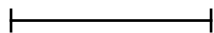

1.00

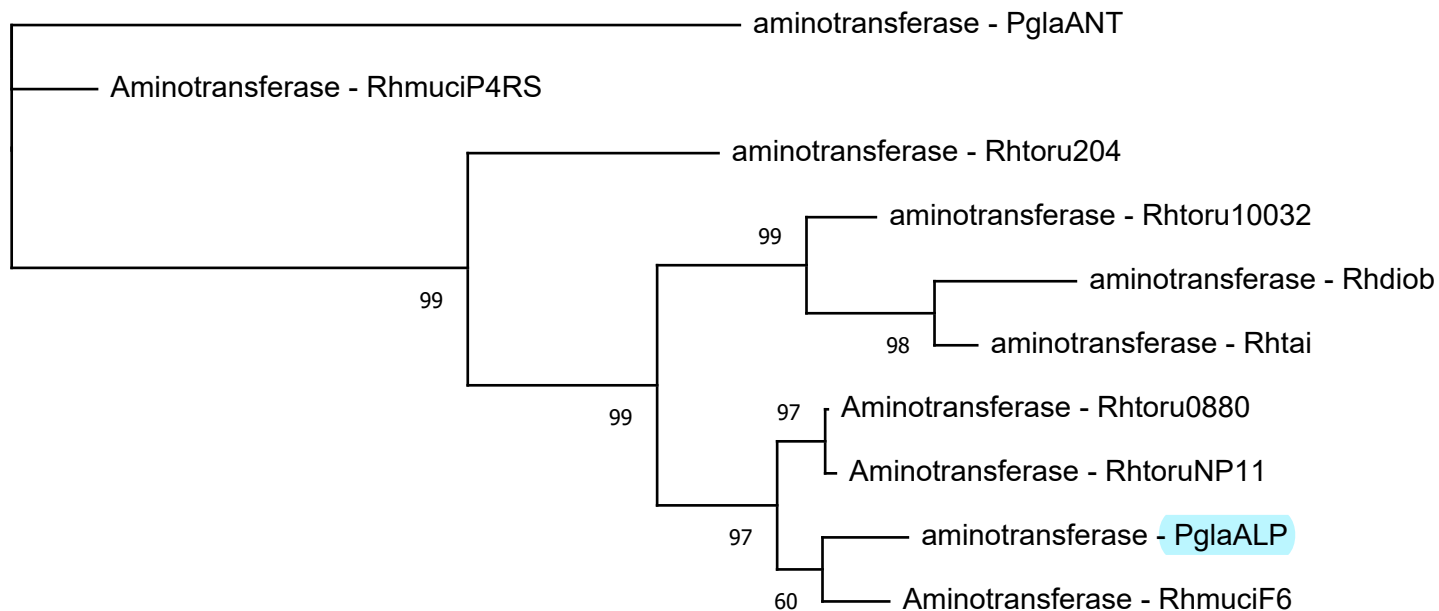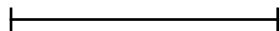

1.00

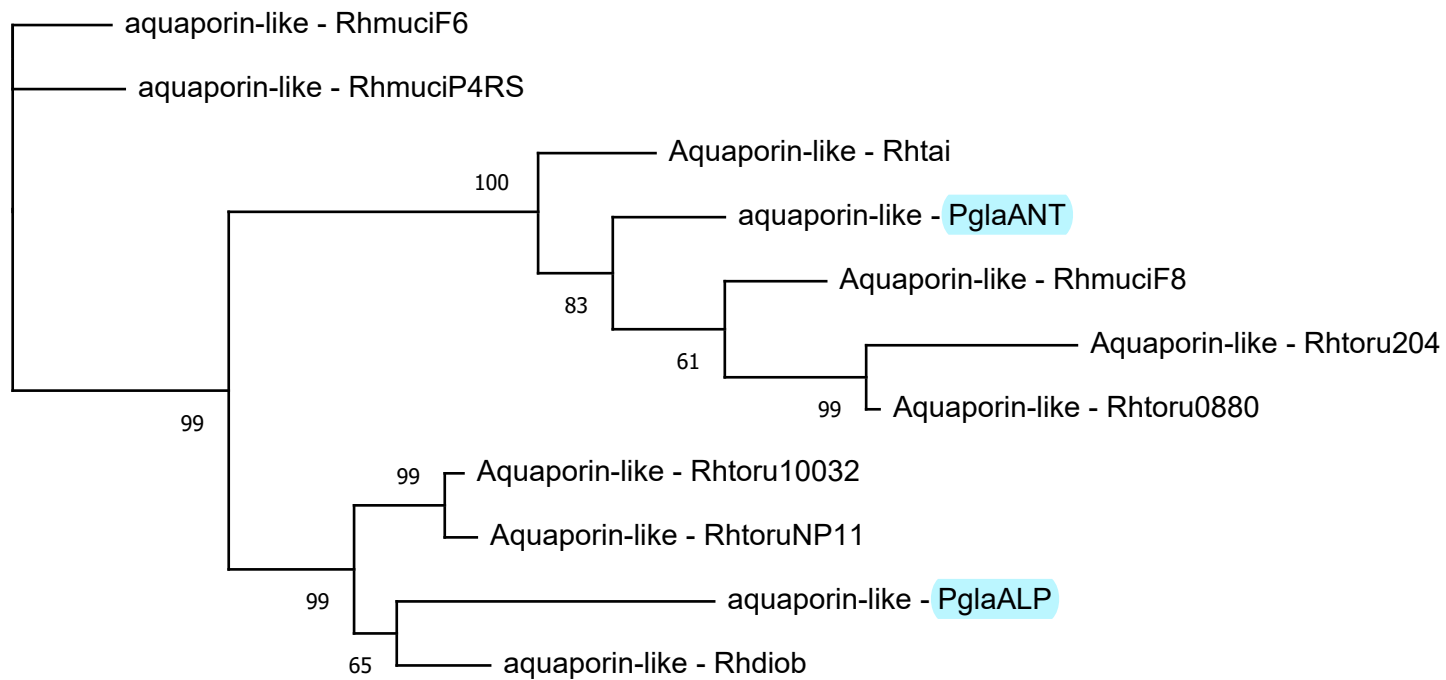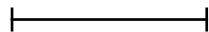

0.50

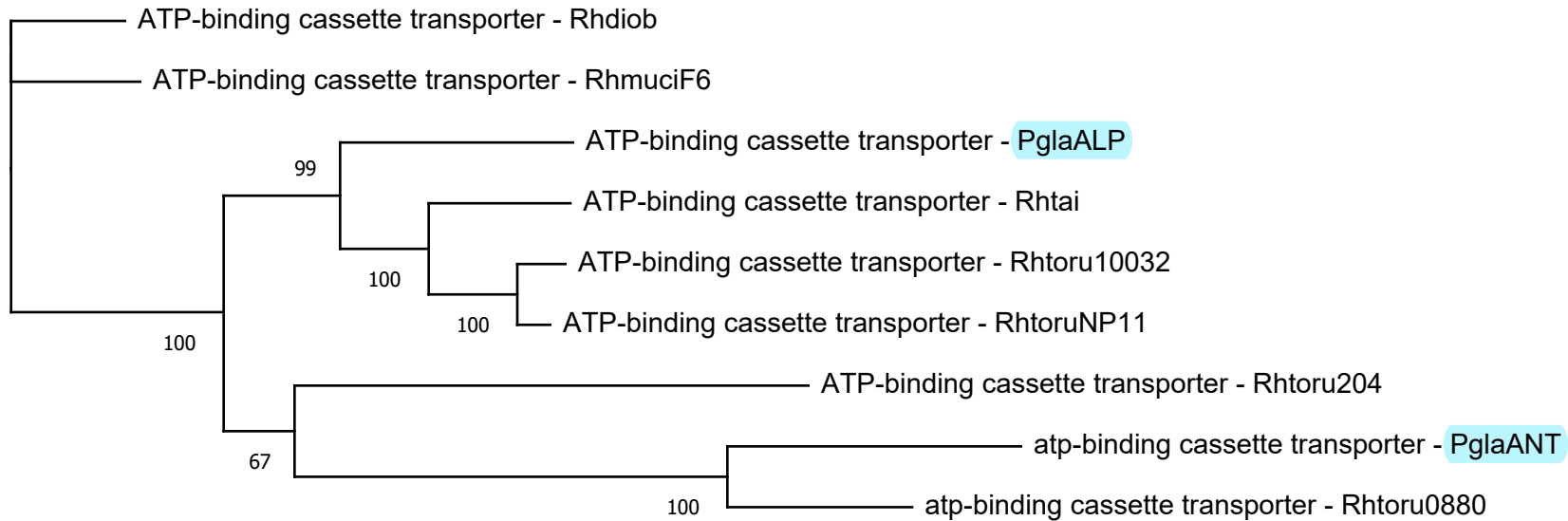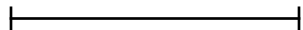

0.50

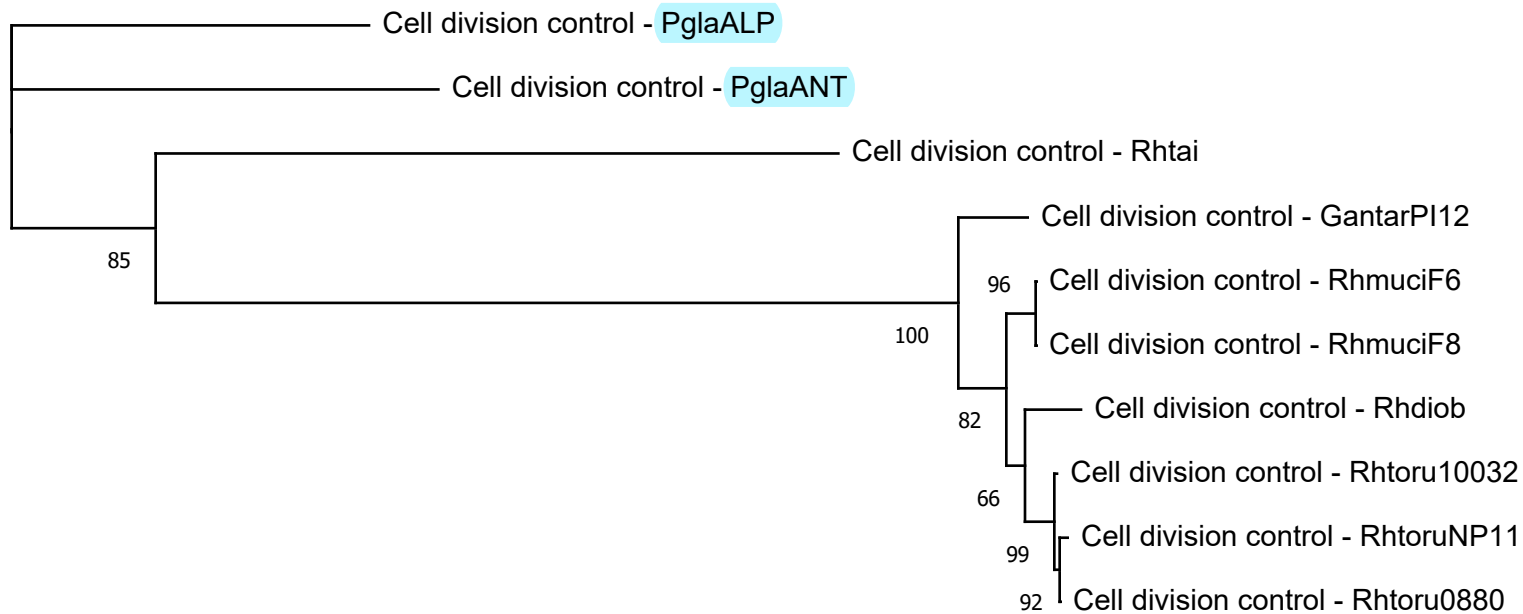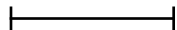

0.20

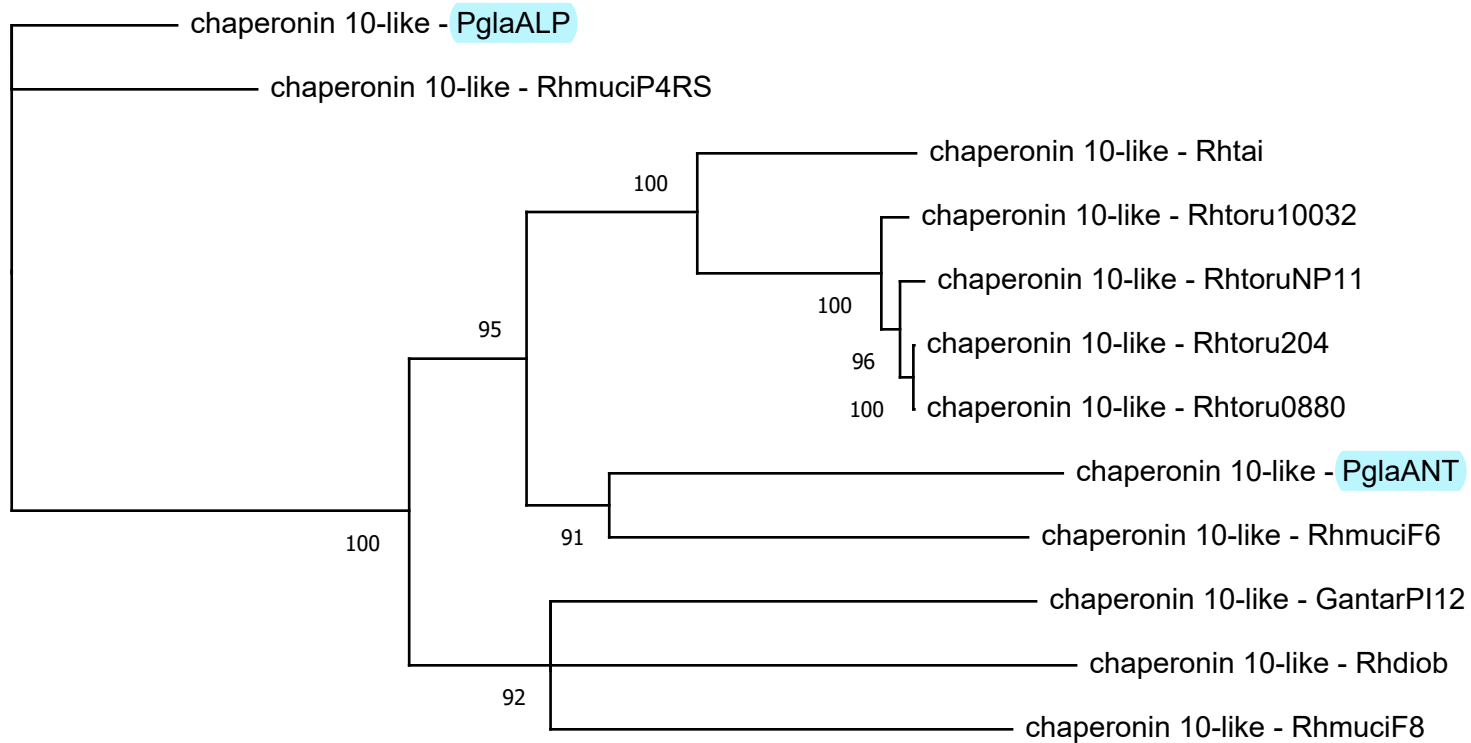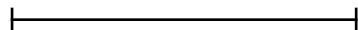

0.50

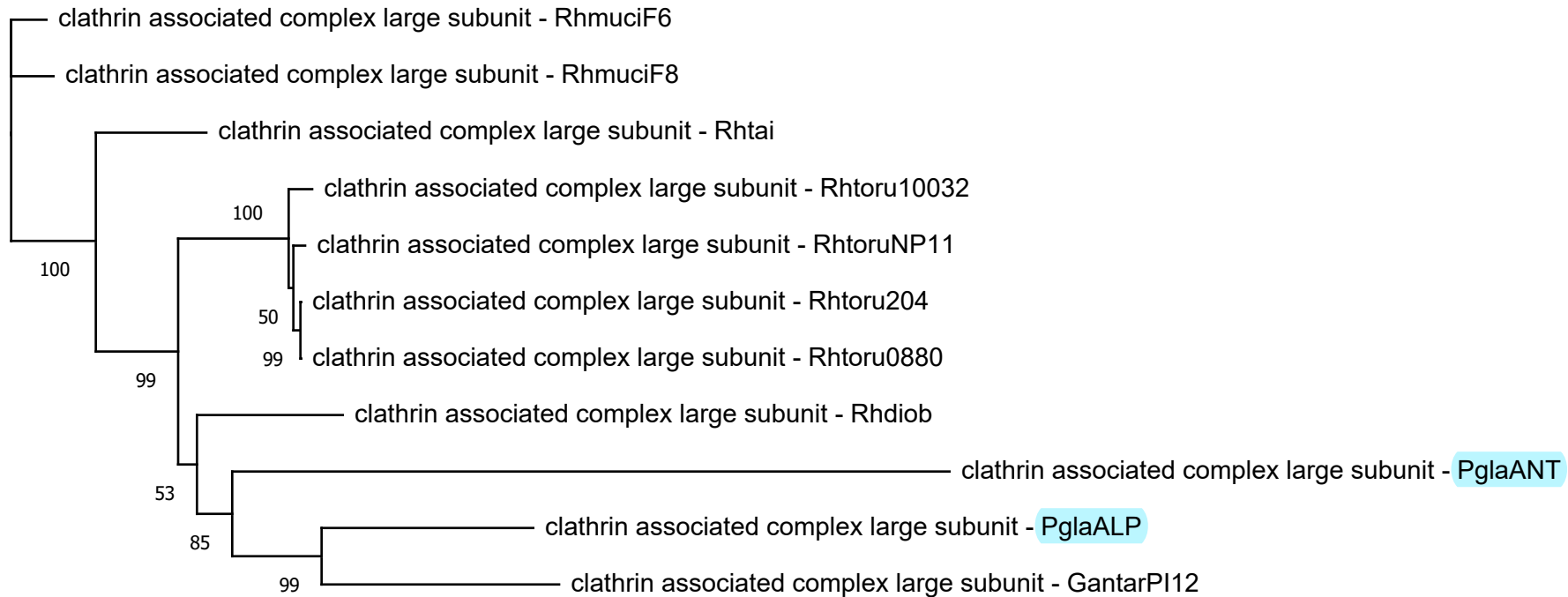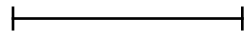

0.10

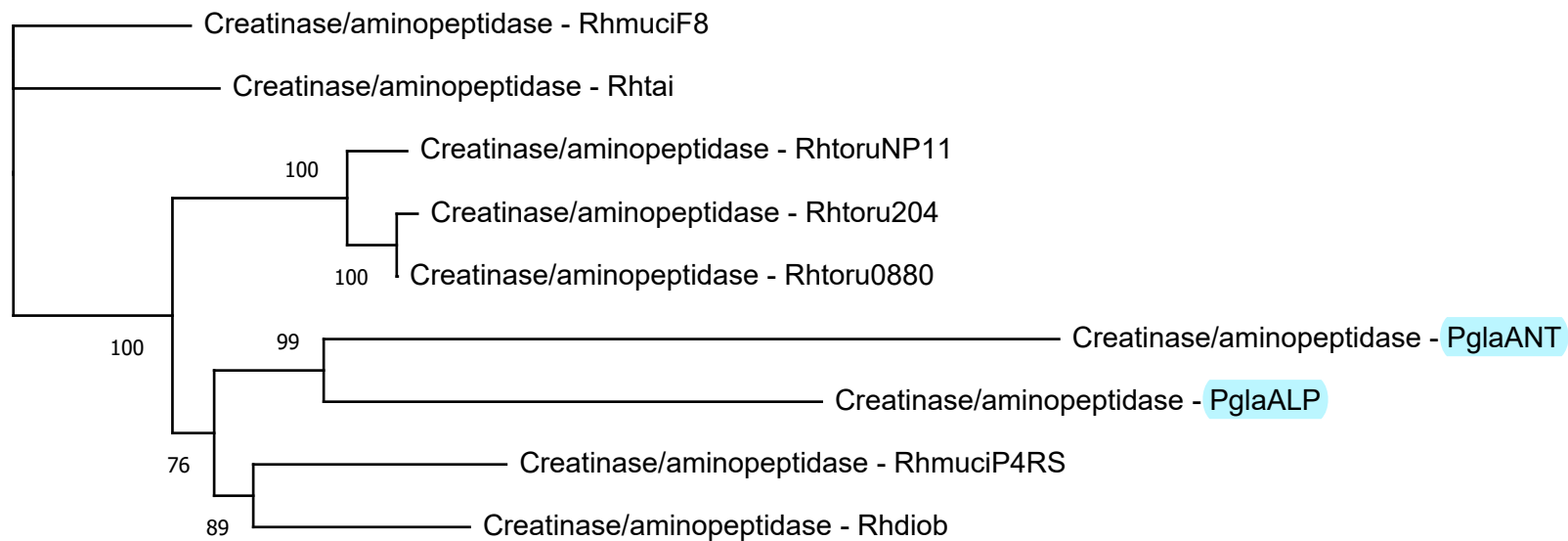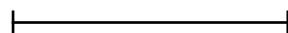

0.20

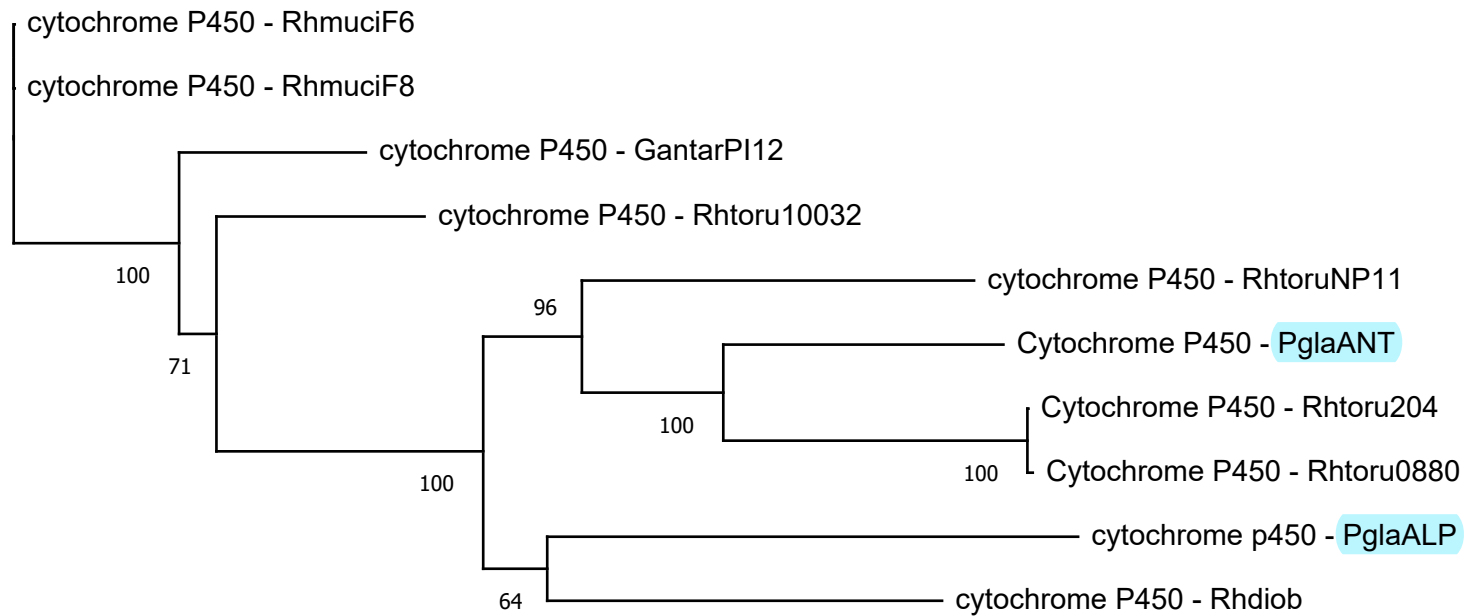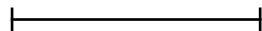

0.50

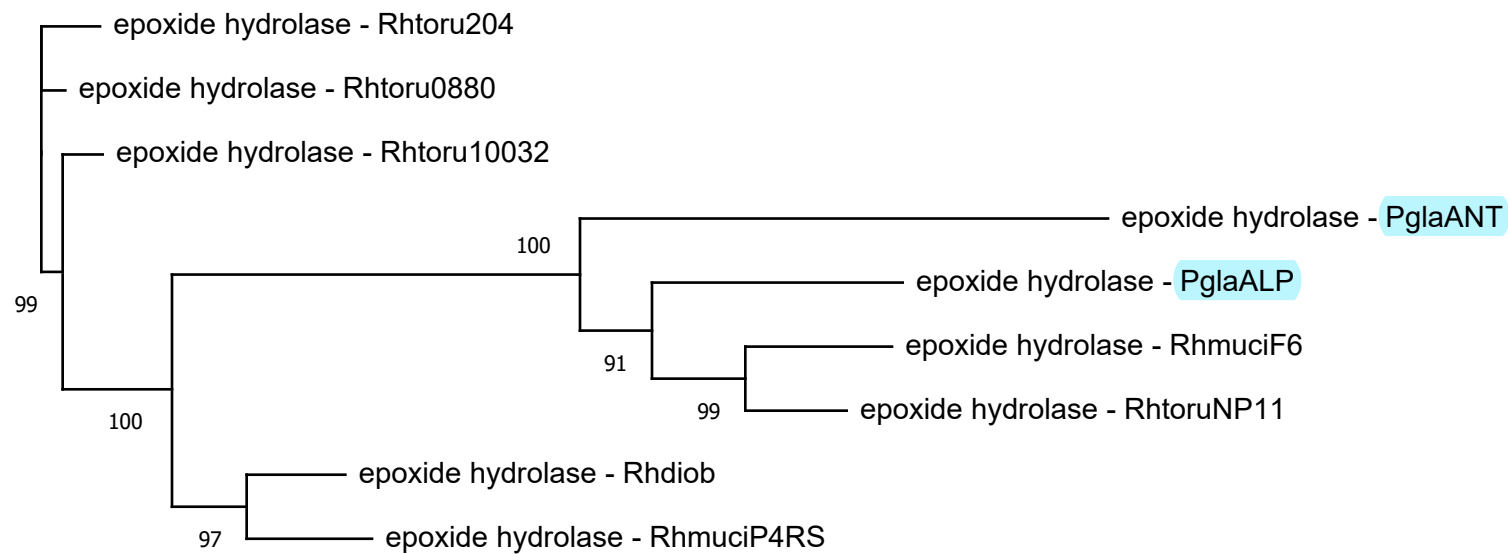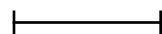

0.20

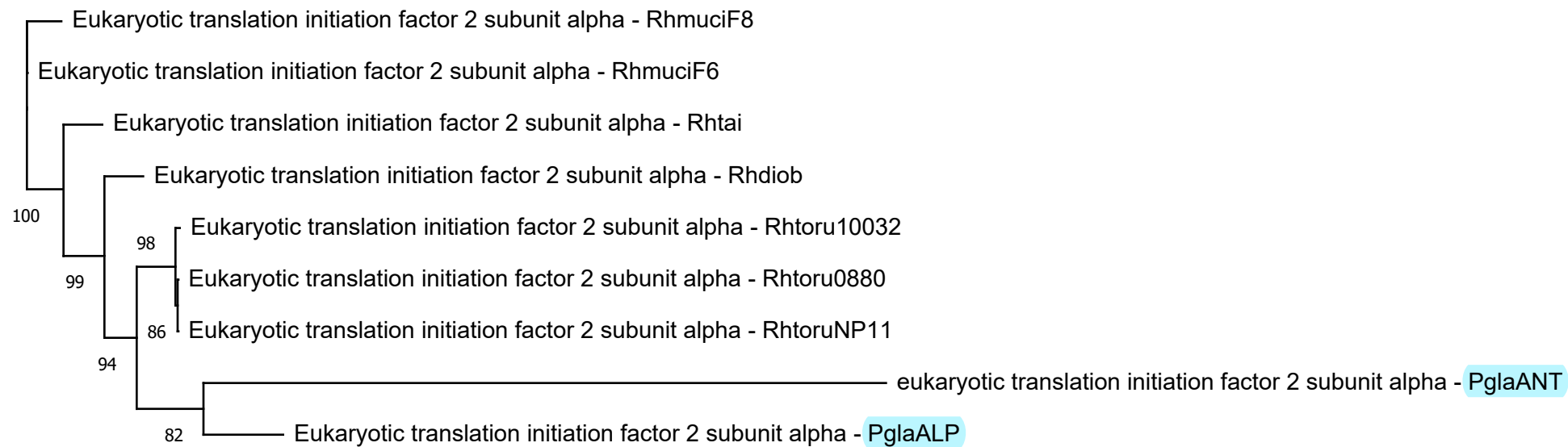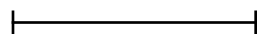

0.20

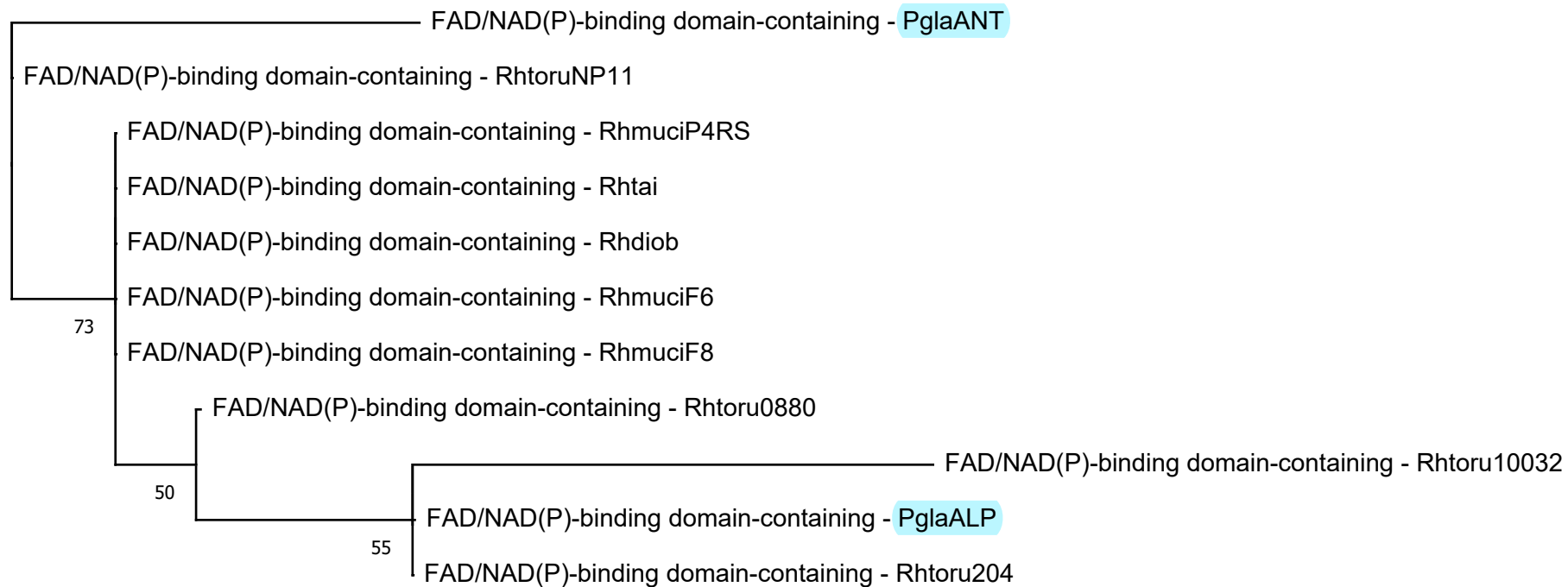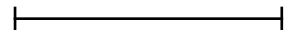

20.00

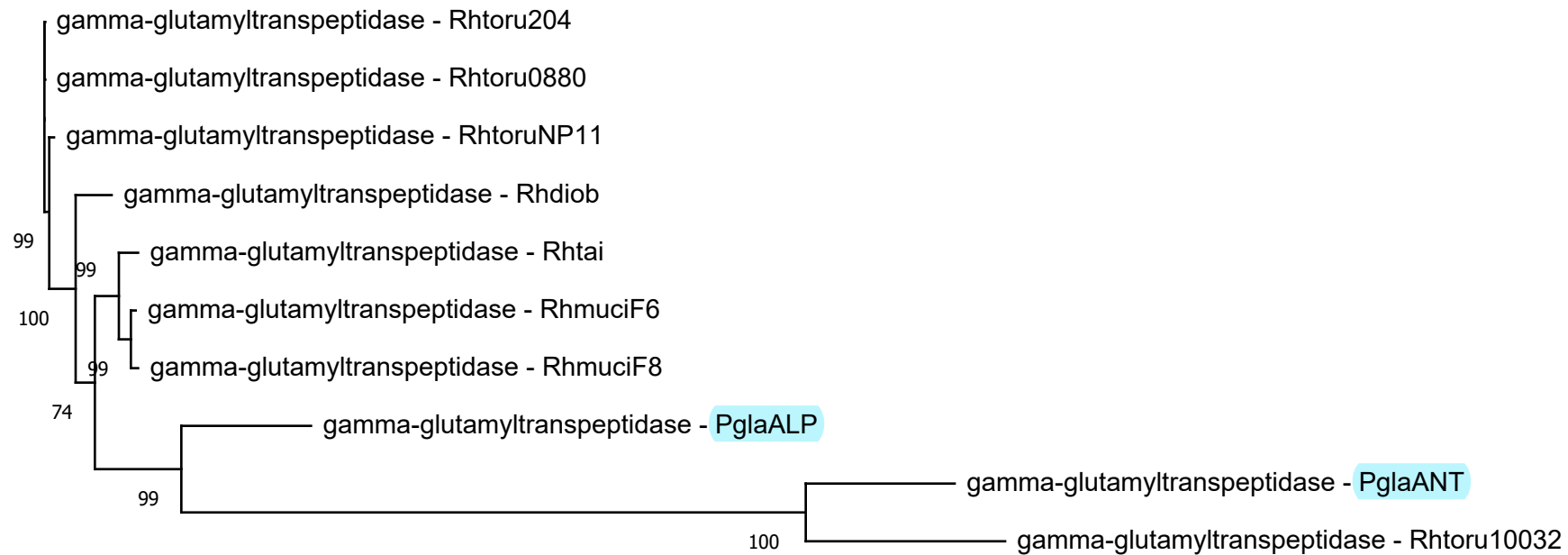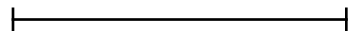

0.50

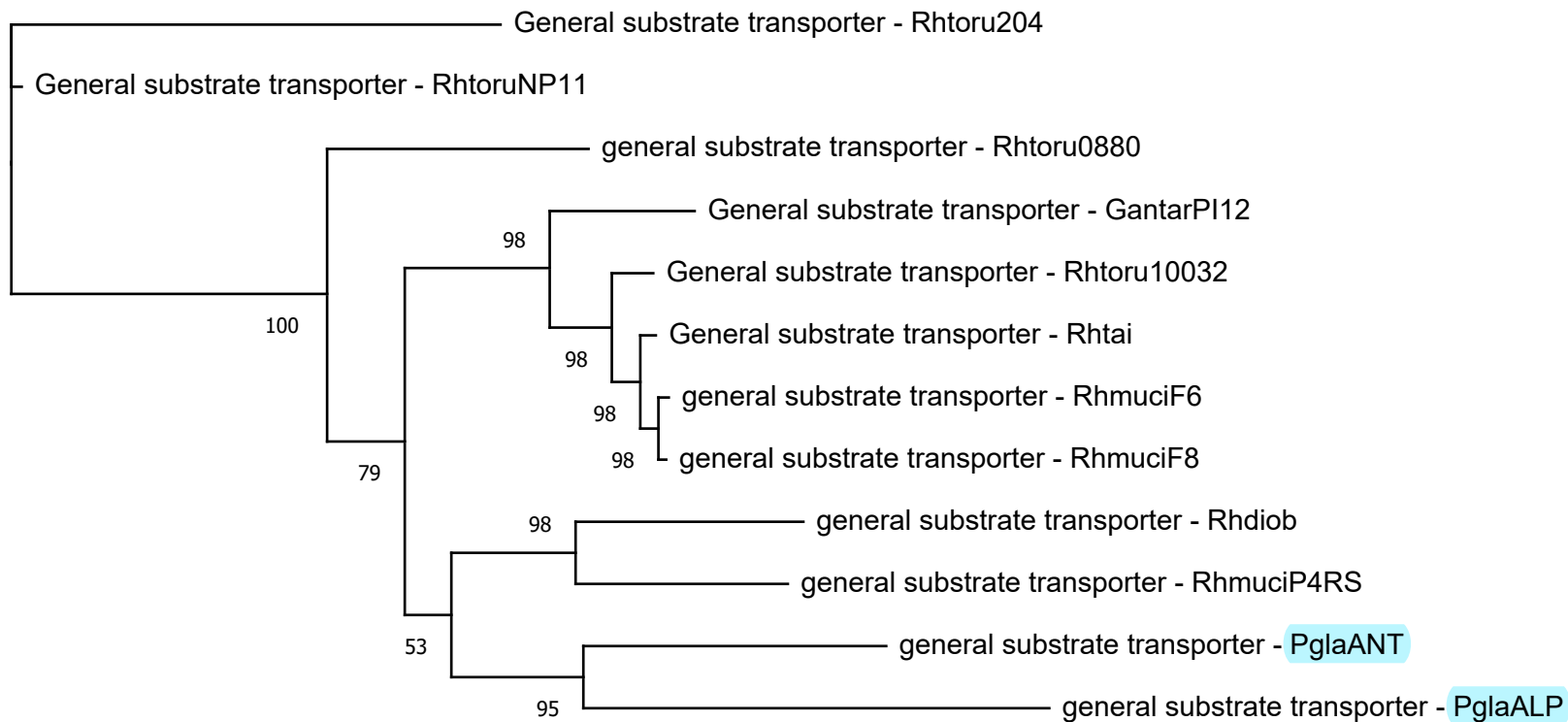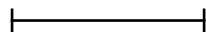

0.50

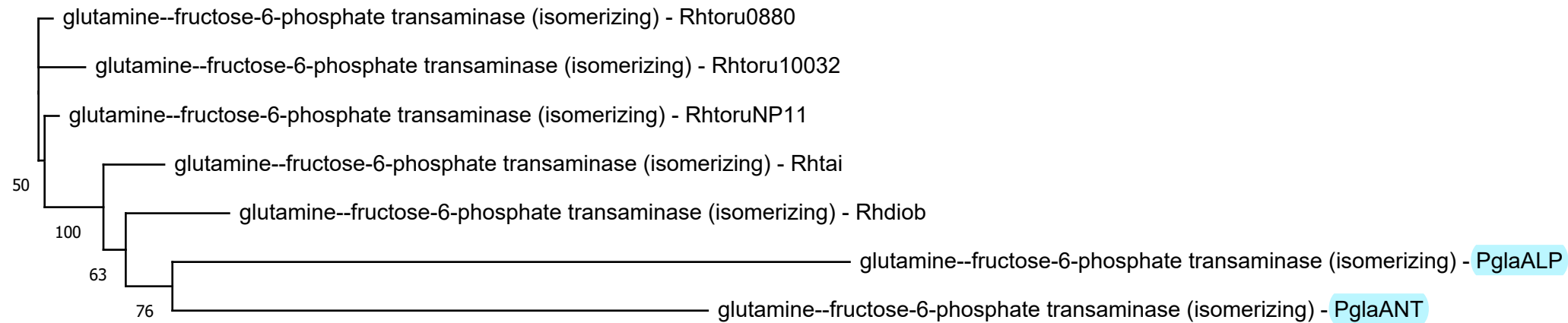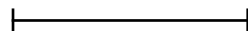

0.10

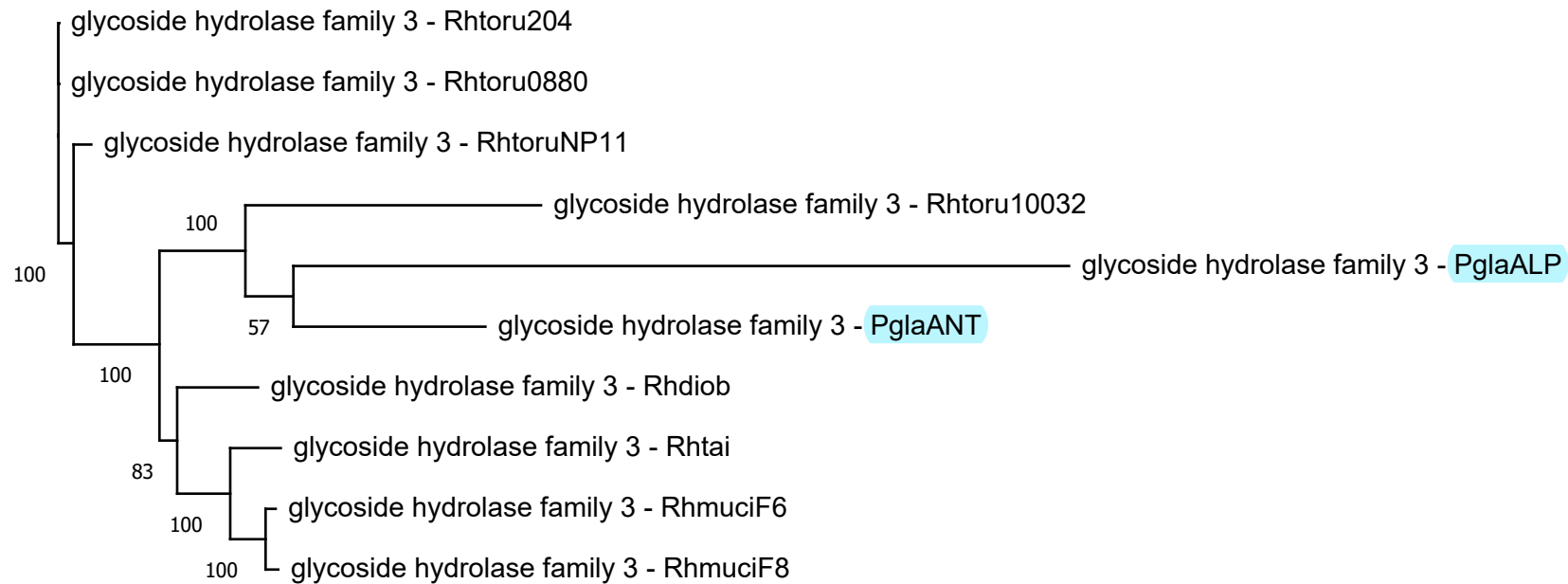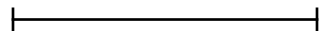

0.50

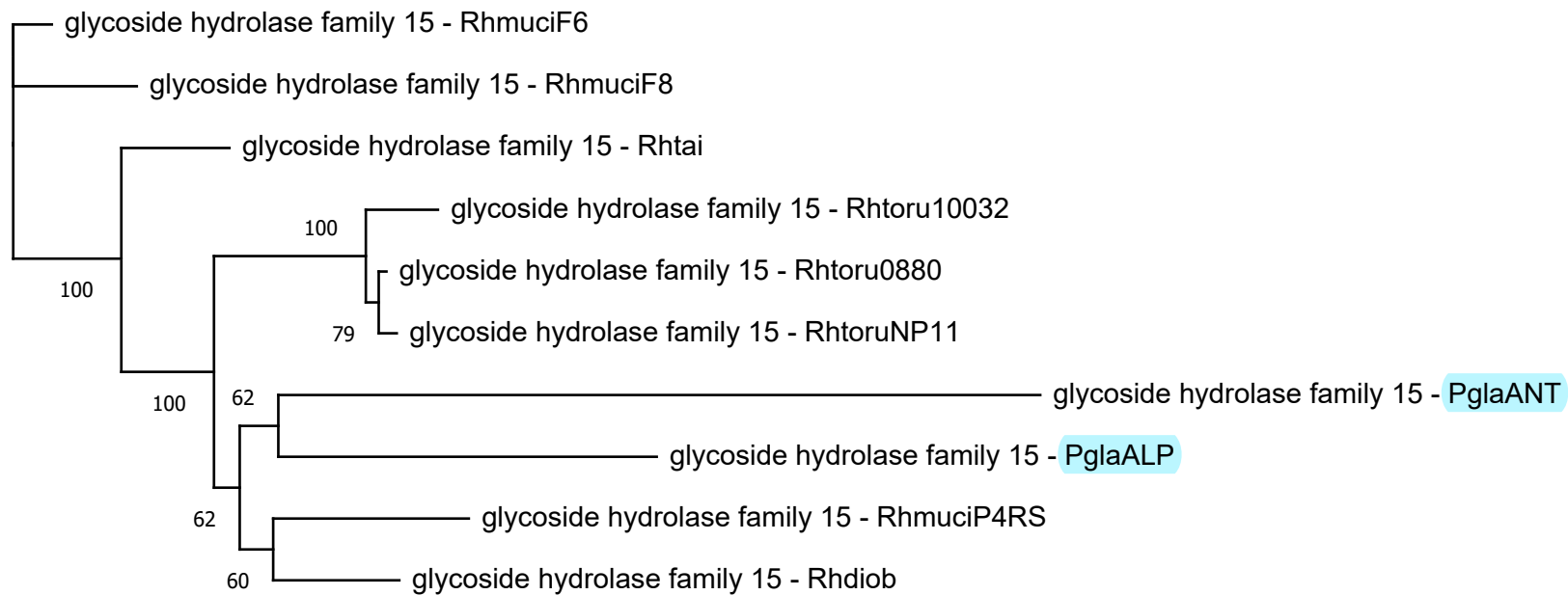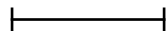

0.10

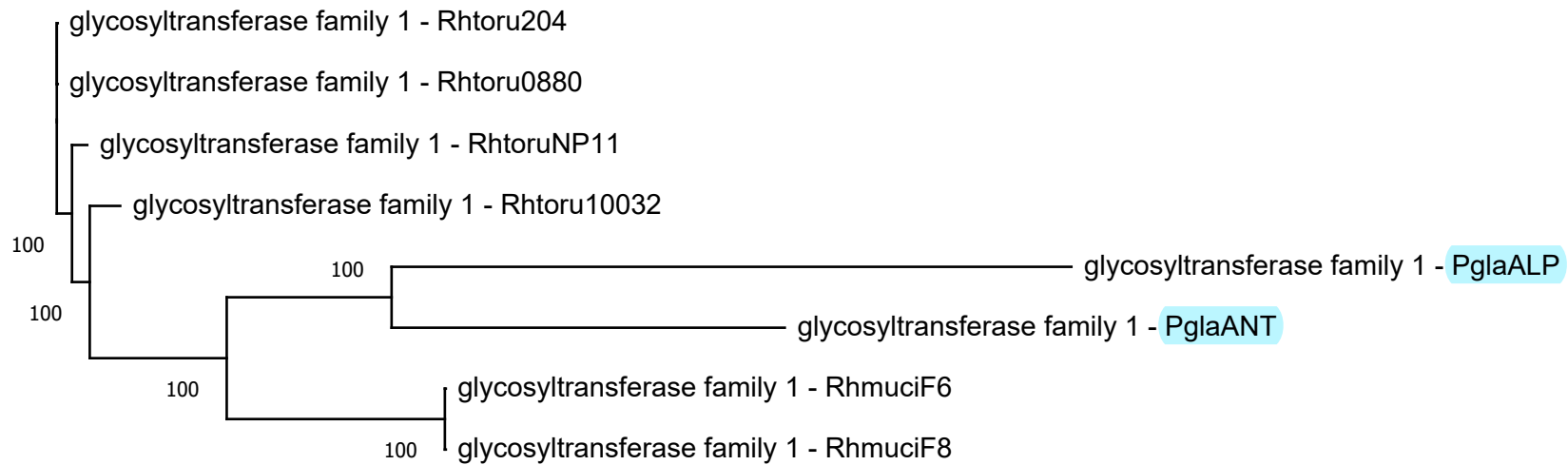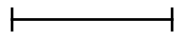

0.20

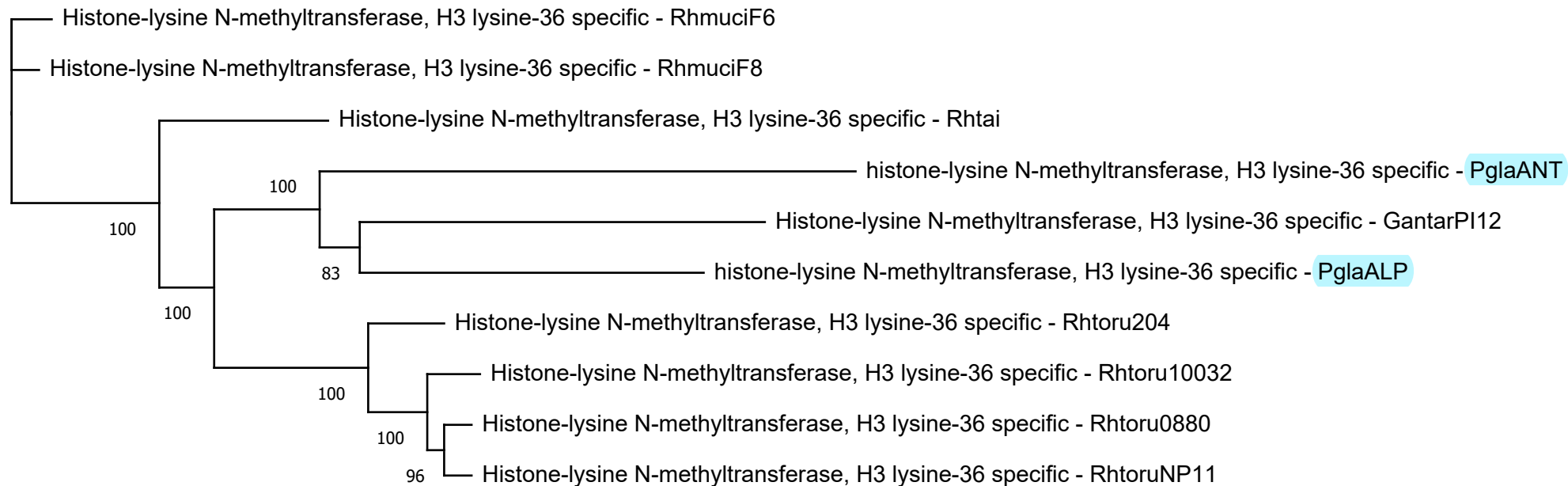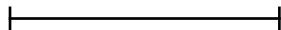

0.20

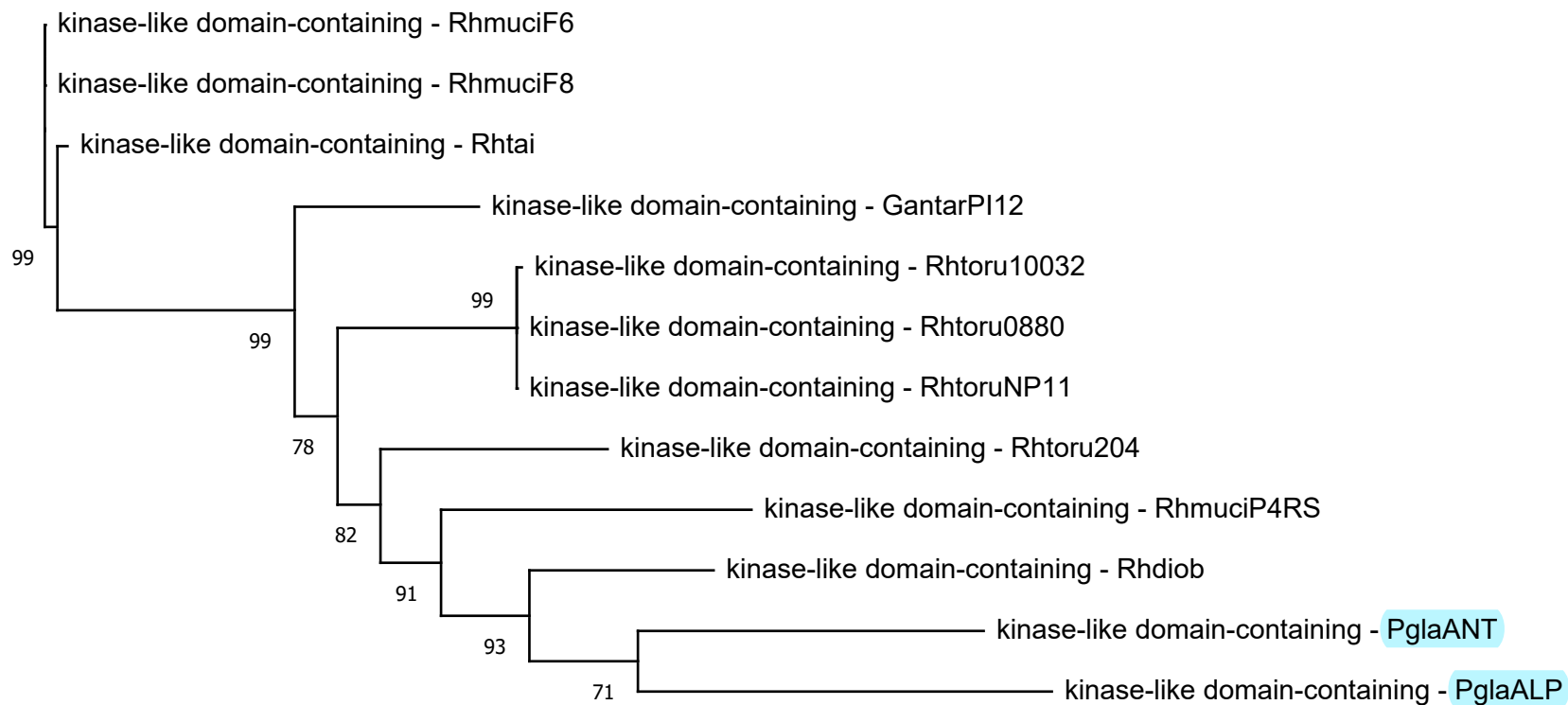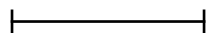

0.50

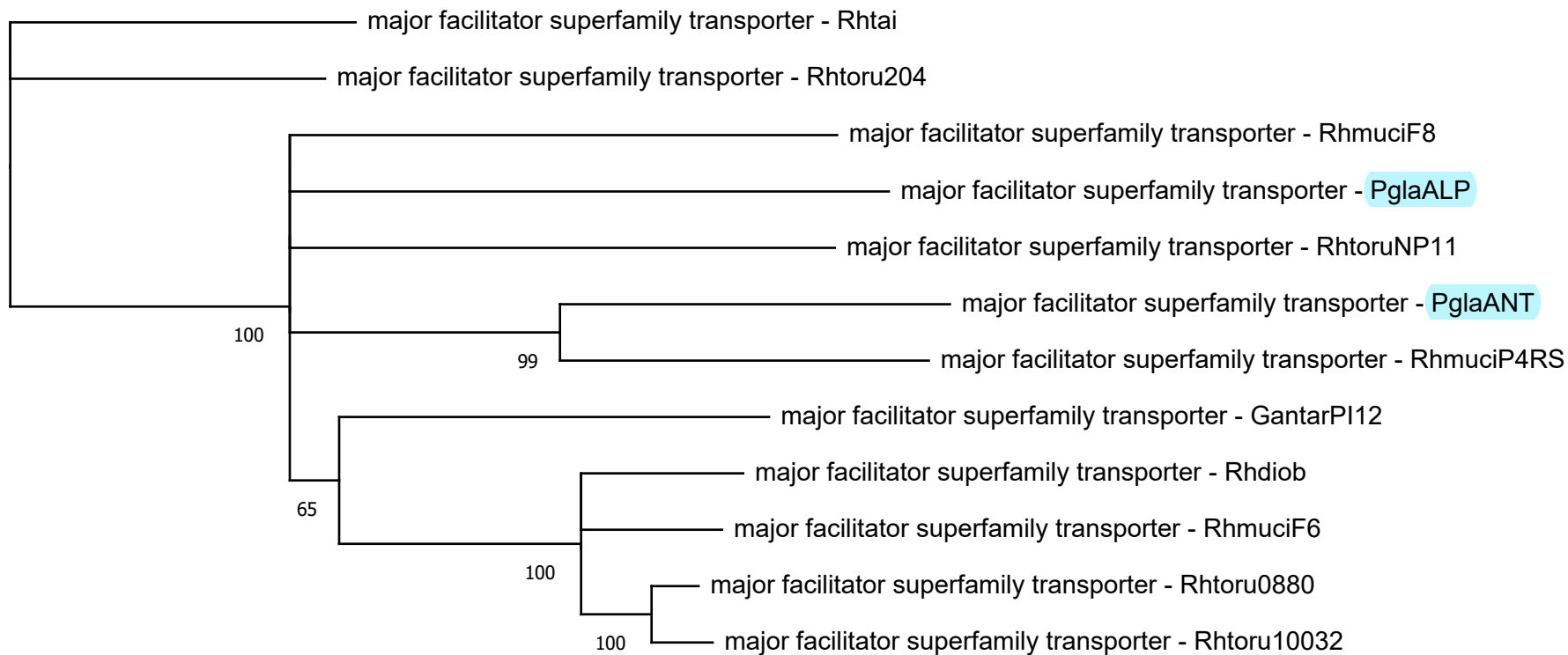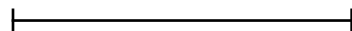

0.50

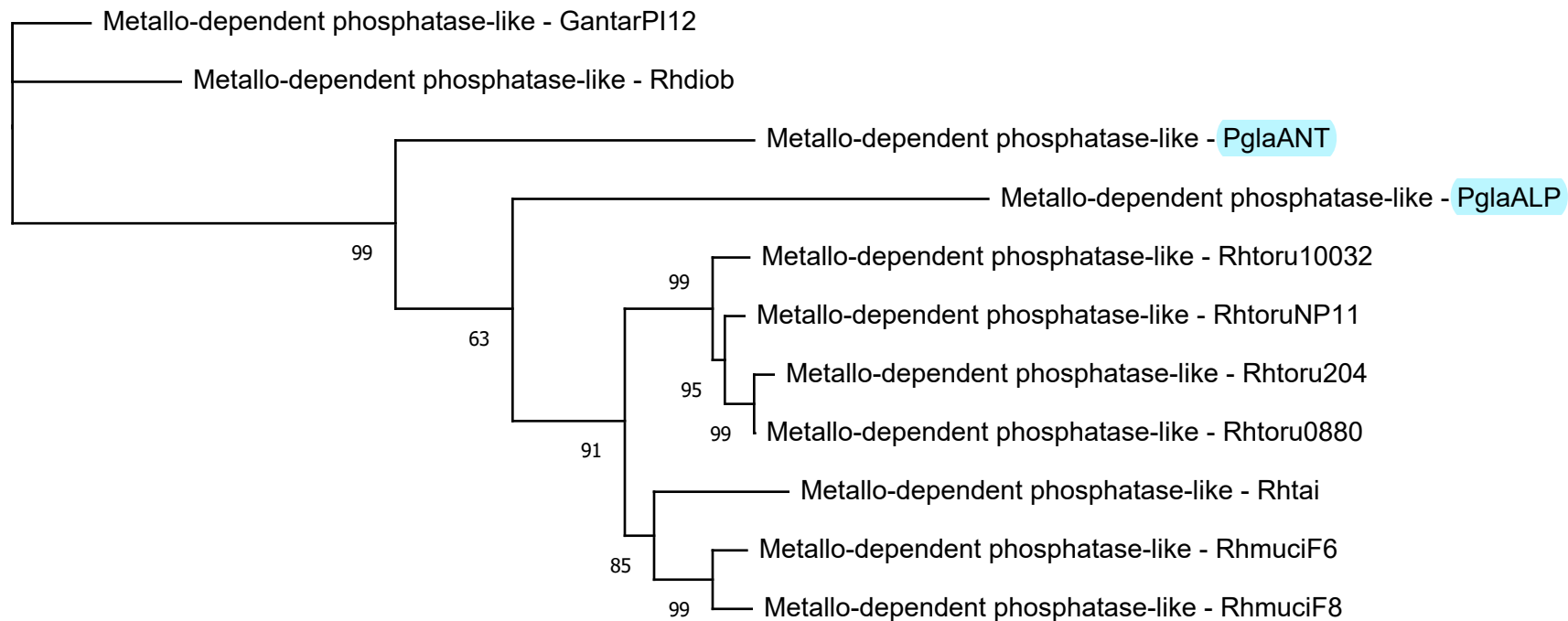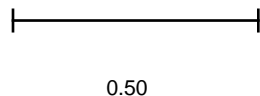

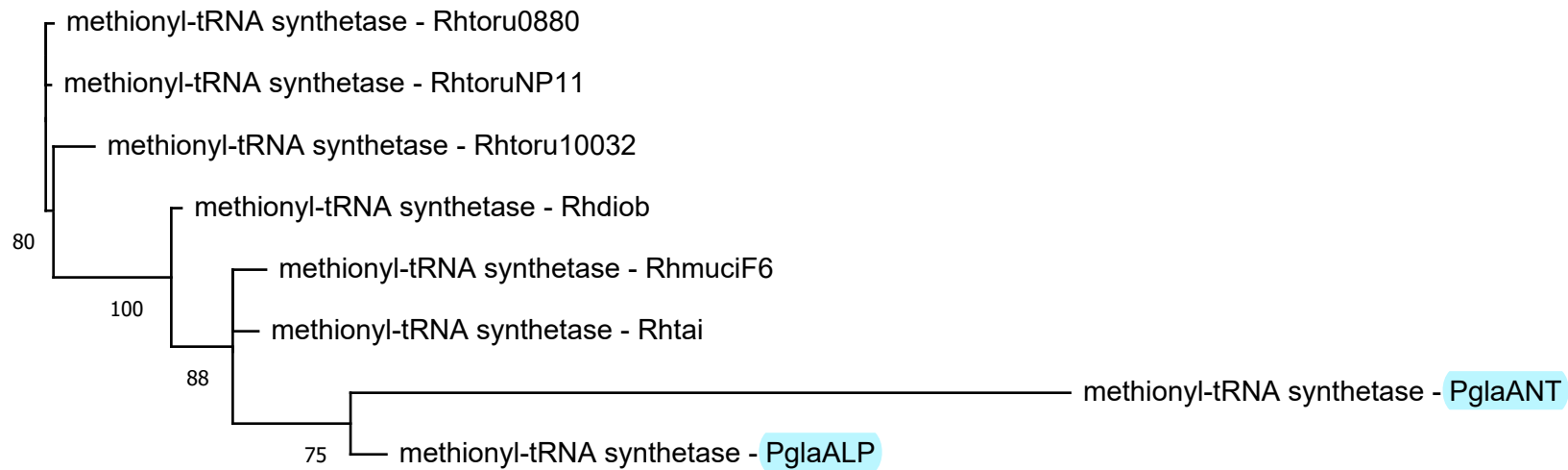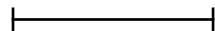

0.50

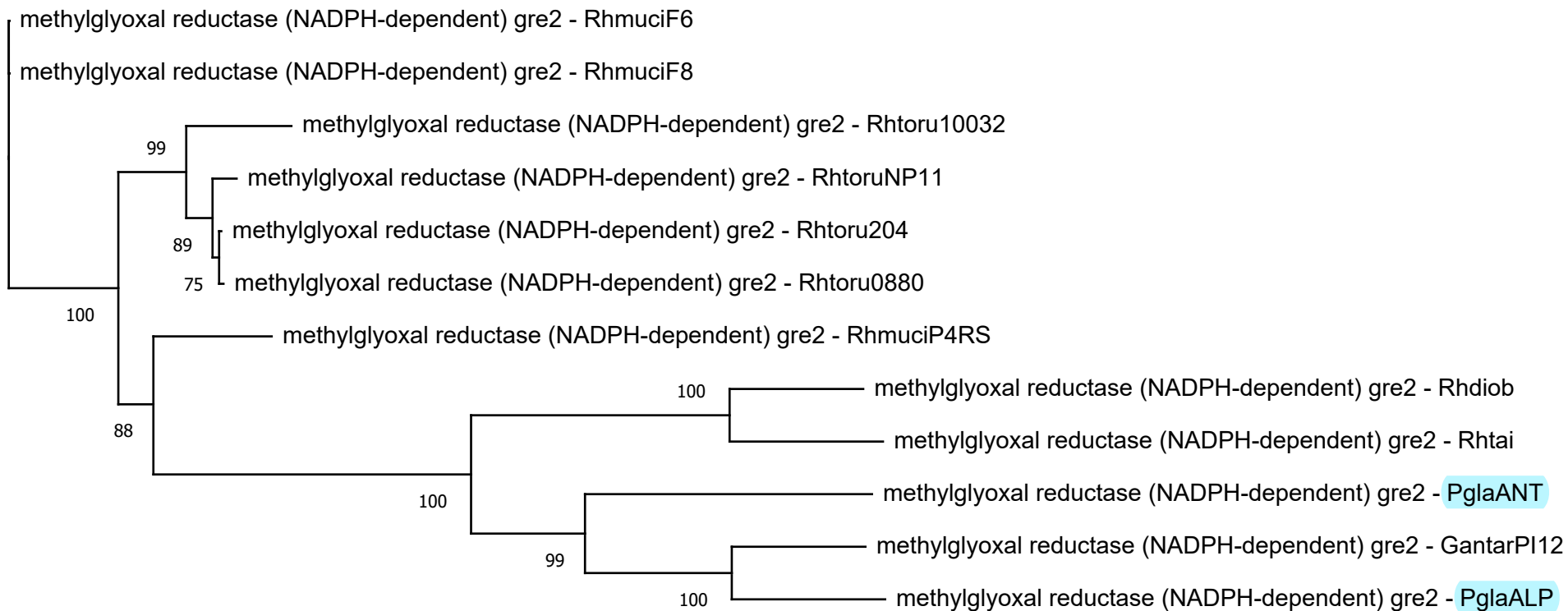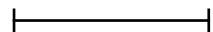

0.20

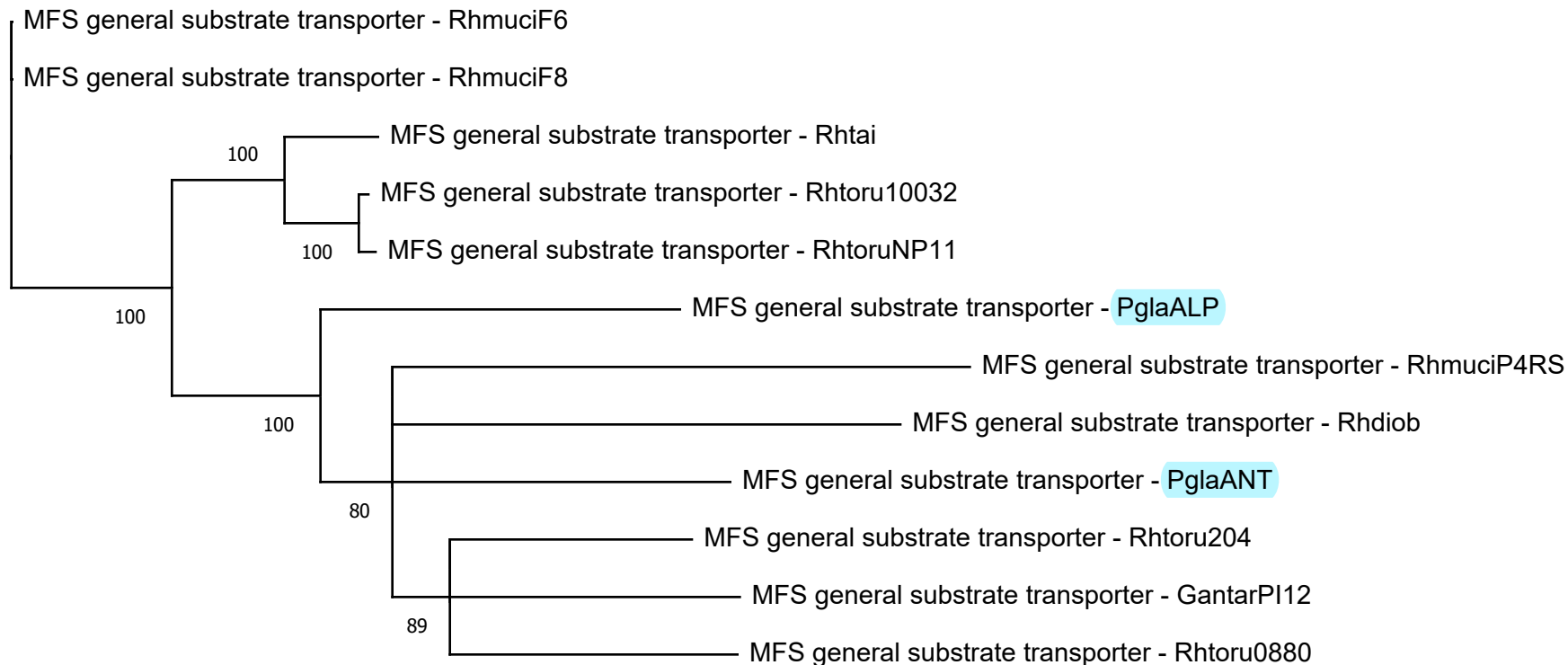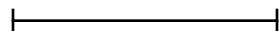

0.50

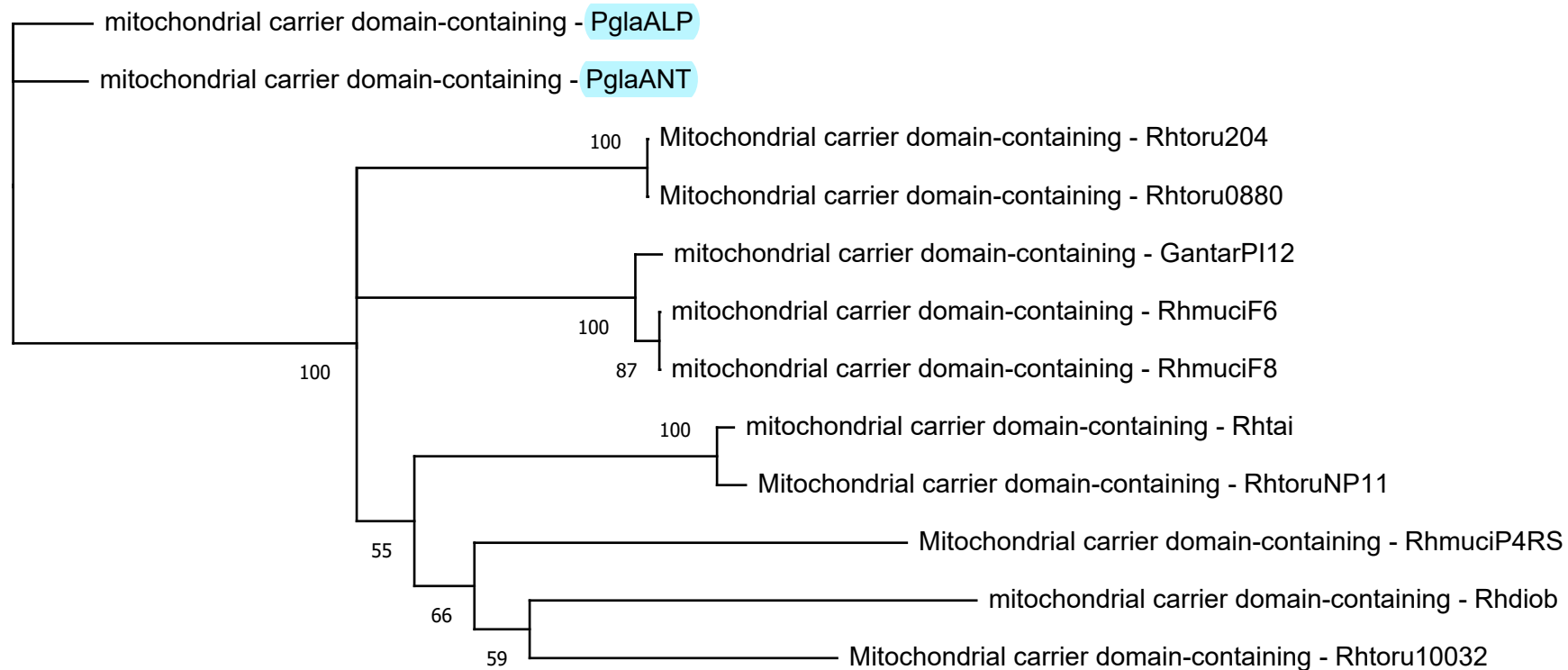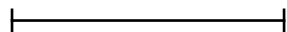

0.50

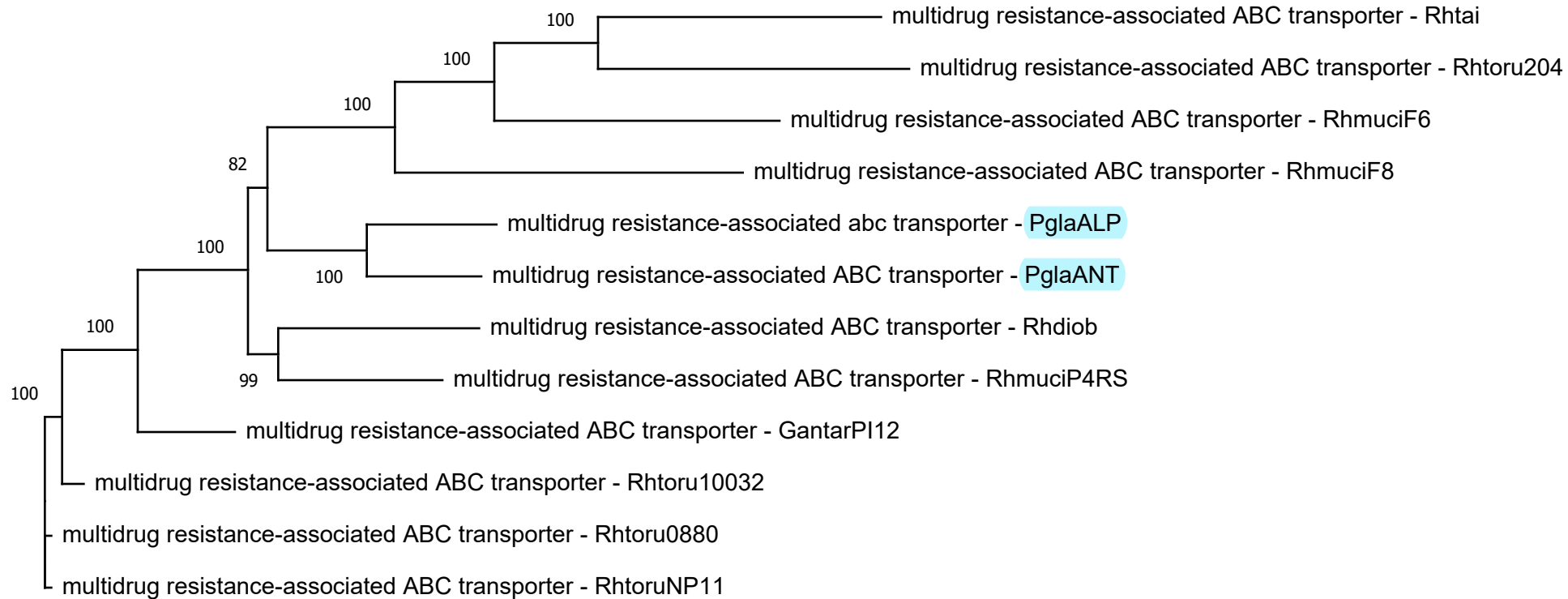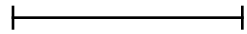

0.50

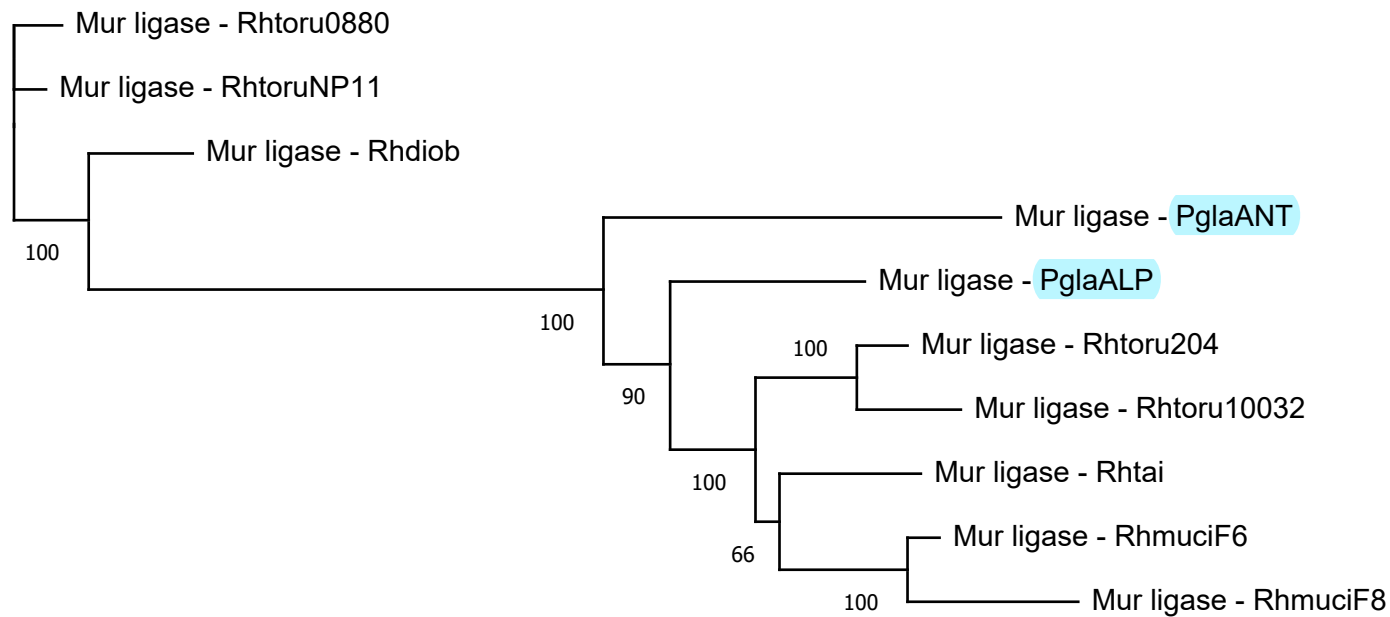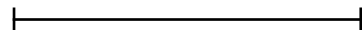

0.50

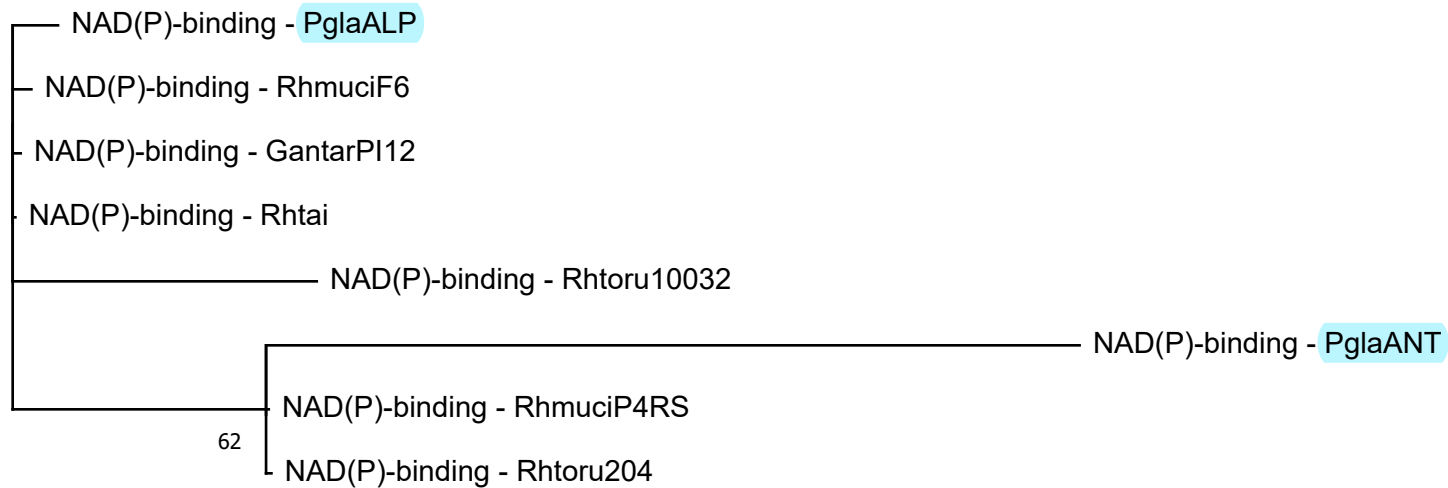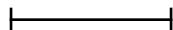

10.00

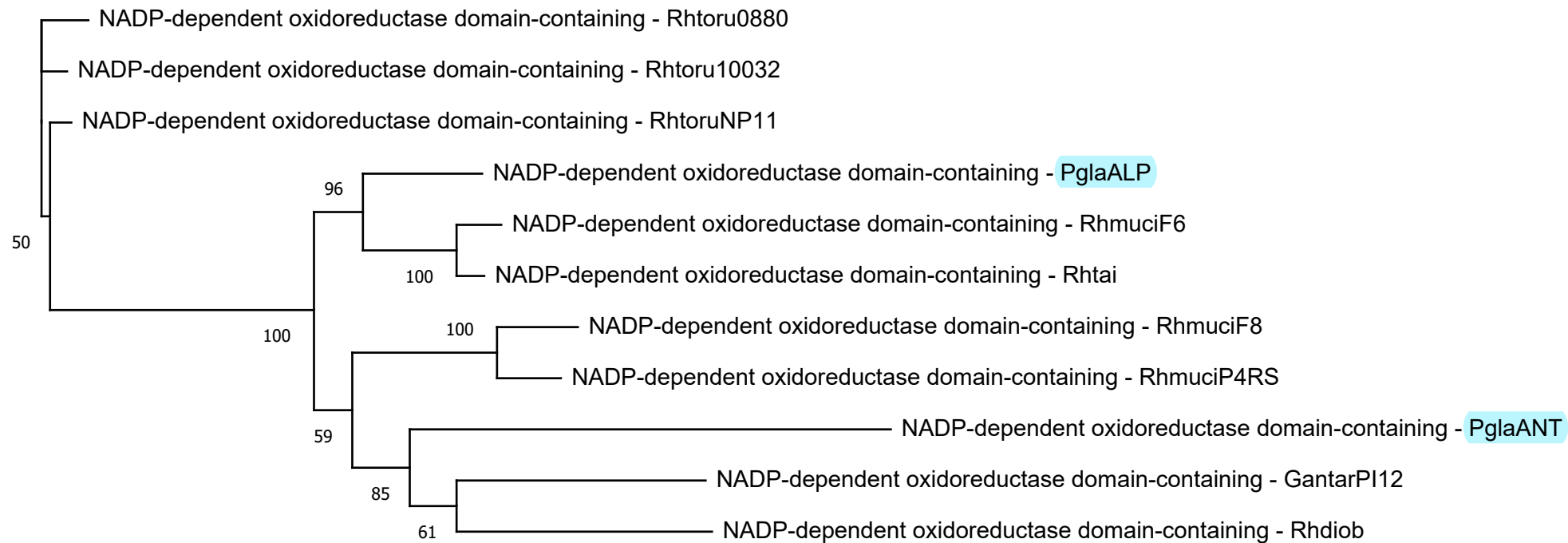

0.50

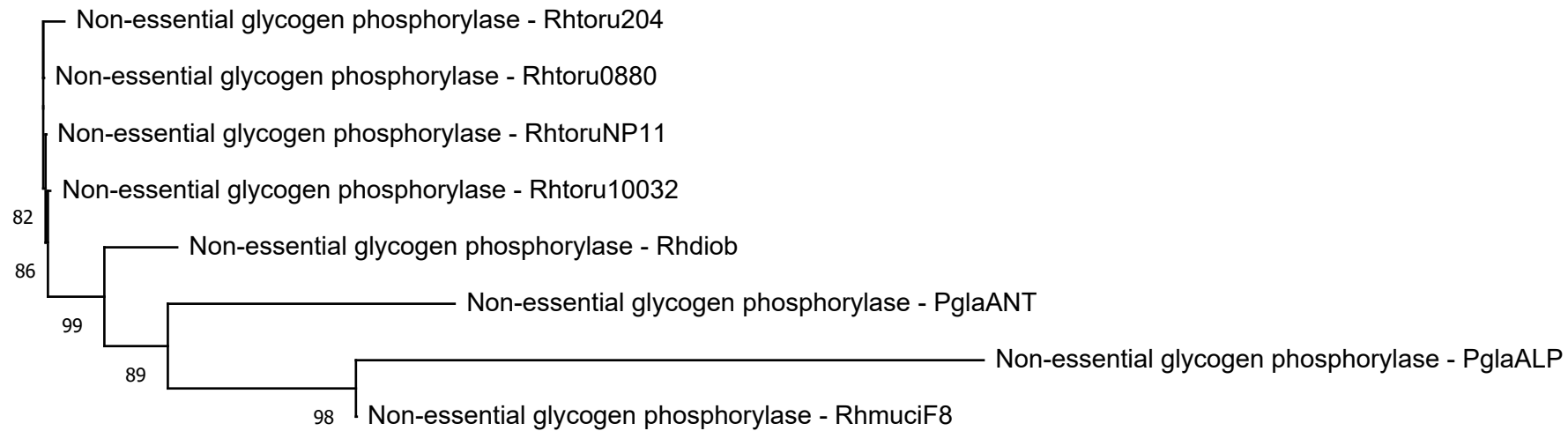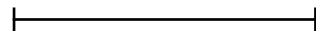

0.50

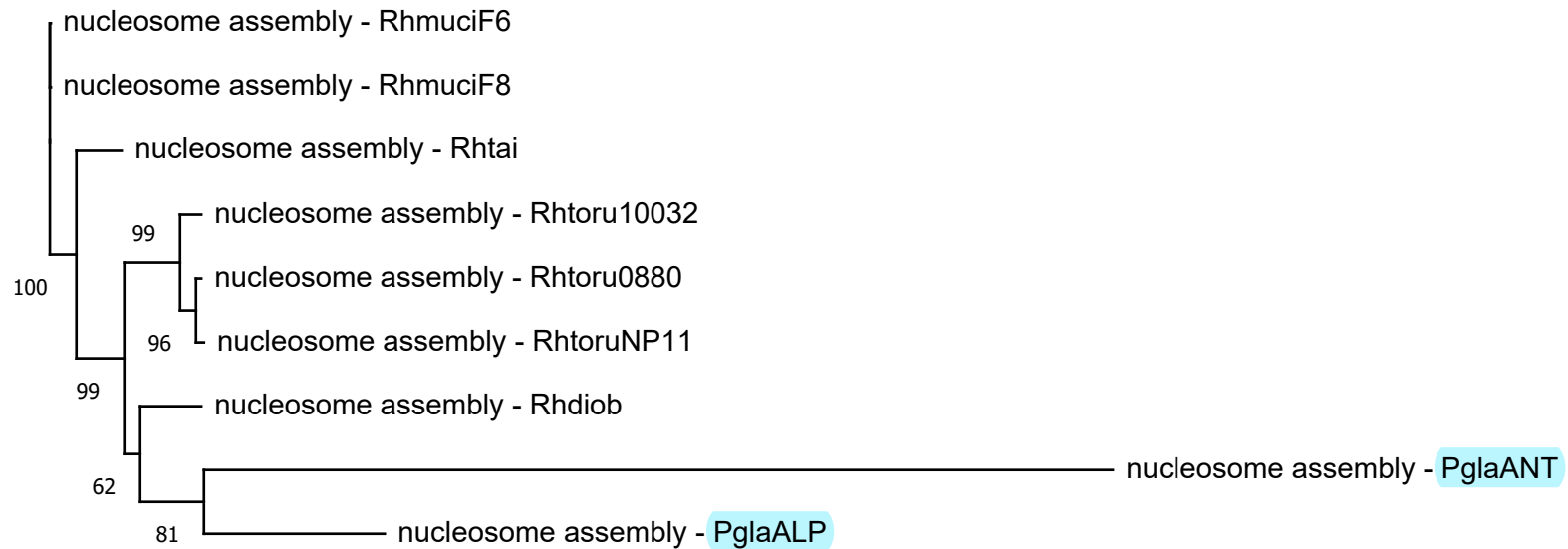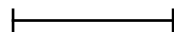

0.20

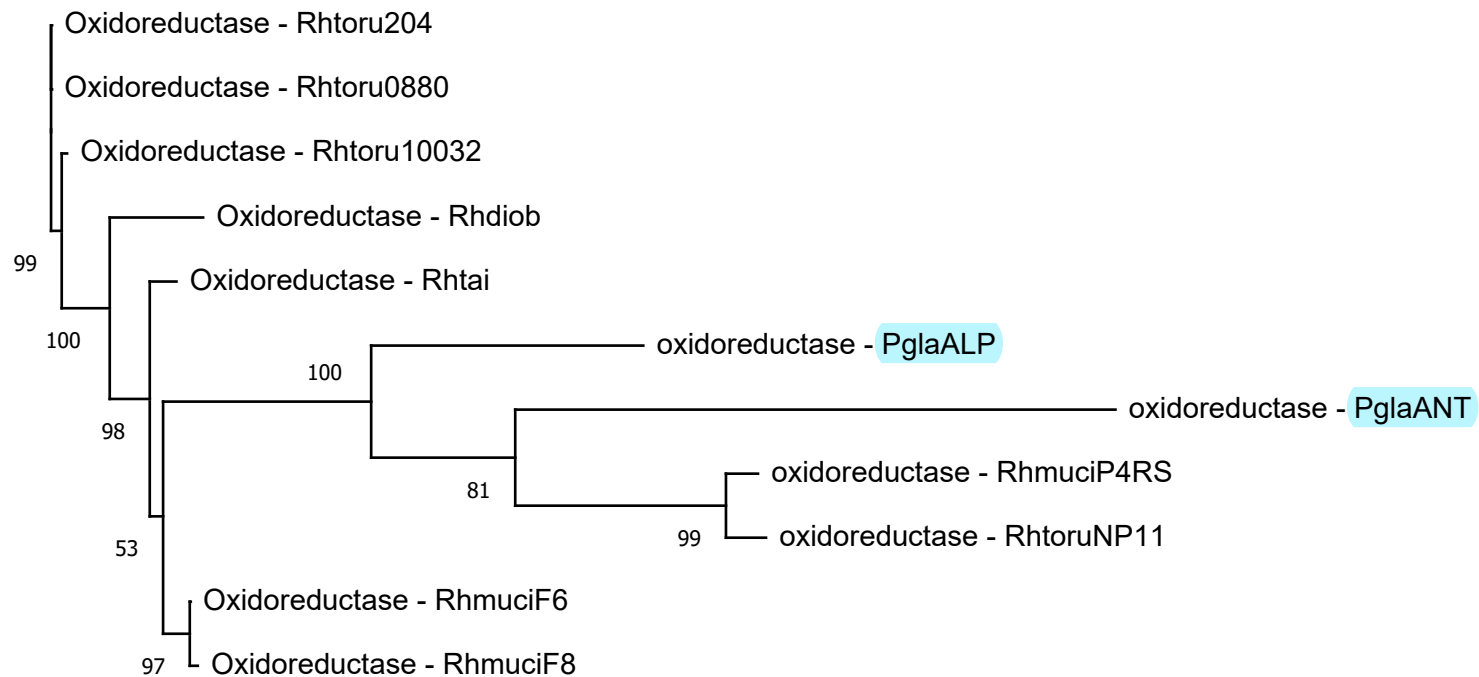

0.50

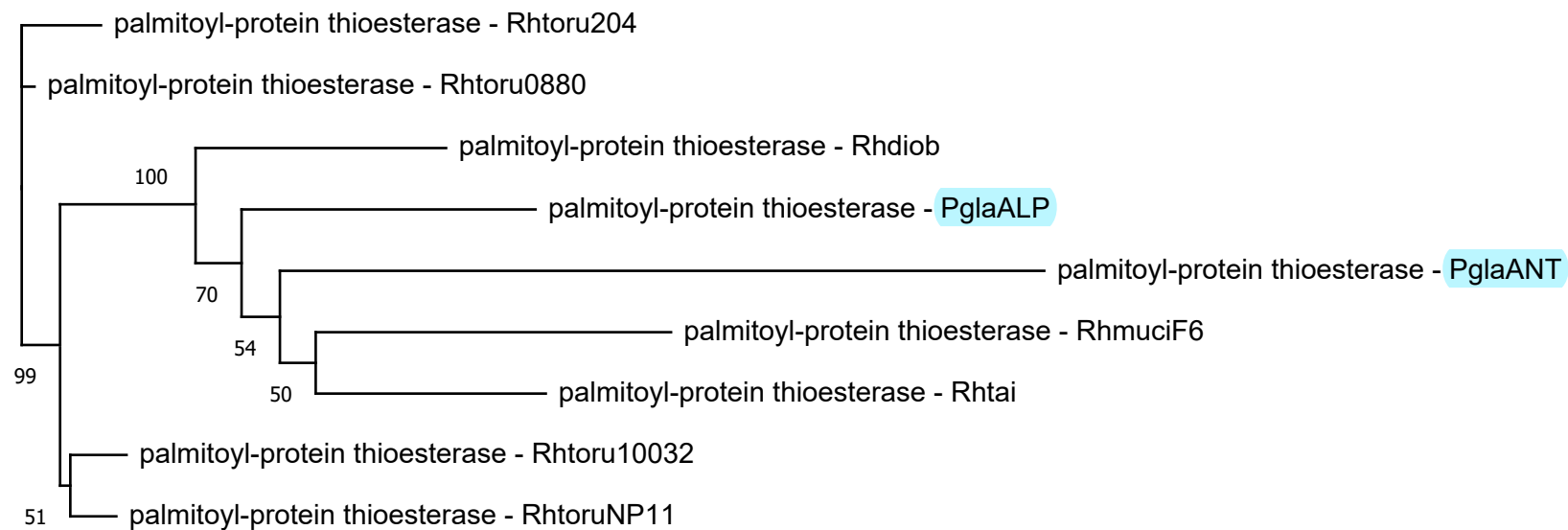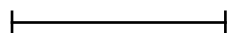

0.20

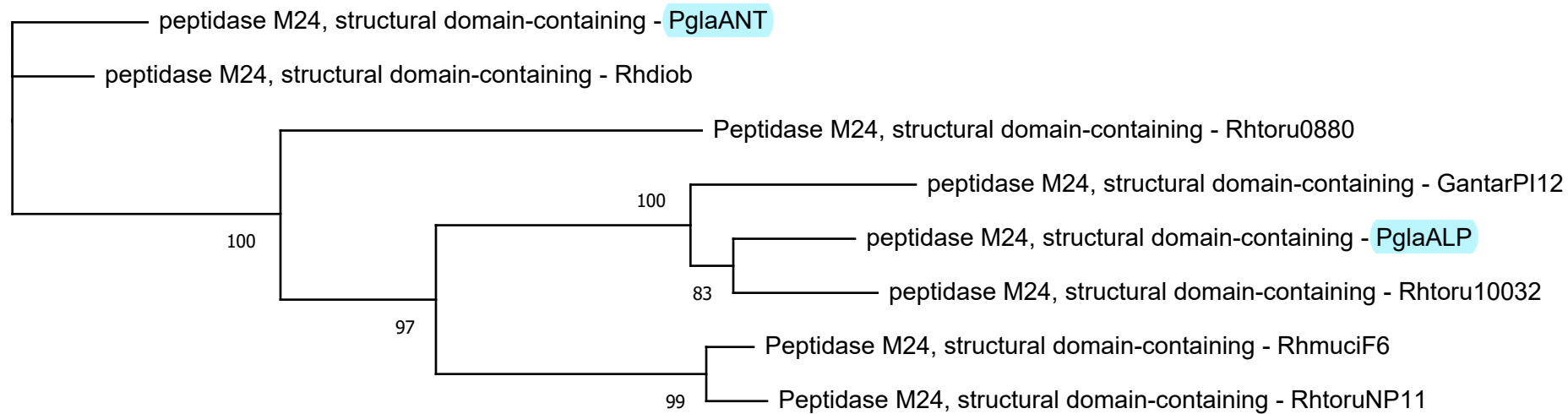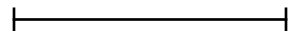

0.50

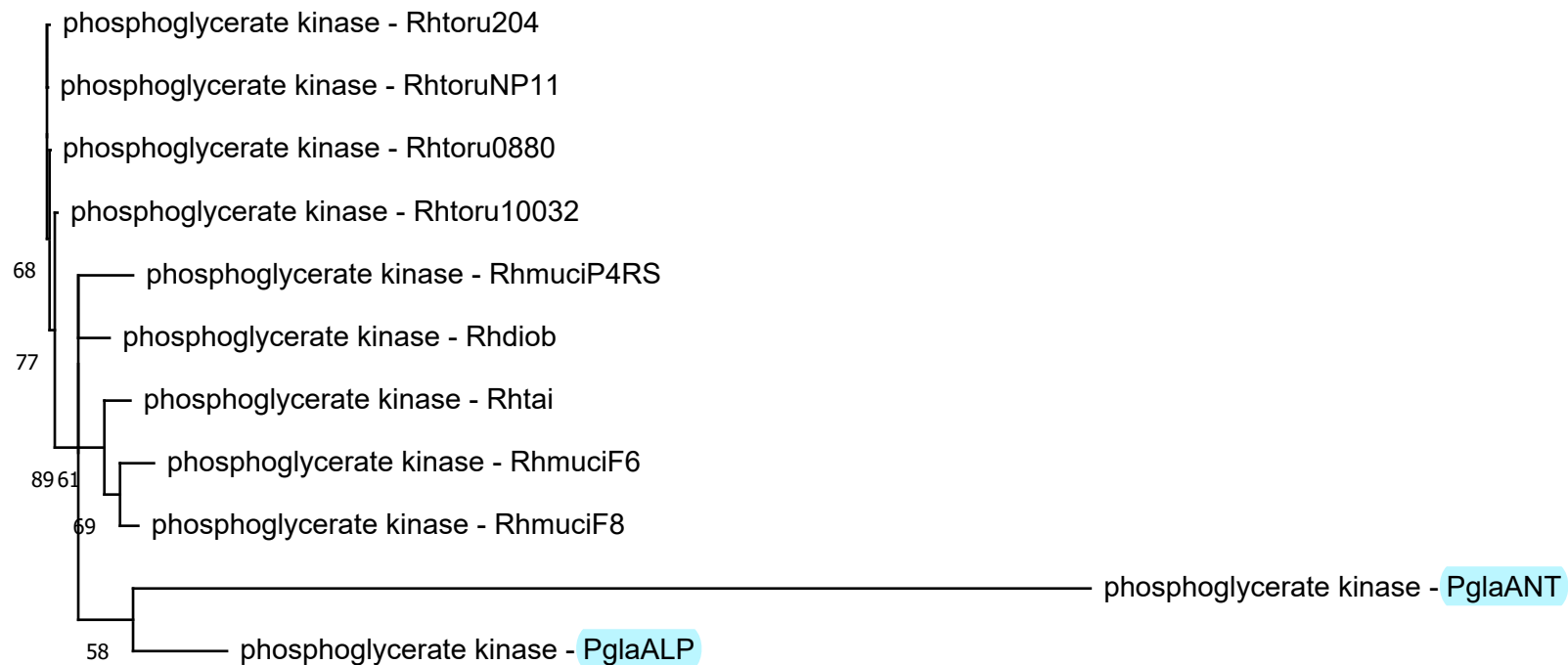

0.20

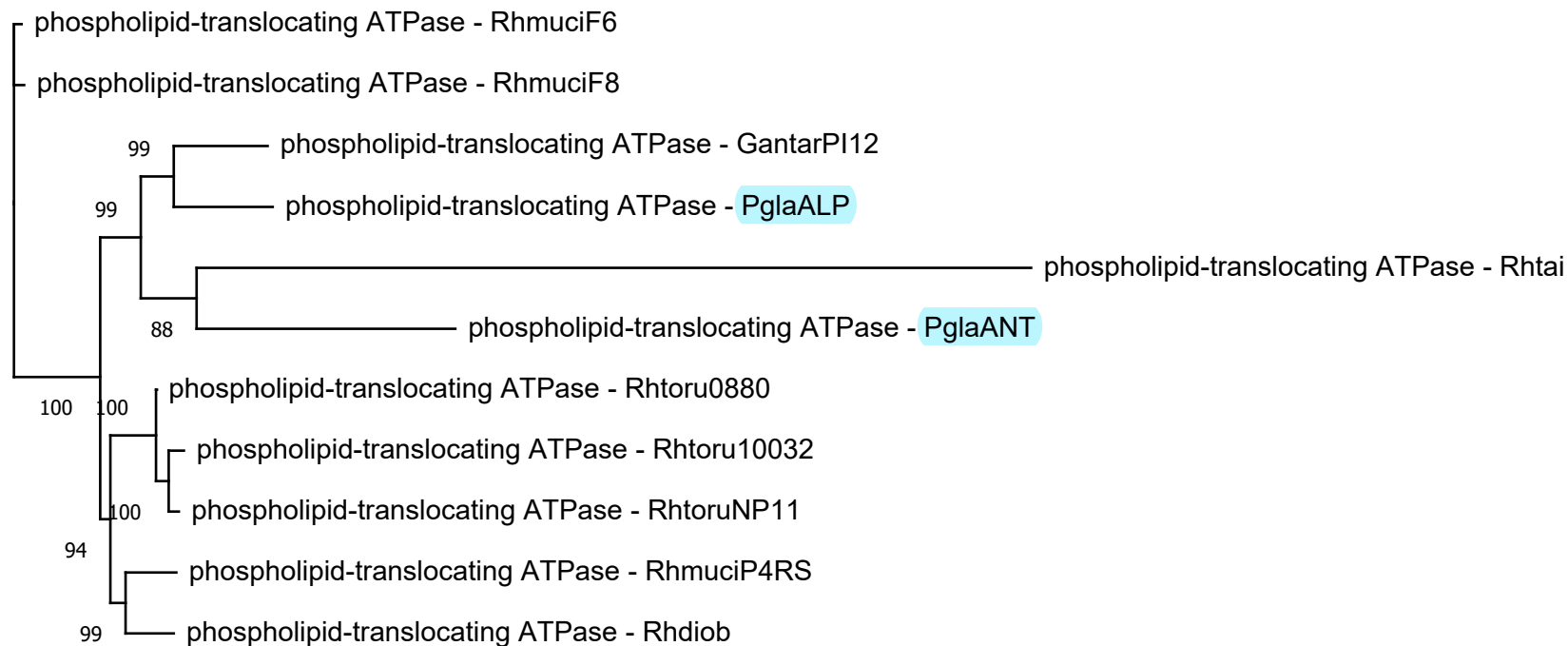

0.20

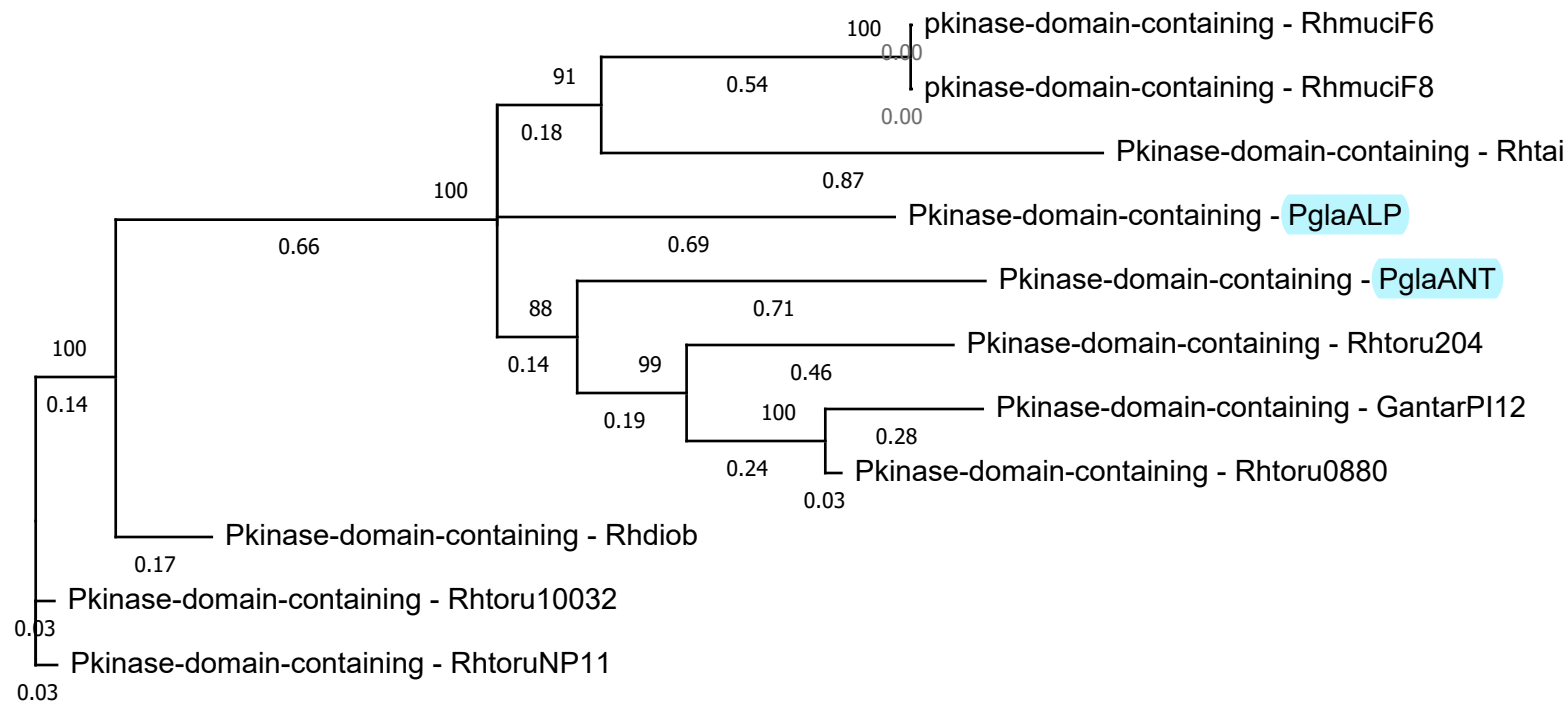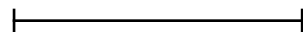

0.50

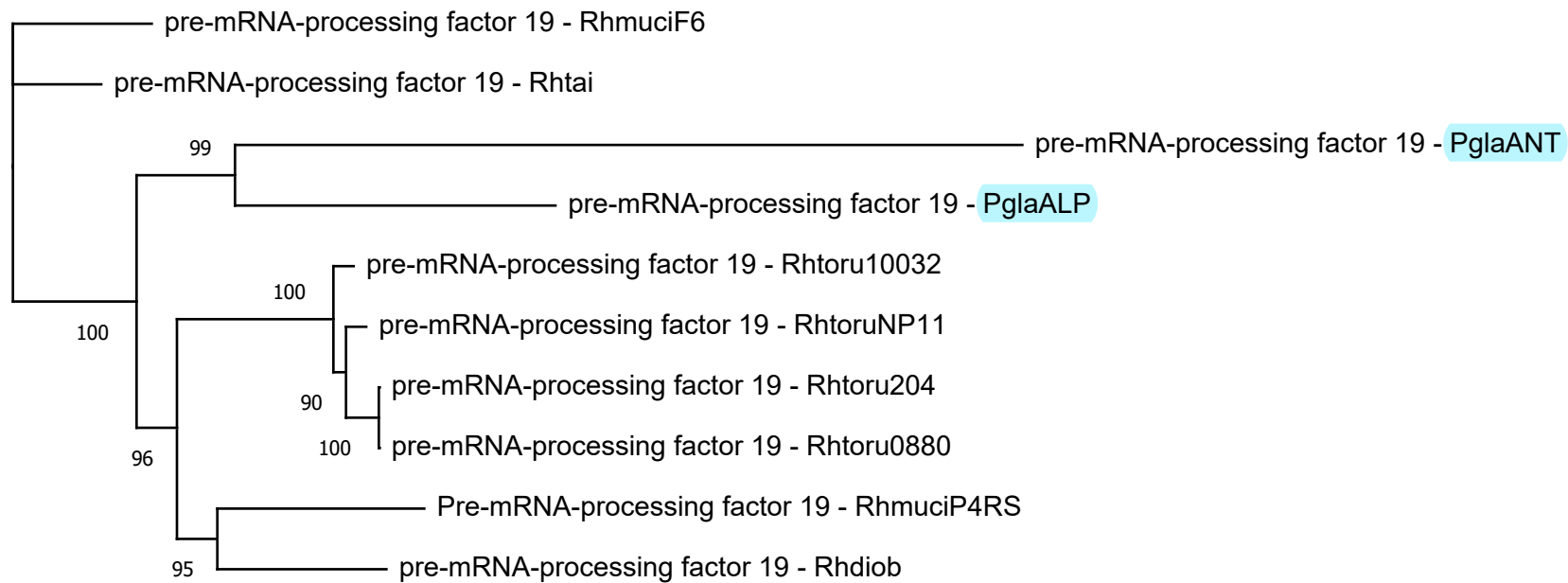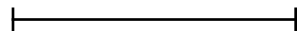

0.20

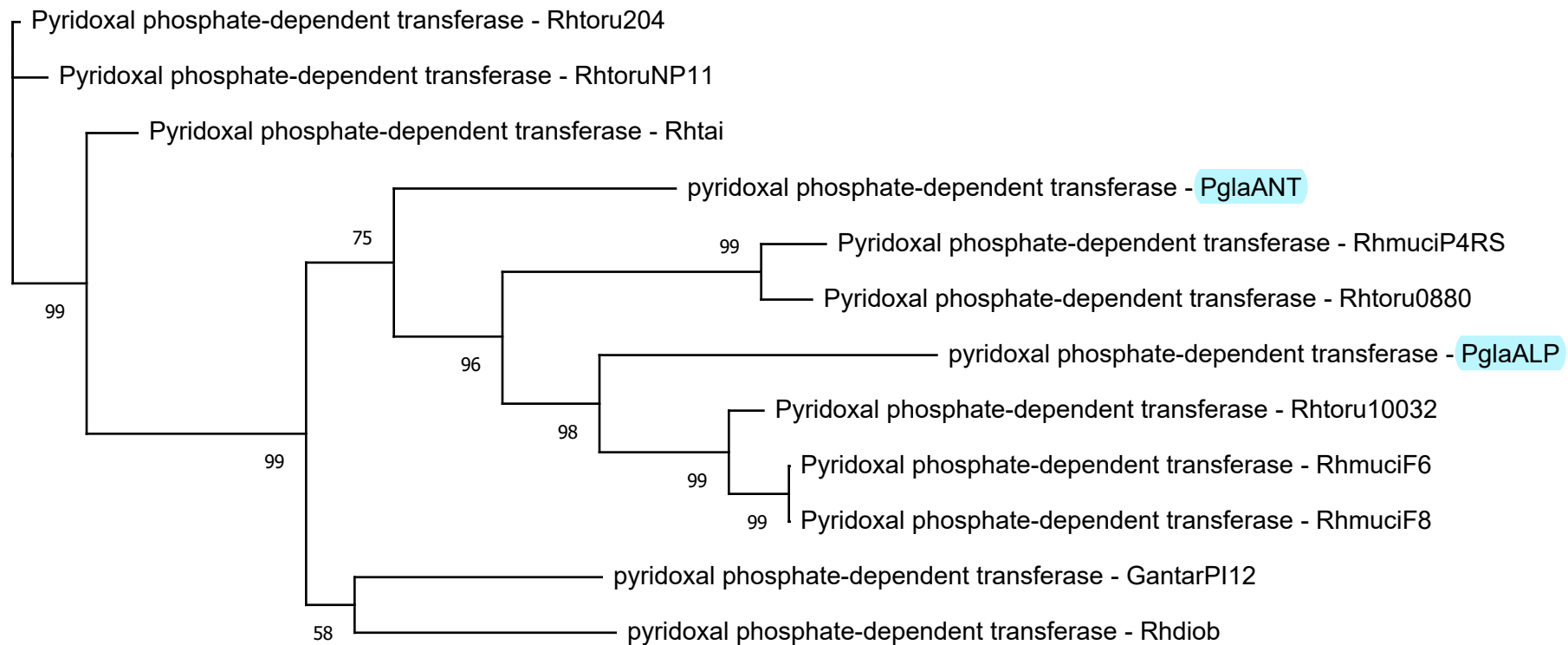

0.50

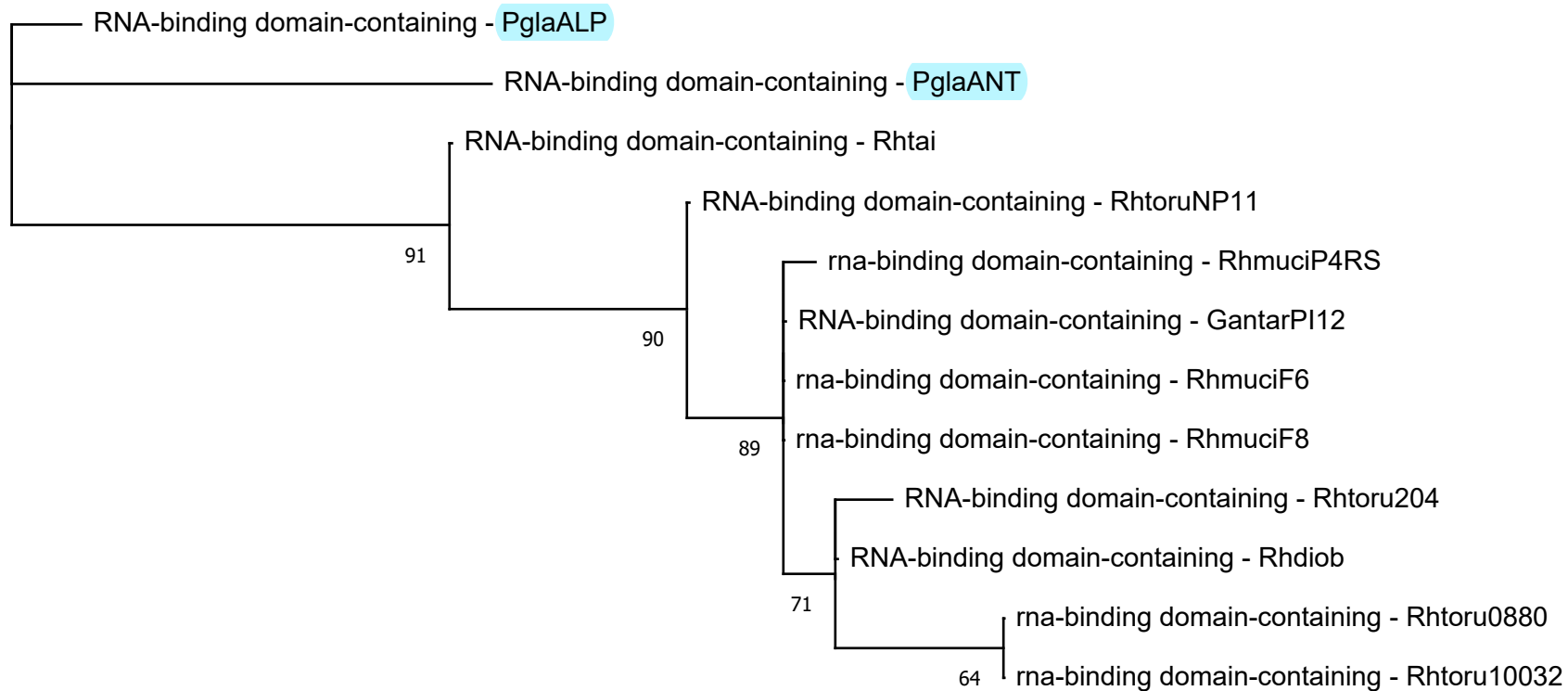

10.00

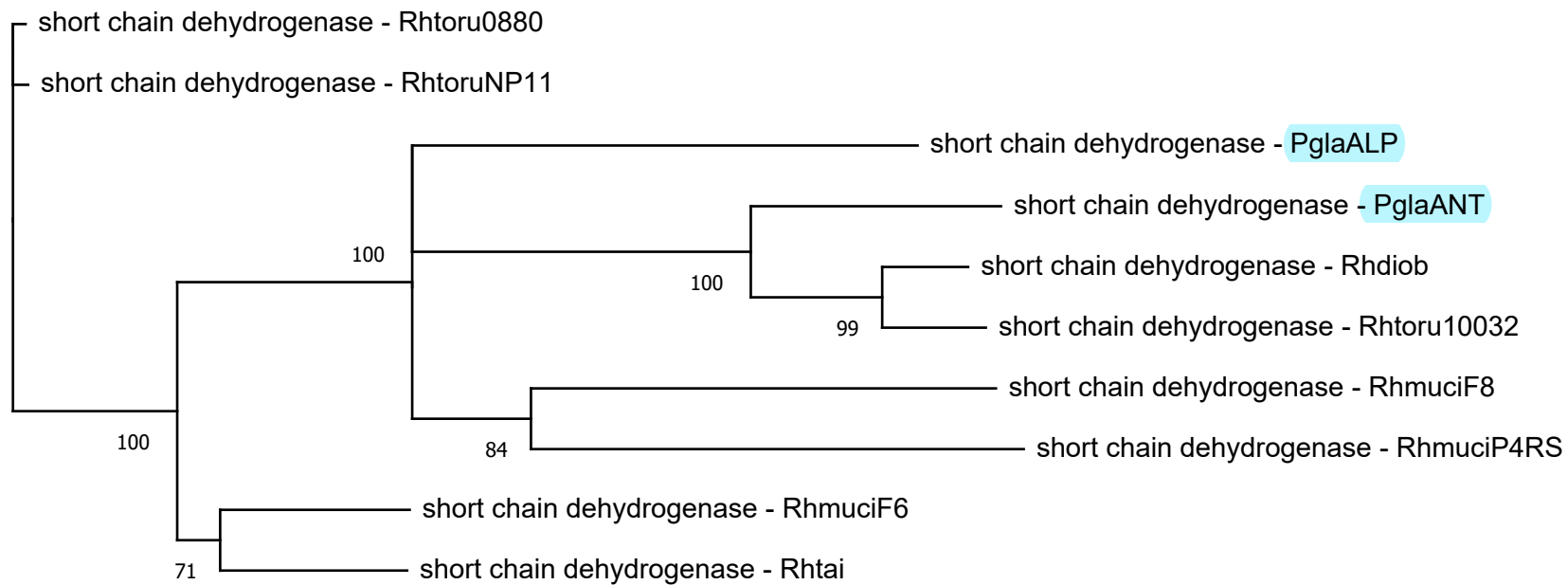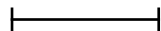

0.20

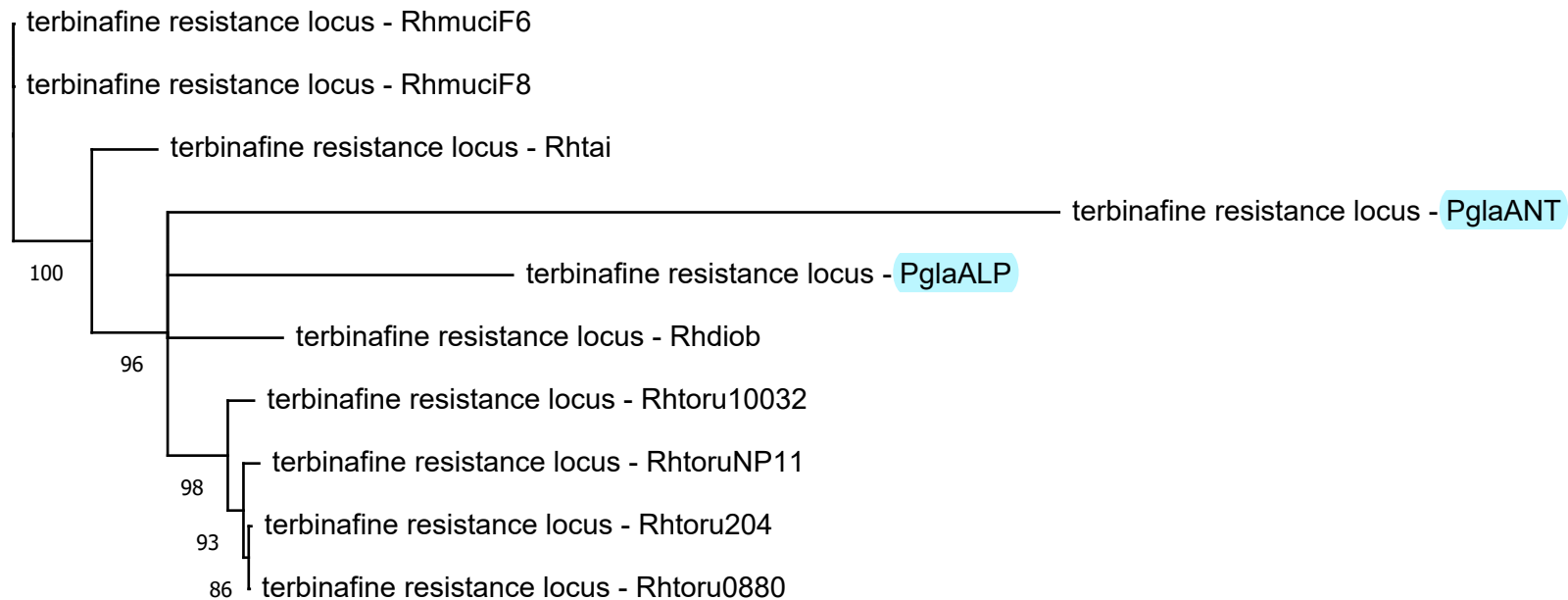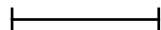

0.10

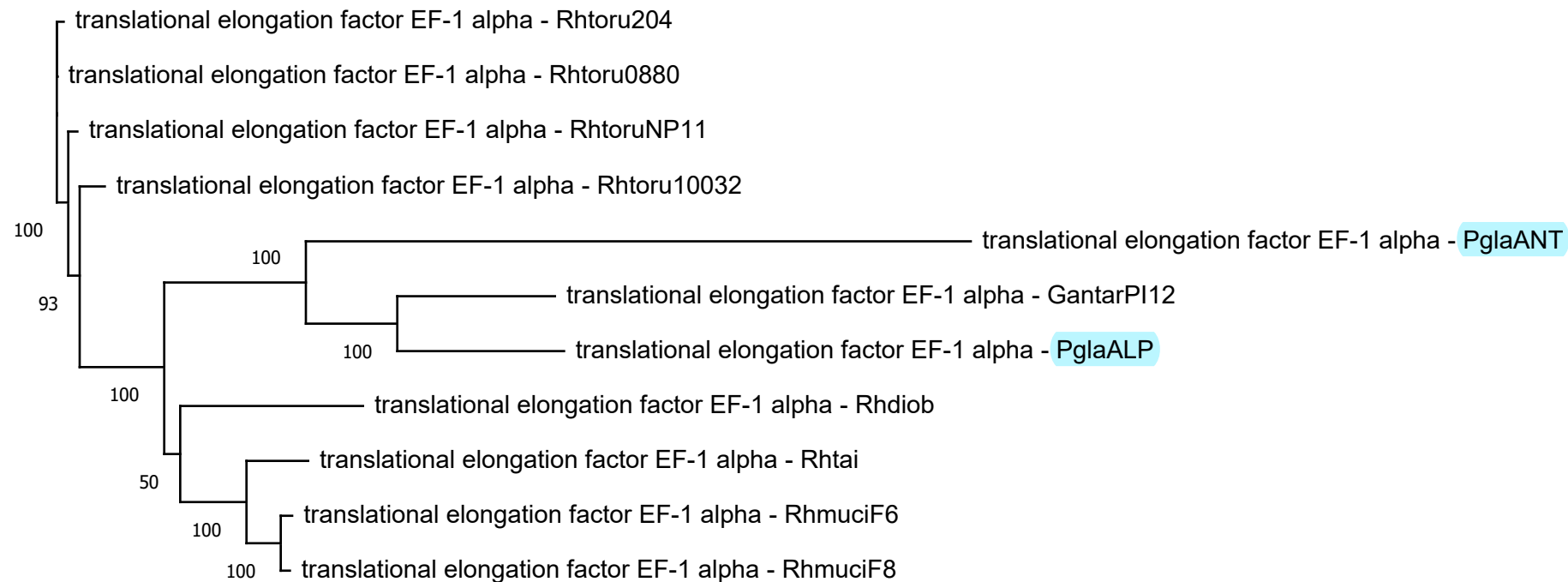

0.10

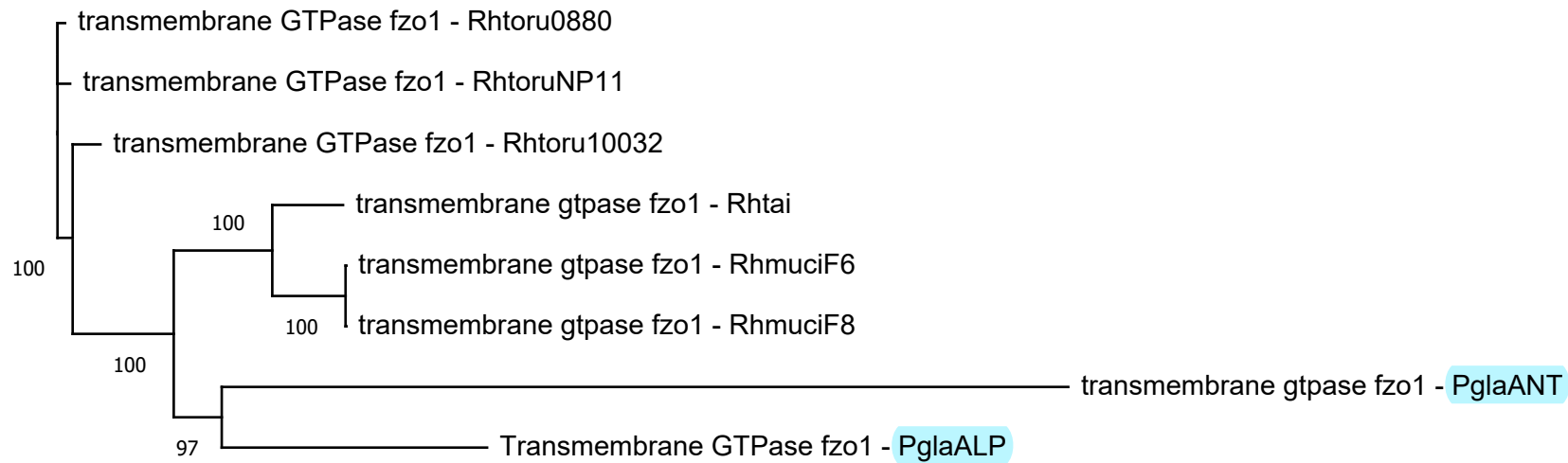

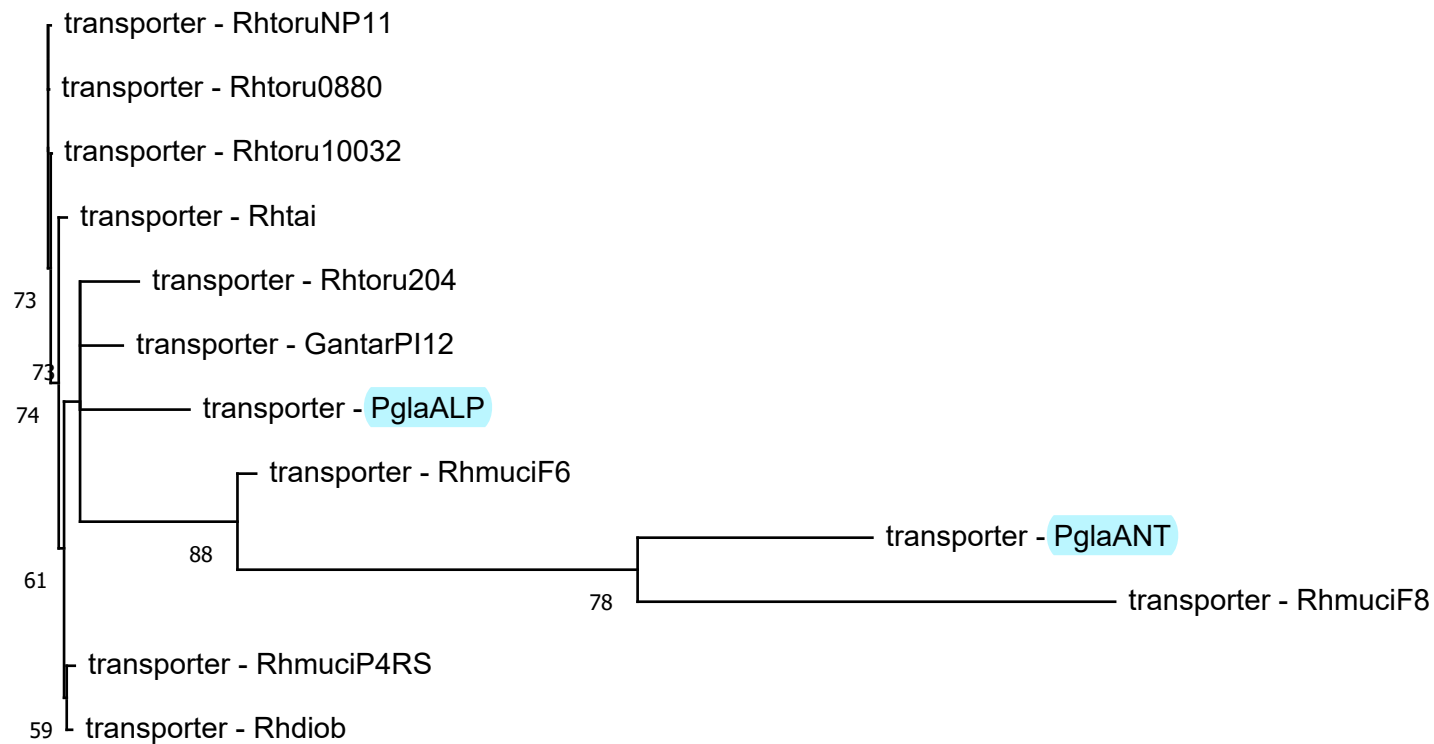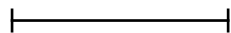

5.00

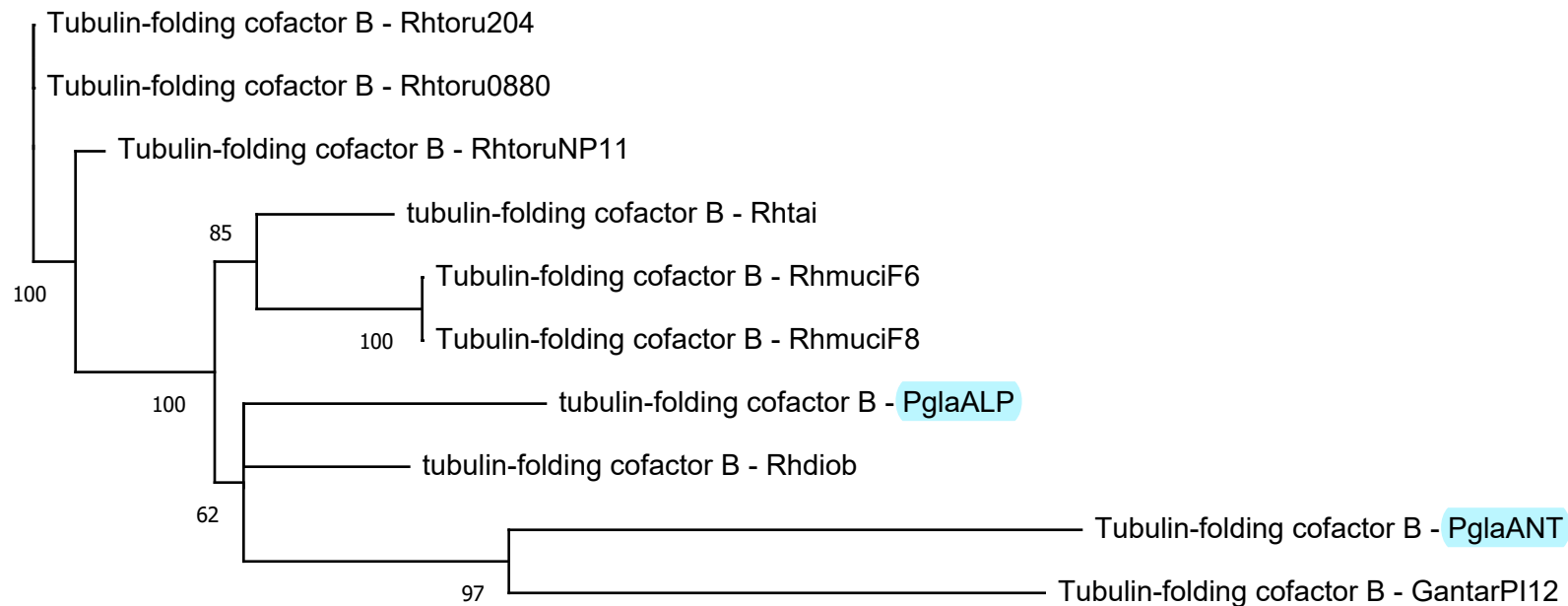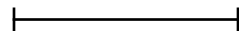

0.20

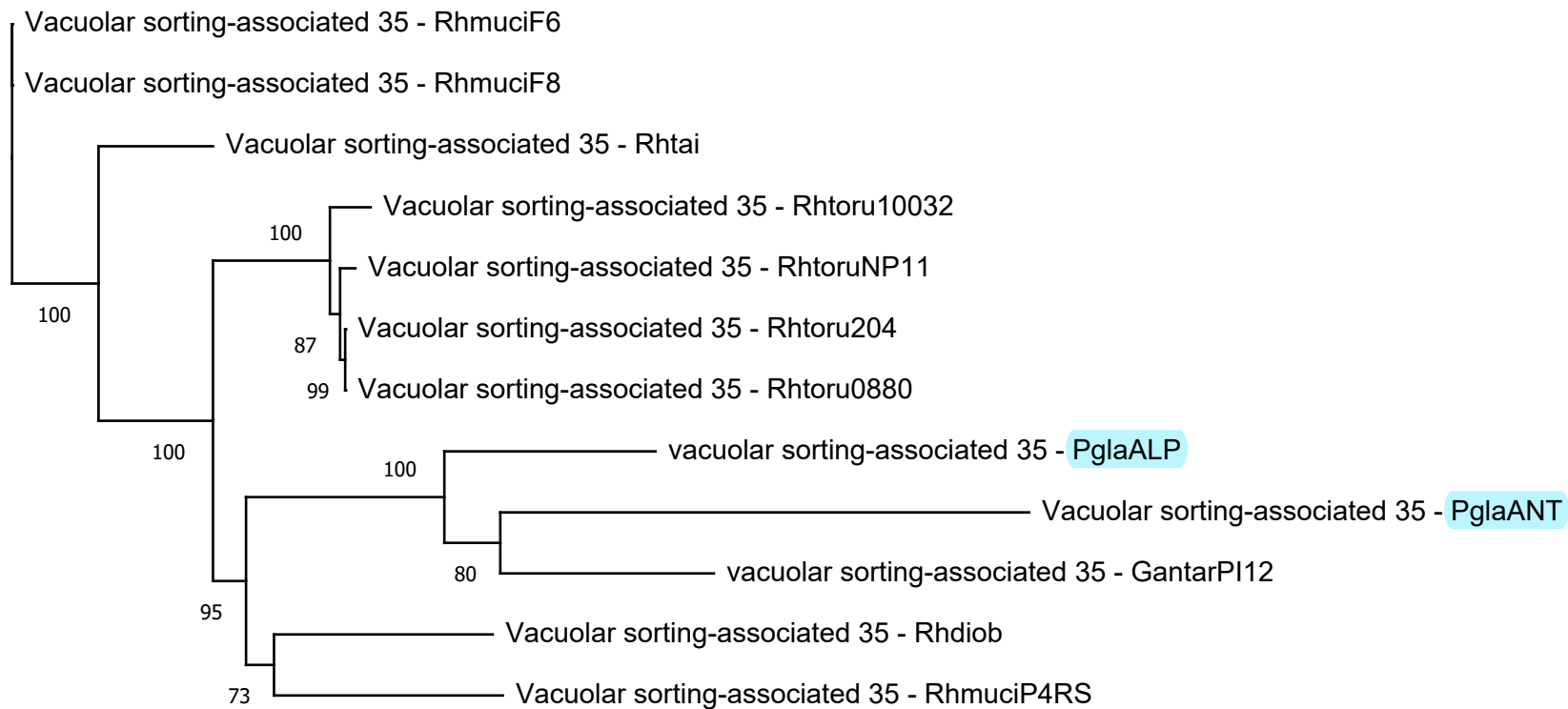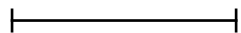

0.10

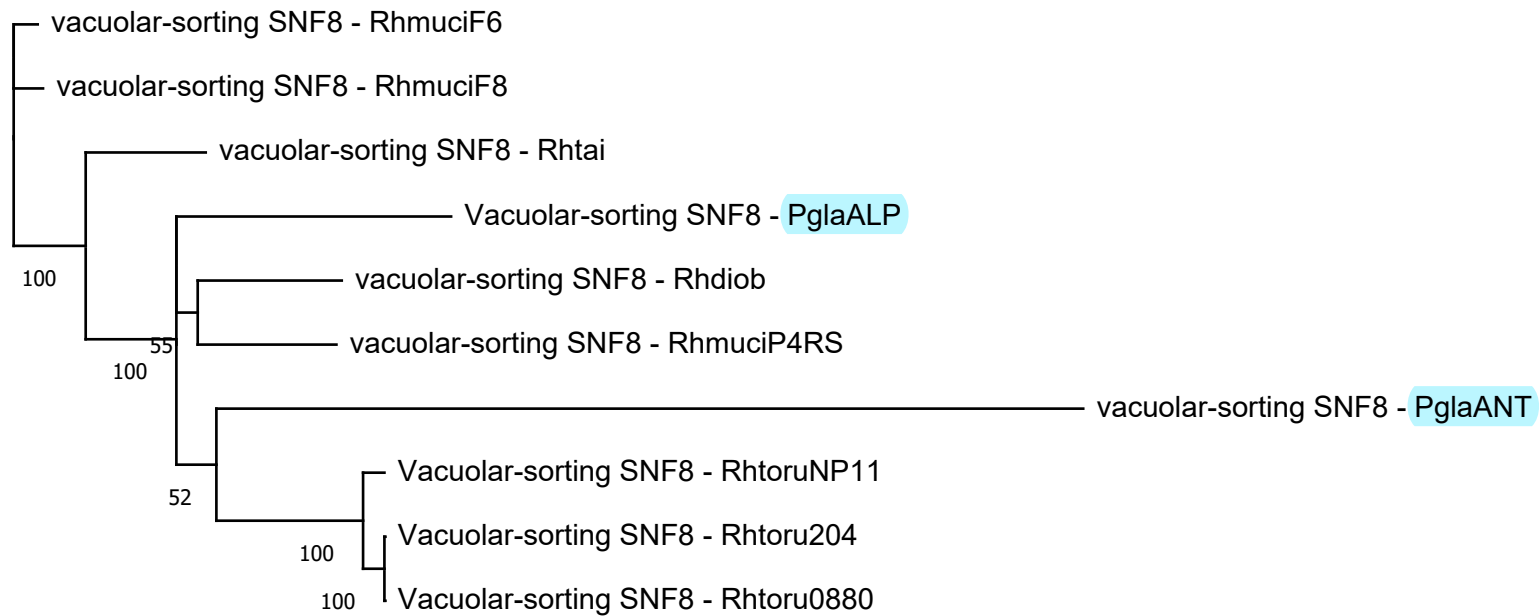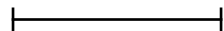

0.20

Supplement: Supplementary FIGURE S3 — Phylogenetic analysis for individual proteins used in multigene phylogeny analysis. The evolutionary history was inferred by using the Maximum Likelihood method and JTT matrix-based model. The percentage of trees in which the associated taxa clustered together is displayed next to the branches. [file Data_Sheet_3.PDF]
